# Concentrations of selected human milk components influence the infant oral microbiome to a greater degree than estimated intakes

**DOI:** 10.3389/fcimb.2026.1765736

**Published:** 2026-05-28

**Authors:** Roaa A. Arishi, Ali S. Cheema, Jacki L. McEachran, Zoya Gridneva, Phil Vlaskovsky, Isabella Norrish, Sabrina H. Bilston-John, Xiaojie Zhou, Ching Tat Lai, Matthew S. Payne, Donna T. Geddes, Lisa F. Stinson

**Affiliations:** 1 School of Molecular Sciences, The University of Western Australia, Crawley, WA, Australia; 2 Australian Breastfeeding + Lactation Research and Science Translation (ABREAST) Network, Perth, WA, Australia; 3 UWA Centre for Human Lactation Research and Translation, Crawley, WA, Australia; 4 Ministry of Education, Riyadh, Saudi Arabia; 5 The Kids Research Institute Australia, Nedlands, WA, Australia; 6 Department of Mathematics and Statistics, School of Physics, Mathematics and Computing, The University of Western Australia, Crawley, WA, Australia; 7 School of Biomedical Sciences, The University of Western Australia, Crawley, WA, Australia; 8 Division of Obstetrics and Gynaecology, The University of Western Australia, Crawley, WA, Australia

**Keywords:** antimicrobial proteins, breastfeeding, human milk, lactose, minerals, oral microbiome

## Abstract

**Background:**

Human milk is characterised by its complex composition, consisting of nutrient and bioactive components that play a crucial role in infant health. Although the infant oral cavity is directly exposed to these components during breastfeeding, their effects on the developing oral microbiome remains underexplored. This study aimed to assess associations between the concentrations and daily estimated intakes of human milk components (including minerals, lactose, and antimicrobial proteins) and the oral microbiome of exclusively breastfed infants.

**Methods:**

We profiled infant oral samples collected at 3 months of age using full-length 16S rRNA gene sequencing, alongside paired analyses of human milk components from 45 mother-infant dyads in the Western Australian BLOSOM cohort. Concentrations of milk lactose, antimicrobial proteins (AMPs), and micronutrients (16 components in total) were measured, and their daily estimated intakes were calculated based on 24-hour milk intake.

**Results:**

The composition of the infant oral microbiome was significantly associated with a number of AMPs and micronutrients, with concentration exerting a far stronger effect than estimated intakes. Lactose, the major sugar in human milk, was not associated with any feature of the infant oral microbiome. Both concentrations and estimated intakes of lactoferrin (P = 0.032 and P = 0.005, respectively), as well as estimated intakes of sodium and iodine (P = 0.041 and 0.022, respectively) were negatively associated with infant oral Shannon diversity. While some associations were consistent when both estimated intakes and concentrations were analysed, some appeared only in one analysis, suggesting differing mechanisms of action.

**Conclusion:**

These findings underscore the influence of human milk composition on the developing oral microbiome during early life, highlighting that local, concentration-driven mechanisms are the primary drivers of these effects.

## 1 Introduction

Exclusive breastfeeding is recommended for the first six months of life (World Health Organization (WHO), 2021), not only for its nutritional benefits but also for protective effects against infections, including gastrointestinal and upper- and lower respiratory tract infections (Duijts et al., 2009; Victora et al., 2016). Human milk a complex biofluid containing macronutrients, micronutrients, and a diverse array of bioactive compounds that contribute to immune development and microbial colonisation early in life (Dror and Allen, 2018). In addition, human milk contains numerous other bioactive factors, including lipids and components of the complement system, which are increasingly recognised for their roles in immune defence and host-microbe interactions in early life (Xu et al., 2024; Xu and Wan, 2025).While extensive research has shown that variation in human milk composition shapes the infant gut microbiome (Kim and Yi, 2020; Boudry et al., 2021; Pace et al., 2021; Kijner et al., 2022; Johnson et al., 2024), far less is known about how these components influence microbial communities in other mucosal sites directly exposed to milk, particularly the oral cavity.

The infant oral microbiome assembles rapidly after birth. In healthy infants, early oral communities are characterised by low alpha diversity and strong dominance of commensal genera, particularly non-mutans *Streptococcus* (Oba et al., 2020; Li et al., 2023). Community composition is initially influenced by determinants such as mode of delivery and intrapartum antibiotic prophylaxis (Li et al., 2005; Plonka et al., 2012; Gomez-Arango et al., 2017; Butler et al., 2022). As the oral environment matures – driven by tooth eruption, dietary transitions, and accumulating environmental exposures – *Streptococcus* relative abundance decreases and community diversity increases, with genera such as *Veillonella*, *Prevotella*, and *Haemophilus* becoming more established (Sulyanto et al., 2019; Oba et al., 2020; Xu et al., 2022). This successional trajectory means that both alpha diversity and community composition are inherently dynamic in early life, and sensitive to factors that may accelerate, delay, or perturb normal assembly (Sulyanto et al., 2019; Oba et al., 2020; Butler et al., 2022; Li et al., 2023).

The infant oral microbiome plays a critical role in oral health trajectories (Xiao et al., 2020), including the development of dental biofilms and future susceptibility to dental caries. Early-life oral microbial composition can influence biofilm structure, metabolic activity, and the later colonisation of cariogenic species, such as *Streptococcus mutans* (Dzidic et al., 2018). Epidemiological studies have reported associations between prolonged breastfeeding and increased risk of early childhood caries (Cui et al., 2017; Peres et al., 2017; Moynihan et al., 2019; van Meijeren-van Lunteren et al., 2021), yet the biological mechanisms underpinning these associations remain poorly understood. Given that the infant oral cavity is repeatedly and directly exposed to human milk during breastfeeding, it is plausible that milk-derived nutrients and antimicrobial proteins exert selective pressures on early oral microbial communities. While breastfeeding *per se* is known to shape the infant oral microbiome (Butler et al., 2022; Schulkers Escalante et al., 2025), it is important to understand how specific milk components influence the developing infant oral microbiome to better understand mechanistic links between breastfeeding, oral microbial ecology, and early-life caries risk.

In the present study, we selected key human milk components to examine in relation to the infant oral microbiome. These were chosen based on their abundance in milk and biological relevance to microbial colonisation in the infant oral cavity. Lactose, the dominant carbohydrate in human milk, is a major carbon source to which oral microbes are repeatedly exposed during breastfeeding and may influence microbial metabolism at mucosal sites. We also focused on highly abundant antimicrobial proteins (AMPs), namely lactoferrin, lysozyme, and secretory immunoglobulin A (sIgA), which may modulate microbial colonisation via their roles in innate and adaptive immune protection, iron sequestration, cell wall disruption, and immune exclusion. In addition, a panel of essential minerals and trace elements (including iron, zinc, iodine, selenium, calcium, and phosphorus) was selected as these components are required for microbial growth and enzymatic activity, but can also exert antimicrobial or biofilm-modifying effects depending on their concentration. Collectively, these components capture key nutritional and bioactive features of human milk with plausible pathways through which they may influence the structure of the developing infant oral microbiome.

When assessing the relationship between human milk components and the infant oral microbiome, it is important to assess both concentrations and total estimated intakes of each component. This is because milk estimated intake and milk composition concentrations vary significantly between individual mother-infant dyads (Arishi et al., 2025b). Therefore, two infants who receive milk with the same concentration of a particular component, but who consume different volumes of milk, are exposed to differing doses of that component. Thus, measurement of both concentrations and estimated daily intakes of these components are important for understanding the role of human milk in shaping the infant oral microbiome.

In the present study, we aimed to explore associations between key human milk components (concentrations and estimated daily intakes) and the oral microbiome in healthy, term, exclusively breastfed infants.

## 2 Materials and methods

### 2.1 Study design

The BLOSOM (Breastfeeding Longitudinal Observational Study of Mothers and Kids) cohort comprised 85 mother–infant dyads. For the present sub-study, 46 dyads had paired human milk composition and infant oral microbiome data at 3 months of age; one dyad was excluded due to mixed feeding, resulting in a final sample size of 45. Pregnant women in their third trimester (>30 weeks gestation) were recruited from the community and online networks. Eligible participants were healthy women without major pregnancy complications who planned to exclusively breastfeed for at least five months and continue up to 12 months. Exclusion criteria included maternal smoking, pregnancy complications (e.g., preterm labor, gestational diabetes), and infant conditions affecting growth or body composition. Participants provided written informed consent, and the study was approved by The University of Western Australia Human Research Ethics Committee (RA/4/20/4023, 7 March 2018). Sample and data collection.

Participant metadata was collected at the time of enrolment (third trimester of pregnancy) and at the sample collection time point. Collected metadata included maternal health history, demographic data, birth mode, infant feeding practices, and maternal and infant health.

Infant oral swabs and human milk samples were collected at 3 months postpartum. Mothers were provided with written instructions and used Copan E-Swabs to collect samples from their infant’s oral cavity (Becton, Dickinson and Company, Franklin Lakes, NJ, USA) and stored them in their home refrigerators for up to 18 hours before being transported on ice to the laboratory, where they were immediately aliquoted into sterile tubes (Sarstedt, Numbrecht, Germany) and stored at -80 °C until subsequent analysis. Oral swabs were vortexed for 5 seconds to elute the sample from the swab into the collection media before aliquoting.

### 2.2 Biochemical analysis of human milk

Milk samples collected at 3 months postpartum were used for human milk composition analysis. Assay detection limits, recovery rates, and coefficients of variation are provided in **Supplementary Tables 1**, **2**.

#### 2.2.1 Lactose

The lactose concentration was measured in skim milk samples using the Megazyme lactose kit (K-LOLAC, Megazyme, Wicklow, Ireland), as previously described (Mitoulas et al., 2002). Skim milk samples, standards, and qualitative control samples were analysed in duplicate. Briefly, the reaction solution (105 µL per well), containing buffer, NADP^+^/ATP, and distilled water, was prepared immediately prior to analysis according to the manufacturer’s instructions. Skimmed milk samples, diluted 1:50 in distilled water, along with standards and QC samples, were added (10 µL per well) to a 96-well microtiter plate containing 105 µL of the prepared reaction solution. Plates were mixed for 3 minutes at room temperature on a plate shaker (PST-60HL; BioSan SIA, Latvia), and the initial absorbance (A_1_) was recorded at 340 nm using a plate spectrophotometer (Enspire Multimode Plate Reader, Waltham, MA, USA). Subsequently, 2 µL of suspension (containing hexokinase, glucose-6-phosphate dehydrogenase, and 6-phosphogluconate dehydrogenase) was added to each well, mixed, and incubated for 10 minutes, after which absorbance (A_2_) was measured at 340 nm. Finally, 2 µL of suspension (β-galactosidase) was added, mixed, and incubated for 15 minutes, and absorbance (A_3_) was measured at 340 nm.

#### 2.2.2 Minerals

Mineral concentrations were measured in whole milk. Multi-element standard solutions containing copper (Cu), iron (Fe), magnesium (Mg), and zinc (Zn) (10 µg/mL each) and calcium (Ca), potassium (K), sodium (Na), and phosphorus (P) (1000 µg/mL each) were used as calibration standards. Internal standards were comprised of yttrium (Y) and scandium (Sc) solutions (µg/mL). All standards were diluted using 18.2 MΩ water (DDI). The standard range for Cu, Fe, Mg and Zn was 0 to 8 µg/mL; Na was 0 to 500 µg/mL; K, Ca and P was 0 to 250 µg/mL. 200 µL of whole milk was added to glass tubes, followed by 300 µL of 65% HNO_3_ and 100 µL of internal standards. After incubation at 110 °C for 1 hour, 1500 µL of distilled deionised (DDI) water was added. The samples were then filtered and analysed using inductively coupled plasma optical emission spectroscopy (ICP-OES). Additionally, the concentrations of iodine (I), selenium (Se), manganese (Mn), and molybdenum (Mo) were measured using inductively coupled plasma mass spectrometry as described previously (Bilston-John et al., 2021a).

#### 2.2.3 Antimicrobial proteins

Lysozyme concentrations were measured in skim milk samples using a modified turbidimetric assay (Selsted and Martinez, 1980) following the protocol outlined by Gridneva et al (Gridneva et al., 2016). Skim milk samples were diluted 1:10 in 0.1 M Na_2_HPO_4_/1.1 mM citric acid buffer (pH 5.8). Hen egg white lysozyme (EC 3.2.1.17; Sigma, St. Louis, MA, USA) was used to generate a six-point standard curve, with concentrations ranging from 0.00075 to 0.0125 g/L. Twenty-five microlitres of standards or diluted samples were added to a 96-well microplate (Greiner Bio-One, Frickenhausen, Germany), followed by 175 µL of a *Micrococcus lysodeikticus* suspension (0.075% w/v; ATCC No. 4698; Sigma, St. Louis, MA, USA). Plates were incubated at room temperature for 1 h, after which absorbance was measured at 450 nm. All samples and standards were analysed in duplicate, and mean values were used for subsequent statistical analyses.

Lactoferrin and sIgA concentrations were measured in skim milk samples using ELISA, following the methodology outlined by Tijssen et al (Tijssen, 1985). sIgA concentrations in skim milk were measured using a sandwich ELISA. Skim milk samples were diluted 1:600, and this dilution was also used for the quality control (QC). Ninety-six-well high-binding microtitre plates were coated with 250 µL per well of anti-human sIgA capture antibody and incubated overnight at 4 °C. After discarding the capture antibody solution, 200 µL per well of blocking solution was added for 30 minutes at room temperature. Plates were then washed three times with wash buffer. Seven-point standard curves for sIgA (0–320 ng/mL) were prepared, and standards, samples, and QC samples were added in duplicate (200 µL per well) and incubated for 1 hour at 37 °C. Following three washes with wash buffer, 200 µL per well of detection antibody was added and incubated for 1 hour at 37 °C. Plates were subsequently washed three times with wash buffer and once with deionized water, after which 200 µL per well of horseradish peroxidase (HRP) solution was added and incubated in the dark at room temperature for 30 minutes. The color reaction was stopped by adding 100 µL per well of 1 M sulfuric acid, and absorbance was measured at 450 nm using a microplate reader. Mean values of duplicate wells were used for subsequent analyses.

Lactoferrin concentrations in skim milk were measured using a sandwich ELISA. Seven-point standard curves were prepared for lactoferrin, with concentrations ranging from 0 to 0.2 g/L. At the time of analysis, skim milk samples were thawed and diluted 2,000,000-fold in reagent diluent. Ninety-six-well microplates were coated with 100 µL per well of capture antibody solution and incubated overnight at 4 °C. Plates were washed three times with wash buffer before adding 250 µL per well of blocking buffer, followed by a 2-hour incubation at room temperature. After another three washes, 100 µL of standards or diluted samples were added to each well and incubated for 2 hours at room temperature. Plates were then washed three times, and 100 µL per well of detection antibody solution was added and incubated for 1 hour at room temperature. Following three additional washes, 100 µL of HRP solution was added and incubated for 30 minutes at room temperature. Plates were washed again, and 100 µL of Substrate A/B solution was added for 5 minutes at room temperature in the dark, after which the reaction was stopped with 100 µL stop solution. Absorbance was read at 450 nm using a microplate reader. All samples and standards were analysed in duplicate, and mean values were used for quantification.

### 2.3 24-h milk intake

Infant milk intake was measured at the three month time point using the 24-hour milk profile protocol as previously described (Arthur et al., 1987). Briefly, mothers weighed their infants before and after each feed using calibrated electronic infant scales (± 2 g) (Electronic Baby Weigh Scale, Medela Inc., McHenry, IL, USA). Human milk intake, in grams, was calculated by subtracting the pre-feed weight from the post-feed weight, then converted to millilitres using the density of human milk (1.03 g/mL). 24-hour milk intakes at three months were considered representative of intake levels during the exclusive breastfeeding period, as infant milk intake does not significantly vary from one to six months (Kent et al., 2013). Milk intake data was available for 42 of the 45 participants.

### 2.4 Estimated daily intake of human milk components

Estimated daily intakes of human milk components (g) were determined by multiplying the concentration of each component (g/L) by volume of milk intake per 24 hours.

### 2.5 Infant oral microbiome analysis

#### 2.5.1 DNA extraction

DNA extraction from the cell pellet was carried out using the QIAGEN MagAttract Microbial DNA Isolation Kit (QIAGEN, Hilden, Germany). Swabs were vortexed for 5 seconds to release cells. The swab media was then centrifuged at 40,000 × *g* for 5 minutes at 4 °C, and the supernatant was carefully removed. DNA was extracted from the resulting cell pellet using the QIAGEN MagAttract Microbial DNA Isolation Kit, which incorporates both chemical and mechanical lysis. A negative extraction control (reagents only) was included with each batch.

#### 2.5.2 16S rRNA gene amplification and sequencing

The full-length 16S rRNA gene was amplified using asymmetrically barcoded primers 27F and 1492R. All PCR reagents were decontaminated using the ArcticZymes PCR Decontamination Kit prior to use. PCR reactions (30 µL each) contained 0.75 µL each of DTT and dsDNase (ArcticZymes PCR Decontamination Kit), 0.3 µM of forward and reverse barcoded primers, 1X AccuStart II ToughMix, 3 µL of nuclease-free water, and 6 µL of template DNA. All master mix components were treated with the ArcticZymes PCR Decontamination Kit prior to template addition. Each batch included a no-template control. Cycling conditions were: initial denaturation at 94 °C for 3 minutes, followed by 35 cycles of 94 °C for 30 seconds, 52 °C for 30 seconds, and 72 °C for 2 minutes, with a final extension at 72 °C for 5 minutes. Amplicon presence and size were verified using the QIAxcel capillary gel electrophoresis system with a DNA High-Resolution cartridge. Amplicons were normalised, pooled, and purified using the Machery Nagel NucleoMag^®^ NGS Clean−up and Size Select kit. Purified amplicon pools were sequenced at the Australian Genome Research Facility (AGRF). Briefly, raw sequencing data were processed on a PacBio Sequel II using the CCS algorithm (SMRT Link v12.0.0) to generate HiFi reads, retaining only reads with ≥ 3 full passes and predicted accuracy ≥ Q20. The resulting HiFi reads had a median quality of Q32–Q33, mean read passes of 18-19, and mean lengths of 1,520–1,533 bp across all pools.

#### 2.5.3 Sequencing data processing

Mothur v.1.48.0 was used to process full-length 16S rRNA gene sequences (Schloss et al., 2009). Alignment to the SILVA reference alignment v132 was performed after raw sequences were filtered for length (1336–1743 bp) and homopolymer content (≤9) (Quast et al., 2013). Alignment quality was assessed using summary statistics in mothur, and sequences were screened based on alignment start and end positions to remove poorly aligned reads. Chimeric sequences were eliminated using VSEARCH (Rognes et al., 2016). Initial classification using the SILVA taxonomy database (v132) was performed to screen out non-bacterial sequences (Quast et al., 2013) using a confidence threshold of 80. Sequences were then clustered into operational taxonomic units (OTUs) at a 97% similarity threshold. OTU clustering was selected as a conservative approach to characterise community-level patterns within this consistently processed dataset, minimising over-splitting of taxa while accounting for intragenomic variation. We acknowledge that, unlike ASVs which have better cross-platform reproducibility, OTU clustering is pipeline-dependent. As such, raw sequence data has been publicly deposited (NCBI SRA accession number PRJNA1186108) enabling future reanalysis using alternative pipelines should cross-study comparisons be desired. BLAST was used to further classify highly abundant OTUs (average relative abundance >0.5%; n=15) to species level against the NCBI 16S rRNA gene database, with a minimum cutoff of 97% sequence identity and 99% sequence coverage (Altschul et al., 1997) (**Supplementary Table 3**). For each OTU representative sequence, matches to complete genome sequences or full-length 16S rRNA gene sequences were preferred over short-read entries. Where BLAST returned multiple equally strong matches across different species, assignments were reported as *[Genus]* sp. Final species assignments reflect the consensus of the top 100 BLAST hits rather than the single top hit in isolation, to avoid spurious assignments driven by marginal identity differences. Sequence matches generally showed high identity and coverage; however, these annotations reflect similarity to reference sequences and were therefore used to aid biological interpretation rather than to infer definitive species-level classification. Sequences recovered from the negative extraction and template controls are reported in **Supplementary Table 4**. Sub-sampled OTU-level data (2882 reads per sample) were used for all alpha and beta diversity analyses to standardise sequencing depth across samples. OTU abundance counts were separately centre log-ratio (CLR) transformed, with a pseudocount of 1 added to handle zeros, for compositional analyses and statistical testing. Relative abundance data were calculated from raw counts for visualization and descriptive summaries only, and were not used for differential abundance analyses.

#### 2.5.4 Statistical methods

Participant characteristics were summarised with means and standard deviations, and counts and proportions, as appropriate. The study included all mother-infant dyads from the BLOSOM cohort who were exclusively breastfeeding and had complete paired human milk and oral microbiome data at 3 months. As this was an exploratory analysis based on available complete cases, no *a priori* power calculation was performed. The sample size was determined by the availability of complete data from exclusively breastfeeding dyads within the BLOSOM cohort at this time point. *Post-hoc* power analyses were conducted using the pwr package (v.1.3.0) in R. Effect sizes were calculated as Cohen’s f², derived from the marginal increment in R² attributable to each milk component exposure above the covariate-only model (f² = (R²_full − R²_covariates)/(1 − R²_full)). Power was calculated at α = 0.05 for sample sizes of n=45 (concentration models) and n=42 (intake models), with one numerator degree of freedom reflecting the addition of a single exposure term. Results are reported in **Supplementary Tables 9**, **10**. Associations between human milk components (concentrations/estimated daily intakes) and the infant oral microbiome were assessed using covariate-adjusted linear regression models using lm function from the stats package (version 4.3.1) package in R (v. 4.3.0) (R Core Team, 2023). Covariates included delivery mode, presence of siblings, maternal pre-pregnancy BMI, and pacifier use (and time of milk collection for iron), and these were selected based on prior findings in this cohort demonstrating significant associations with infant oral microbiome beta diversity (Arishi et al., 2025a). Microbial outcomes included CLR-transformed abundance of the 15 OTUs meeting the relative abundance threshold, OTU-level richness, and Shannon diversity. Explanatory variables were the concentrations or estimated intakes of each milk component: lactose, minerals (Ca, Cu, Fe, I, K, Mg, Mn, Mo, Na, P, Se, Zn), and antimicrobial proteins (lactoferrin, lysozyme, sIgA). Given that intake data were available for only a subset of 42 mothers, concentration (n=45) and intake (n=42) models were performed separately. Model assumptions were assessed using examination of residual diagnostics, including Q-Q plots and residual versus fitted value plots. Homoscedasticity was assessed by inspection of residual versus fitted value plots. While minor deviations from normality were observed for some models, linear regression is generally robust to moderate departures from normality at this sample size, and no substantial violations of homoscedasticity were detected. Accordingly, no additional transformations were applied.

Associations between human milk components and infant oral microbiome beta diversity were assessed using individual PERMANOVA models for each milk component exposure, with a consistent *a priori* covariate set applied throughout (delivery mode, presence of siblings, maternal pre-pregnancy BMI, and pacifier use). Models were implemented using the adonis2 function in the vegan package (v 2.6.4) (Dixon, 2003), with Aitchison distances calculated from CLR-transformed OTU abundances and 999 unrestricted permutations. Formal dispersion testing via betadisper was not performed as this approach is designed for categorical exposures and not directly applicable to continuous exposures. Instead, ordination plots were visually inspected for evidence of heterogenous dispersion across the range of each exposure. To account for multiple testing across milk component exposures, P-values from the PERMANOVA models were adjusted using the Benjamini–Hochberg false discovery rate (FDR) method (**Supplementary Tables 5**, **6**).

Effect sizes (ß) and 95% confidence intervals are reported throughout. To control the false discovery rate, all P-values from the covariate-adjusted linear regression models were corrected using the Benjamini-Hochberg (BH) procedure, applied separately to concentration and intake analyses (n=45 and n=42, respectively), with all milk component exposures and microbial outcomes within each analysis type treated as a single hypothesis family. BH corrected P-values are presented throughout the text, and uncorrected values are reported in **Supplementary Tables**.

## 3 Results

**Figure 1:**
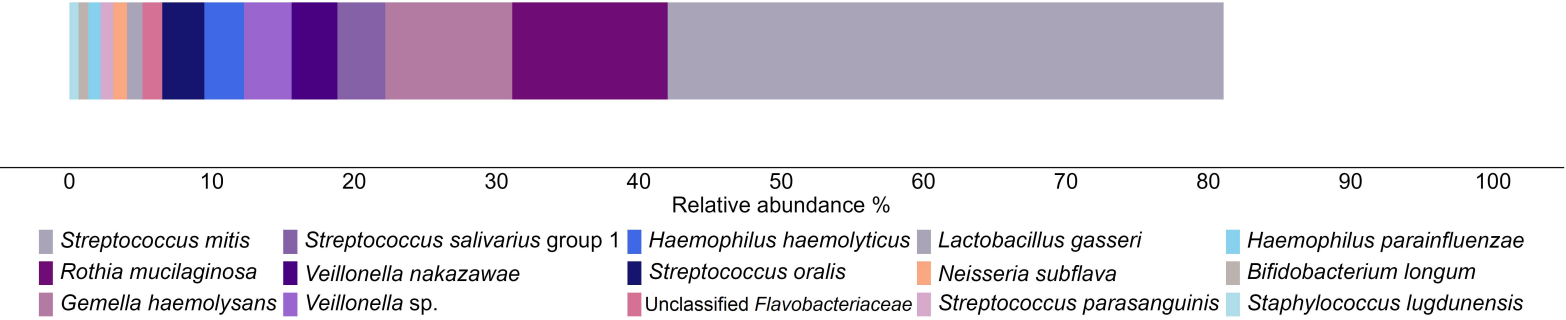
The composition of the oral microbiome in breastfed infants at 3 months of age. OTUs with a mean relative abundance of > 0.5% are shown.

All infants in this sub-study were born at term, had no major health complications, and were exclusively breastfed (**Table 1**), with an average daily human milk intake of 766.9 mL. Baseline maternal and infant characteristics were compared between dyads with and without available milk intake data. No significant differences were observed between the groups (all P > 0.05), indicating that the subset with intake data is broadly representative of the complete cohort. The infant oral microbiome at 3 months of age was strongly dominated by *Streptococcus mitis* (**Figure 1**). Principal coordinates analysis (PCoA) of Aitchison distances from CLR-transformed OTU abundances showed that samples clustered closely around the origin, with no clear separation across the cohort (**Supplementary Figure 1**).

**Table 1:** Participant characteristics.

Characteristic	Concentration analysis (n=45) Mean ± SD or n (%)	Intake analysis (n=42) Mean ± SD or n (%)
Maternal age at delivery (years)	32.5 ± 5.1	32.6 ± 5.1
Maternal ethnicity		
Caucasian	37 (82.2%)	36 (85.7%)
Other	8 (17.8%)	6 (14.3%)
Delivery mode		
Vaginal	29 (64.4%)	29 (69.0%)
Planned caesarean	9 (20.0%)	7 (16.7%)
Emergency caesarean	7 (15.5%)	6 (14.3%)
Intrapartum antibiotic exposure		
Yes	23 (51.1%)	20 (47.6%)
Maternal pre-pregnancy BMI (kg/m^2^)	24.7 ± 5.9	24.5 ± 5.0
Underweight	2 (4.4%)	1 (2.4%)
Normal	21 (46.7%)	20 (47.6%)
High	8 (17.8%)	7 (16.7%)
Obese	5 (11.1%)	4 (9.5%)
Missing	9 (20.0%)	10 (23.8%)
Infant sex		
Female	26 (57.8%)	24 (57.1%)
Feeding mode		
Exclusive breastfeeding	45 (100%)	42 (100%)
Pacifier use within the first week	11 (24.4%)	11 (26.2%)
Age of first solid food intake (weeks)	23.0 ± 4.3	22.3 ± 3.5

BMI, body mass index; SD, standard deviation; n, number of participants.

### 3.1 Associations of human milk component concentrations with infant oral microbiome

**Figure 2:**
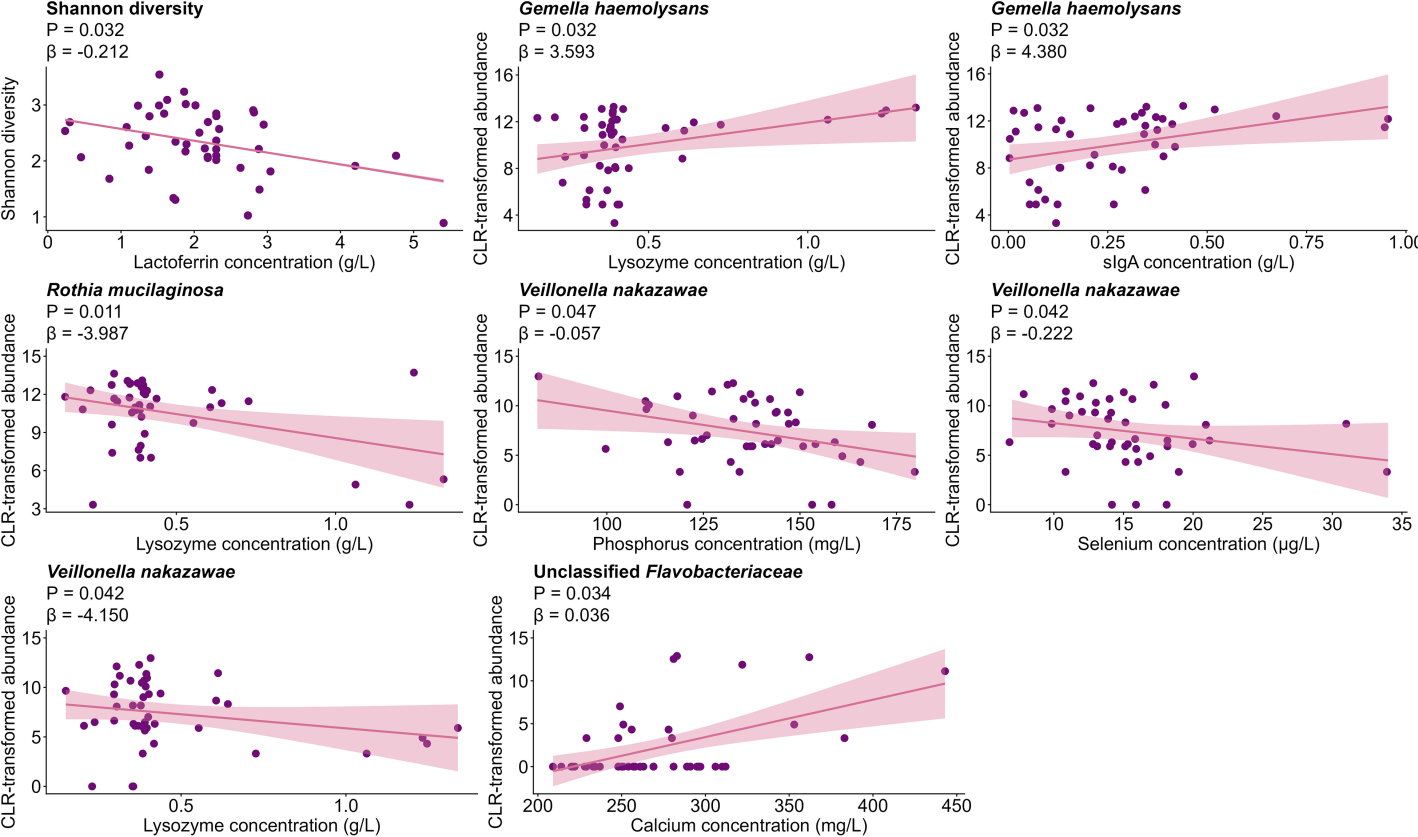
Associations between human milk component concentrations and infant oral microbiome. Lines represent linear model fits, and shaded areas show 95% confidence intervals.

We first assessed concentrations of milk components (**Supplementary Table 7**). Concentration of lactoferrin was negatively associated with infant oral Shannon diversity (ß = -0.212 [–0.374, –0.049], P = 0.032). Lysozyme concentration was positively associated with *Gemella haemolysans* (ß = 3.593 [0.779, 6.406], P = 0.032) and negatively associated with *Rothia mucilaginosa* (ß = –3.987 [–6.642, –1.333], P = 0.011) and *Veillonella nakazawae* (ß = –4.150 [–7.511, –0.789], P = 0.042). sIgA concentration was positively associated with *G. haemolysans* (ß = 4.380 [0.942, 7.819], P = 0.032). Infants who consumed milk with higher phosphorus and selenium concentrations harboured lower abundances of *V. nakazawae* (phosphorus: ß = –0.057 [–0.104, –0.009], P = 0.047; selenium: ß = –0.222 [–0.402, –0.042], P = 0.042), while infants who consumed milk with higher calcium concentrations harboured higher abundances of an OTU that mapped to an unknown *Flavobacteriaceae* (ß = 0.036 [0.014, 0.059], P = 0.034) (**Figure 2**).

### 3.2 Associations of human milk component estimated intakes with infant oral microbiome

**Figure 3:**
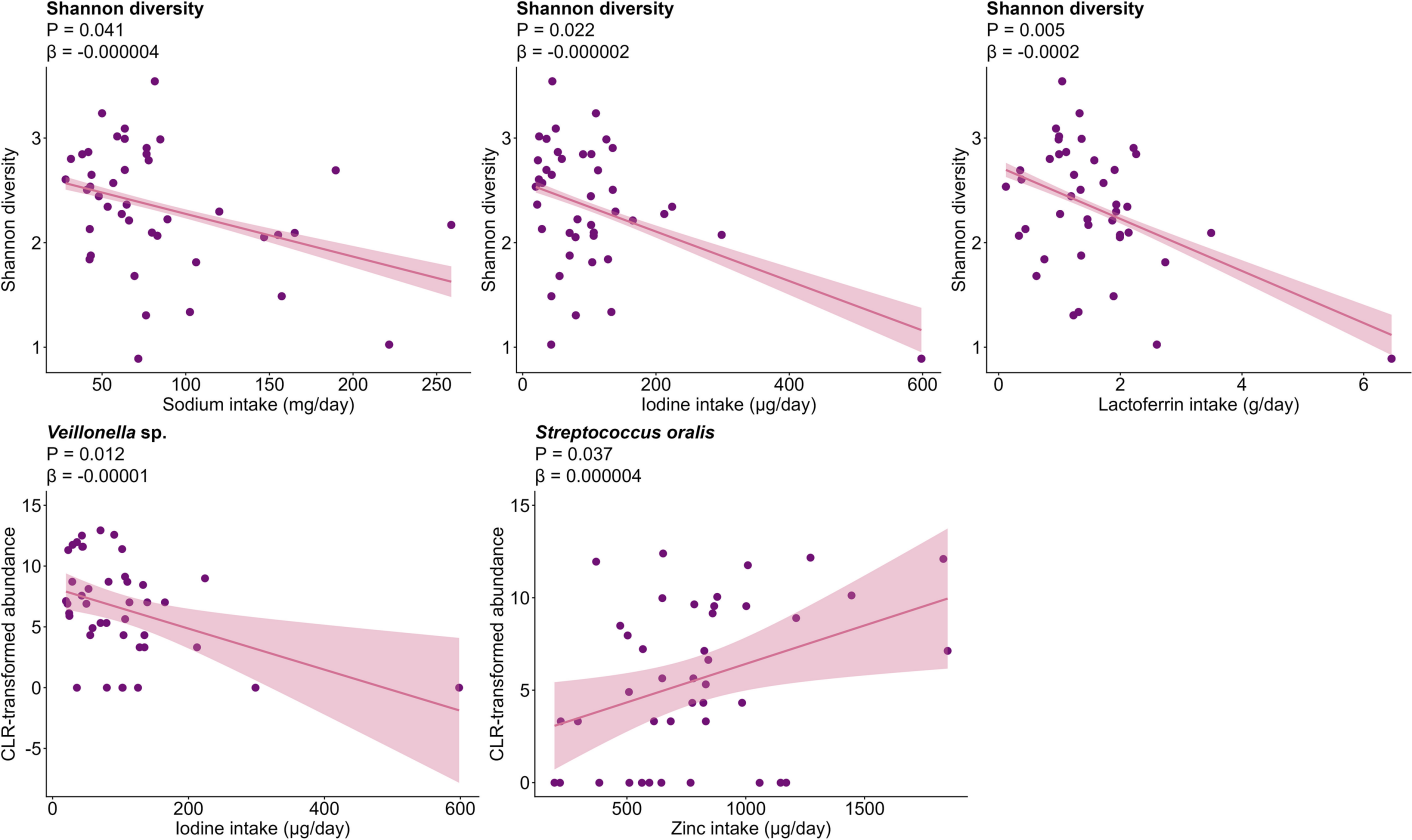
Associations between human milk component daily intakes and infant oral microbiome. Lines represent linear model fits, and shaded areas show 95% confidence intervals.

While many of the associations observed for concentrations were mirrored in the intake models, the effect sizes were more modest (**Supplementary Table 8**). In line with the concentration findings, we observed a negative association between estimated intake of lactoferrin and Shannon diversity (ß = -0.0002 [–0.0004, –0.0001], P = 0.005), with additional negative associations for sodium and iodine estimated intakes (sodium: ß = –0.000004 [–0.000007, –0.0000008], P = 0.041; iodine: ß = –0.000002 [–0.000004, –0.0000006], P = 0.022). A negative association was also detected between estimated iodine intake and *Veillonella* sp. (ß = –0.00001 [–0.00002, –0.000005], P = 0.012). In contrast, zinc estimated intake was positively associated with *Streptococcus oralis*, the dominant OTU in this cohort (ß = 0.000004 [0.000001, 0.000007], P = 0.037) (**Figure 3**).

## 4 Discussion

While human milk components are known to influence bacterial communities in the gut (Gotteland and Magne, 2017; Xi et al., 2023; Flores Ventura et al., 2024; Kebbe et al., 2024), their effect within the breastfed infant oral cavity is not clear. Here, we report significant relationships of human milk components with microbial composition among exclusively breastfed infants.AMPs showed the strongest effect size on oral microbial profiles in terms of concentrations. Human milk AMPs play key roles in shaping early host-microbe interactions, contributing to mucosal immunity, pathogen defence, and the modulation of microbial communities (Doare et al., 2018; Carr et al., 2021). We found both concentrations and estimated intakes of milk AMPs were associated with reduced Shannon diversity, higher abundance of *Gemella*, and reduced abundance of *Veillonella* and *Rothia*. The negative association between lactoferrin and Shannon diversity may be due to its ability to sequester iron and thereby restrict the growth of iron dependent bacteria (Jenssen and Hancock, 2009). This effect was consistently observed in both the concentration and intake analyses, suggesting similar local and systemic effects of its broad antimicrobial properties. However, in a previous clinical trial of low-iron infant formula supplemented with bovine lactoferrin (at concentrations 1000 times greater than in human milk), the addition of lactoferrin did not significantly alter infant oral alpha diversity (Anticona et al., 2025). This may suggest that isolating a single component from the human milk matrix to modulate infant colonisation is not an effective strategy. Human milk feeding has repeatedly been associated with lower infant oral Shannon diversity (Butler et al., 2022; Anticona et al., 2025), and the results here suggest that this may in part be explained by lactoferrin.

Similarly, we observed that both lower concentration of lysozyme were associated with increased relative abundances of *V. nakazawae* and *R. mucilaginosa* consistent with lysozyme’s antimicrobial activity, which can disrupt bacterial cell walls and inhibit the growth of susceptible taxa (Doare et al., 2018; Carr et al., 2021). Lysozyme exerts bactericidal effects by cleaving peptidoglycan in bacterial cell walls, primarily targeting Gram-positive taxa (Lee et al., 2004). This raises two questions: why was there a negative association between a Gram-negative bacteria and lysozyme, and why was there a negative association between one Gram-positive bacteria and lysozyme, but not other highly abundant Gram-positives such as *Streptococcus*? Interestingly, *Veillonella* has atypical lipopolysaccharide, and a very thin peptidoglycan layer. Thus, if other human milk or salivary components, such as lactoferrin, compromise the outer membrane, *Veillonella*’s peptidoglycan layer may be vulnerable to lysozyme. As for the lack of association with the dominant Gram-positive taxa in the oral cavity, *Streptococcus* species often express protective peptidoglycan modifications, such as O-acetylation or N-deacetylation, which make them less vulnerable to enzymatic cleavage (Vollmer, 2008). These protective mechanisms are well described in several oral streptococci, but similar modifications have not been shown in *Veillonella* or *Rothia*. *Rothia* typically form small clusters in early oral biofilms, and detailed information on their biofilm structure, thickness, or matrix is still lacking (Palmer et al., 2017). *Streptococcus*, on the other hand, forms dense, extracellular polymeric substance-rich biofilms, which may further protect them from lysozyme.

The positive association between *Gemella* species and sIgA concentration may reflect a role for sIgA in promoting colonisation and immune tolerance of commensal oral bacteria. sIgA is known to reduce bacterial adherence to mucosal surfaces and, in the oral environment, can interact with mucins to form complexes that facilitate bacterial aggregation and clearance (Marcotte and Lavoie, 1998; Gibbins et al., 2015). While sIgA also influences colonisation of other early oral taxa, such as *Veillonella* (Zhou et al., 2021; Singh et al., 2023), direct evidence linking milk-derived sIgA concentration to specific taxa remains limited. Nevertheless, these findings highlight a potential role for human milk in supporting oral immune function through sIgA-dependent regulation of key commensal bacteria, contributing to the establishment and modulation of early oral microbial communities.

Minerals and trace elements are vital for infant growth and development, contributing to hormone synthesis, immune system function, and serving as cofactors in antioxidant defence mechanisms (Black et al., 2008; Choi et al., 2016; Perrella et al., 2021). We found that concentrations and estimated intakes of several minerals were significantly associated with the relative abundances of specific oral bacterial taxa. We observed that higher estimated daily intakes of the antimicrobial minerals sodium and iodine were associated with reduced infant oral Shannon diversity, with increased iodine estimated intake also linked to lower relative abundance of *Veillonella* sp. These findings are biologically plausible. *In vitro* studies have shown that molecular iodine mouth rinses markedly reduce viable counts of subgingival biofilm bacteria, including periodontal pathogens (Rams et al., 2022). Clinically, short-term application of povidone-iodine has been shown to modestly reduce Shannon diversity in dental plaque (Reilly et al., 2016). Elsewhere in the gut, higher dietary sodium intake has been linked to reduced microbiome diversity (Ferguson et al., 2019). Collectively, these data provide support for our observations, suggesting that higher sodium and iodine estimated intakes via human milk may constrain early oral microbial diversity.

An interesting finding of this study was the positive association between estimated intake of zinc and *S. oralis.* Supporting evidence from the gut microbiome shows that, in a cohort of 77 mother–infant dyads, higher milk zinc concentrations were correlated with increased infant gut *Bacteroides* abundance (Flores Ventura et al., 2024). Within the oral cavity, evidence from a recent randomized controlled study suggests that brushing with a zinc-based toothpaste alters the composition of the plaque microbiome, with small increases in several *Streptococcus* species, as well as changes in their metatranscriptome, with specific reduction in glycolysis and increases in processes linked to gum health and systemic health (Adams et al., 2025). Studies have demonstrated that zinc exhibits antimicrobial activity, inhibiting pathogenic species linked to dental caries and periodontitis, such as *S. mutans* and *Porphyromonas gingivalis* (Lynch, 2011; Griauzdyte and Jagelaviciene, 2023), This may suggest that early zinc exposure can influence microbial colonisation across mucosal sites.

In our study, both selenium and phosphorus concentrations were negatively associated with *V. nakazawae.* Selenium, which acts as a cofactor for redox enzymes thereby modulating oxidative stress (Maia et al., 2023), can be increased in milk via maternal dietary supplementation, providing infants with biologically relevant exposure (Trafikowska et al., 1998). Evidence from both murine and human studies have linked dietary selenium estimated intake to gut microbiome profiles (Kasaikina et al., 2011; Zhang et al., 2021); however, this is the first report of such effects within the infant oral microbiome. There is nevertheless evidence for a role of this mineral in the oral cavity, with dental sealants containing selenium shown to reduce pathogenic biofilm formation (Tran et al., 2013).

We observed a positive association between calcium concentrations and an unknown *Flavobacteriaceae*, indicating that calcium may selectively promote the growth of specific commensal taxa in the infant oral cavity. *In vitro* saliva-derived biofilm models have shown that calcium supplementation increases biofilm biomass and alters community composition, including the relative abundance of genera such as *Veillonella* (Shokeen et al., 2022). Calcium can bind to extracellular polymeric substances, enhancing biofilm stability and influencing microbial colonisation dynamics (Rovera et al., 2025). Although some *Flavobacteriaceae* can form biofilms, little is known about biofilm formation by oral *Flavobacteriaceae*.

A major strength of this study was the consideration of both concentrations and estimated intakes of human milk components. During breastfeeding, the infant’s oral cavity is regularly exposed to human milk. Therefore, the concentration that each component reaches is expected to have immediate, local effects on the infant oral microbiome. Conversely, cumulative estimated intakes are expected to exert systemic effects. As milk composition and intake differ across mother-infant pairs, infants consuming human milk with the same concentration of a component may receive different doses. Our analysis identified largely consistent associations for both concentrations and estimated intakes; however, the effect sizes for estimated intakes were approximately 1,000 times weaker than those for concentrations. Two hypotheses may explain this lesser effect of estimated intakes. There may be a biological explanation, whereby direct local effects are more important than indirect systemic effects for the oral microbiome. Alternatively, the explanation may be a mathematical artefact. Intake is calculated by multiplying concentration by milk volume consumed, so a strong effect with concentration may show up in estimated intake data, even though this is predominately driven by concentration. Regardless of the explanation, our data suggest that oral concentration of milk components influences microbial ecology.

*Post-hoc* power analyses indicated that statistical power varied considerably across models, reflecting differences in marginal effect sizes relative to the sample size. Power was adequate for the association between lactoferrin and Shannon diversity (power = 0.86 and 0.83 for concentration and intake models respectively), suggesting this is the most robust finding of the study. For the majority of OTU-level associations, marginal effect sizes were small and power was low (range: 0.05–0.86), indicating that true associations of small-to-moderate magnitude may have gone undetected. These findings should therefore be interpreted as exploratory and hypothesis-generating, and replication in larger, adequately powered cohorts is warranted. This exploratory analysis did not include formal sensitivity analyses. Instead, CLR transformation and Benjamini–Hochberg correction were pre-specified analytical choices appropriate for compositional microbiome data and control of false discovery rate, respectively. Covariates were selected based on prior findings from this cohort. Larger, independent cohorts are needed to confirm these findings and evaluate robustness across alternative analytical specifications. Nevertheless, exclusive breastfeeding, human milk estimated intake data, and long amplicon sequencing are major strengths. The findings presented here should be replicated in larger cohorts and tested in *in vitro* studies to comprehensively elucidate the influence of human milk composition on the infant oral microbiome. Another limitation of this study is that daily intakes of human milk component concentrations were estimated from a single milk sample. There is some evidence that milk composition varies across the day. While lactose concentrations are relatively stable, there is some, inconsistent, evidence that antimicrobial proteins and micronutrients may vary over the course of the day, with the strongest evidence for time-of-day variation in iron (Cacho and Lawrence, 2017; Dror and Allen, 2018; Italianer et al., 2020; Bilston-John et al., 2021b). As such, we accounted for time-of-day in our iron models. However, short-term time-of-day variation is generally modest compared with inter-individual differences, and the use of a single sample to estimate typical exposure is consistent with many observational studies. Future studies incorporating repeated milk sampling across the day would allow more precise estimation of infant exposure.

In summary, the findings of this study suggest that concentrations of human milk components and, to a lesser extent, daily infant estimated intakes, may influence the infant oral microbiome, with potential implications for oral health.

## Data Availability

The datasets presented in this study can be found in online repositories. The names of the repository/repositories and accession number(s) can be found below: https://www.ncbi.nlm.nih.gov/, BioProject accession: PRJNA1186108.
