# Supplementary material for: Concentrations of selected human milk components influence the infant oral microbiome to a greater degree than estimated intakes
Source: Front Cell Infect Microbiol. 2026 May 28;16:1765736. doi: 10.3389/fcimb.2026.1765736 (PMC13254919; doi:10.3389/fcimb.2026.1765736)
Supplement: Supplementary file 1 [file Table1.docx]

**Supplementary tables**

**Supplementary Table S1**. Assay QC details for analysis of human milk macronutrients, hormones, and antimicrobial proteins.

| **Category** | **Component** | **N** | **Detection limit** | **Recovery ± S.D. (%)** | **The coefficient of variation** | |
| --- | --- | --- | --- | --- | --- | --- |
|  |  |  |  |  | **Intra-assay** | **Inter-assay** |
| **Macronutrients** | Lactose | 6 | 0.11 g/L | 100% ± 3% | 4.1% | 7.1% |
| **Antimicrobial proteins** | Lysozyme | 5 | 0.006 μg/mL | 99% ± 3.8% | 0.78% | 0.19%. |
|  | Lactoferrin | 7 | 0.008 µg/ mL | 98% ± 5.3% | 0.66% | 1.53%. |
|  | sIgA | 7 | < 0.0012 µg/ mL | 100% ± 3.9% | 1.2% | 12% |

N: the number of replicate measurements used to calculate detection limits and recovery.

**Supplementary Table S2.**  The recovery of minerals in human milk using ICP-OES

| **Elements** | **Wavelength (nm)** | **N** | **Recovery ± S.D. (%)** |
| --- | --- | --- | --- |
| Calcium | 315.887 | 5 | 98±3 |
|  | 317.933 | 5 | 96±3 |
| Copper | 324.75 | 5 | 97±2 |
|  | 327.395 | 5 | 99±2 |
| Iron | 238.204 | 5 | 98±6 |
|  | 259.940 | 5 | 98±6 |
| Potassium | 766.491 | 5 | 104±4 |
|  | 769.897 | 5 | 105±4 |
| Magnesium | 280.270 | 5 | 92±9 |
|  | 285.213 | 5 | 94±9 |
| Sodium | 368.821 | 5 | 101±4 |
| Phosphorus | 213.618 | 5 | 107±3 |
|  | 214.914 | 5 | 106±4 |
| Zinc | 202.548 | 5 | 97 ± 2 |
|  | 213.857 | 5 | 98 ± 3 |

**Supplementary Table S3**. Taxonomic assignments of OTUs based on SILVA and BLAST analyses.

| **OTU** | **SILVA genus assignment** | **BLAST species assignment** | **Sequnce ID** | **Identity accuracy** | **Coverage** |
| --- | --- | --- | --- | --- | --- |
| Otu000001 | *Streptococcus* | *Streptococcus mitis* | CP067992.1 | 98.58% | 100% |
| Otu000002 | *Gemella* | *Gemella haemolysans* | CP083637.1 | 99.53% | 100% |
| Otu000003 | *Rothia* | *Rothia mucilaginosa* | CP023510.1 | 99.66% | 100% |
| Otu000004 | *Streptococcus* | *Streptococcus salivarius* group 1 | CP015283.1 | 97.63% | 100% |
| Otu000005 | *Veillonella* | *Veillonella* sp. | AY923122.1 | 97.86% | 100% |
| Otu000006 | *Neisseria* | *Neisseria subflava* | MF480369.1 | 99.52% | 99% |
| Otu000007 | *Streptococcus* | *Streptococcus oralis* | CP034442.1 | 97.84% | 100% |
| Otu000008 | *Haemophilus* | *Haemophilus haemolyticus* | CP031240.1 | 98.91% | 100% |
| Otu000009 | *Veillonella* | *Veillonella nakazawae* | AP022321.1 | 99.60% | 100% |
| Otu000010 | *Staphylococcus* | *Staphylococcus lugdunensis* | CP014023.2 | 100% | 100% |
| Otu000011 | *Haemophilus* | *Haemophilus parainfluenzae* | CP133470.1 | 98.51% | 100% |
| Otu000012 | Unclassified *Flavobacteriaceae* | Unclassified *Flavobacteriaceae* | EF419381.1 | 99.86% | 99% |
| Otu000013 | *Streptococcus* | *Streptococcus parasanguinis* | CP134147.1 | 99.59% | 100% |
| Otu000015 | *Bifidobacterium* | *Bifidobacterium longum* | CP102541.1 | 99.86% | 100% |
| Otu000016 | *Lactobacillus* | *Lactobacillus gasseri* | CP054875.1 | 98.52% | 99% |

**Supplementary Table S4**. Genera detected in negative extraction controls (EC, n = 8) and negative PCR controls (NTC, n = 8). Data are read numbers.

| Genus | **EC1** | **EC2** | **EC3** | **EC4** | **EC5** | **EC6** | **EC7** | **EC8** | **NTC1** | **NTC2** | **NTC3** | **NTC4** | **NTC5** | **NTC6** | **NTC7** | **NTC8** |
| --- | --- | --- | --- | --- | --- | --- | --- | --- | --- | --- | --- | --- | --- | --- | --- | --- |
| *Actinomyces* | 2 | 2 | 0 | 0 | 3 | 1 | 1 | 0 | 1 | 1 | 0 | 0 | 1 | 1 | 0 | 0 |
| *Atopobium* | 2 | 1 | 0 | 1 | 0 | 0 | 0 | 0 | 0 | 1 | 2 | 0 | 0 | 0 | 0 | 0 |
| *Bergeyella* | 7 | 1 | 5 | 0 | 1 | 2 | 1 | 1 | 0 | 1 | 8 | 1 | 2 | 1 | 4 | 0 |
| *Gemella* | 37 | 0 | 1 | 0 | 0 | 0 | 1 | 1 | 1 | 0 | 1 | 2 | 2 | 0 | 3 | 1 |
| *Granulicatella* | 1 | 3 | 0 | 0 | 2 | 2 | 1 | 5 | 1 | 1 | 1 | 1 | 2 | 2 | 1 | 2 |
| *Haemophilus* | 3 | 0 | 0 | 0 | 0 | 0 | 0 | 0 | 0 | 0 | 0 | 0 | 0 | 0 | 0 | 0 |
| *Lactobacillus* | 1 | 1 | 1 | 0 | 1 | 1 | 0 | 0 | 0 | 1 | 3 | 0 | 1 | 1 | 0 | 1 |
| *Neisseria* | 8 | 2 | 5 | 2 | 5 | 4 | 1 | 2 | 0 | 4 | 2 | 1 | 9 | 2 | 2 | 3 |
| *Porphyromonas* | 9 | 0 | 0 | 0 | 0 | 0 | 0 | 0 | 0 | 1 | 1 | 0 | 2 | 1 | 0 | 0 |
| *Rothia* | 70 | 3 | 2 | 1 | 1 | 4 | 1 | 1 | 3 | 1 | 2 | 0 | 3 | 1 | 2 | 3 |
| Unclassified *Saccharimonadales* | 4 | 4 | 8 | 1 | 11 | 7 | 5 | 26 | 3 | 10 | 10 | 6 | 7 | 7 | 11 | 1 |
| *Sebaldella* | 1 | 0 | 0 | 0 | 0 | 1 | 0 | 0 | 0 | 0 | 0 | 0 | 0 | 0 | 0 | 0 |
| *Staphylococcus* | 3 | 0 | 3 | 0 | 6 | 5 | 1 | 0 | 2 | 4 | 1 | 2 | 3 | 2 | 5 | 0 |

| Genus | **EC1** | **EC2** | **EC3** | **EC4** | **EC5** | **EC6** | **EC7** | **EC8** | **NTC1** | **NTC2** | **NTC3** | **NTC4** | **NTC5** | **NTC6** | **NTC7** | **NTC8** |  |
| --- | --- | --- | --- | --- | --- | --- | --- | --- | --- | --- | --- | --- | --- | --- | --- | --- | --- |
| *Streptococcus* | 719 | 13 | 18 | 7 | 27 | 16 | 11 | 60 | 6 | 11 | 21 | 16 | 21 | 15 | 13 | 4 |  |
| Unclassified *Actinobacteria* | 2 | 2 | 0 | 0 | 0 | 3 | 1 | 0 | 0 | 0 | 3 | 1 | 1 | 1 | 1 | 0 |  |
| Unclassified *Bacilli* | 3 | 1 | 1 | 0 | 2 | 3 | 2 | 0 | 0 | 0 | 2 | 0 | 1 | 2 | 0 | 1 |  |
| Unclassified bacteria | 37 | 2 | 1 | 0 | 2 | 5 | 3 | 0 | 1 | 6 | 4 | 2 | 6 | 1 | 2 | 1 |  |
| Unclassified *Carnobacteriaceae* | 3 | 1 | 2 | 1 | 2 | 8 | 1 | 0 | 4 | 4 | 7 | 1 | 4 | 5 | 3 | 2 |  |
| Unclassified *Lactobacillales* | 7 | 4 | 2 | 1 | 12 | 18 | 6 | 0 | 0 | 1 | 3 | 4 | 9 | 8 | 11 | 3 |  |
| Unclassified *Leptotrichiaceae* | 1 | 1 | 0 | 0 | 3 | 0 | 0 | 3 | 1 | 0 | 2 | 0 | 2 | 0 | 1 | 0 |  |
| Unclassified *Micrococcaceae* | 6 | 0 | 0 | 0 | 0 | 0 | 0 | 0 | 0 | 0 | 2 | 0 | 0 | 0 | 0 | 0 |  |
| Unclassified *Micrococcales* | 25 | 5 | 6 | 2 | 2 | 9 | 2 | 4 | 3 | 4 | 8 | 6 | 8 | 4 | 4 | 2 |  |
| Unclassified *Neisseriaceae* | 1 | 0 | 4 | 2 | 3 | 4 | 61 | 9 | 0 | 0 | 0 | 0 | 0 | 1 | 1 | 2 |  |
| Unclassified *Porphyromonadaceae* | 1 | 3 | 1 | 0 | 4 | 1 | 3 | 1 | 1 | 2 | 0 | 1 | 7 | 2 | 4 | 0 |  |
| Unclassified *Streptococcaceae* | 33 | 1 | 4 | 0 | 2 | 0 | 0 | 0 | 0 | 1 | 1 | 0 | 0 | 0 | 2 | 0 |  |
| *Veillonella* | 549 | 8 | 9 | 2 | 6 | 7 | 6 | 18 | 3 | 8 | 4 | 11 | 8 | 8 | 5 | 2 |  |
| Others* | 1 | 7 | 6 | 9 | 51 | 57 | 95 | 33 | 6 | 13 | 4 | 9 | 41 | 47 | 27 | 15 |  |

*“Others” represents genera with ≤ 26 reads in negative control

**Supplementary Table S5**. Outputs from PERMANOVA assessing the beta diversity of the oral microbiome in relation to human milk components concentrations

| **Component** | **R²** | **P-value** | **BH corrected P-value** |
| --- | --- | --- | --- |
| Ca | 0.0279 | 0.262 | 0.681 |
| Na | 0.0272 | 0.284 | 0.681 |
| K | 0.0238 | 0.401 | 0.681 |
| P | 0.0259 | 0.350 | 0.681 |
| Mg | 0.0285 | 0.223 | 0.681 |
| I | 0.0230 | 0.427 | 0.681 |
| Se | 0.0118 | 0.881 | 0.939 |
| Cu | 0.0176 | 0.659 | 0.811 |
| Mn | 0.0210 | 0.462 | 0.681 |
| Mo | 0.0151 | 0.782 | 0.893 |
| Fe | 0.0383 | 0.095 | 0.506 |
| Zn | 0.0111 | 0.940 | 0.940 |
| Lactose | 0.0208 | 0.500 | 0.681 |
| Lactoferrin | 0.0208 | 0.511 | 0.681 |
| Lysozyme | 0.0431 | 0.069 | 0.506 |
| sIgA | 0.0409 | 0.063 | 0.506 |

Ca, calcium; Na, sodium; K, potassium; P, phosphorus; Mg, magnesium; I, iodine; Se, selenium; Cu, copper; Mn, manganese; Mo, molybdenum; Fe, iron; Zn, zinc; sIgA, secretory immunoglobulin A.

**Supplementary Table S6**. Outputs from PERMANOVA assessing the beta diversity of the oral microbiome in relation to human milk components intakes

| **Component** | **R²** | **P-value** | **BH corrected P-value** |
| --- | --- | --- | --- |
| Ca | 0.0352 | 0.131 | 0.392 |
| Na | 0.0334 | 0.195 | 0.392 |
| K | 0.0319 | 0.181 | 0.392 |
| P | 0.0329 | 0.161 | 0.392 |
| Mg | 0.0193 | 0.643 | 0.740 |
| I | 0.0331 | 0.196 | 0.392 |
| Se | 0.0151 | 0.812 | 0.843 |
| Cu | 0.0254 | 0.352 | 0.500 |
| Mn | 0.0303 | 0.243 | 0.441 |
| Mo | 0.0147 | 0.843 | 0.843 |
| Fe | 0.0421 | 0.071 | 0.392 |
| Zn | 0.0335 | 0.155 | 0.392 |
| Lactose | 0.0305 | 0.193 | 0.392 |
| Lactoferrin | 0.0367 | 0.144 | 0.392 |
| Lysozyme | 0.0259 | 0.375 | 0.500 |
| sIgA | 0.0362 | 0.107 | 0.392 |

Ca, calcium; Na, sodium; K, potassium; P, phosphorus; Mg, magnesium; I, iodine; Se, selenium; Cu, copper; Mn, manganese; Mo, molybdenum; Fe, iron; Zn, zinc; sIgA, secretory immunoglobulin A.

**Supplementary Table S7**. Outputs from covariate-adjusted linear regression models assessing the associations between human milk component concentrations and the infant oral microbiome at 3 months postpartum.

| **Response variable** | **Explanatory variables** | **Estimate** | **Standard Error** | **P-value** | **BH corrected P-value** | **Lower CI** | **Upper CI** |
| --- | --- | --- | --- | --- | --- | --- | --- |
| Shannon diversity | Ca | -0.001 | 0.002 | 0.699 | 0.869 | -0.005 | 0.003 |
| Shannon diversity | Na | -0.003 | 0.002 | 0.068 | 0.135 | -0.006 | 0.000 |
| Shannon diversity | K | -0.001 | 0.001 | 0.568 | 0.776 | -0.004 | 0.002 |
| Shannon diversity | P | 0.001 | 0.005 | 0.838 | 0.896 | -0.008 | 0.010 |
| Shannon diversity | Mg | 0.012 | 0.012 | 0.325 | 0.516 | -0.012 | 0.037 |
| Shannon diversity | I | -0.002 | 0.001 | 0.042 | 0.089 | -0.004 | 0.000 |
| Shannon diversity | Se | 0.006 | 0.018 | 0.747 | 0.873 | -0.030 | 0.041 |
| Shannon diversity | Cu | 0.000 | 0.001 | 0.829 | 0.896 | -0.003 | 0.003 |
| Shannon diversity | Mn | -0.077 | 0.083 | 0.360 | 0.552 | -0.239 | 0.086 |
| Shannon diversity | Mo | -0.011 | 0.040 | 0.778 | 0.873 | -0.091 | 0.068 |
| Shannon diversity | Fe | -0.001 | 0.001 | 0.176 | 0.311 | -0.002 | 0.000 |
| Shannon diversity | Time of milk sampling: Afternoon | 0.000 | 0.216 | 1.000 | 1.000 | -0.422 | 0.423 |
| Shannon diversity | Time of milk sampling: Night | -0.066 | 0.228 | 0.774 | 0.873 | -0.514 | 0.382 |
| Shannon diversity | Zn | 0.000 | 0.000 | 0.976 | 0.997 | 0.000 | 0.000 |
| Shannon diversity | Lactose | -0.003 | 0.007 | 0.729 | 0.873 | -0.017 | 0.012 |
| Shannon diversity | Lactoferrin | -0.212 | 0.083 | 0.014 | **0.032** | -0.374 | -0.049 |
| Shannon diversity | Lysozyme | 0.041 | 0.338 | 0.905 | 0.946 | -0.622 | 0.703 |
| Shannon diversity | sIgA | 0.703 | 0.399 | 0.085 | 0.164 | -0.079 | 1.485 |
| Shannon diversity | Delivery mode: emergency caesarean | -0.330 | 0.247 | 0.188 | 0.320 | -0.813 | 0.153 |
| Shannon diversity | Delivery mode: planned caesarean | -0.200 | 0.223 | 0.376 | 0.558 | -0.638 | 0.238 |
| Shannon diversity | First week pacifier: yes | 0.300 | 0.205 | 0.150 | 0.277 | -0.101 | 0.702 |
| Shannon diversity | Siblings: yes | -0.234 | 0.203 | 0.254 | 0.417 | -0.632 | 0.163 |
| Shannon diversity | Pre-pregnancy BMI: high and obese | 0.091 | 0.207 | 0.662 | 0.846 | -0.314 | 0.496 |
| Richness | Ca | 0.080 | 0.366 | 0.828 | 0.892 | -0.638 | 0.798 |
| Richness | Na | -0.586 | 0.296 | 0.054 | 0.114 | -1.166 | -0.005 |
| Richness | K | -0.066 | 0.266 | 0.805 | 0.892 | -0.587 | 0.455 |
| Richness | P | 0.601 | 0.906 | 0.511 | 0.671 | -1.175 | 2.376 |
| Richness | Mg | 3.195 | 2.363 | 0.183 | 0.291 | -1.437 | 7.827 |
| Richness | I | -0.381 | 0.192 | 0.053 | 0.114 | -0.757 | -0.005 |
| Richness | Se | 0.730 | 3.458 | 0.834 | 0.892 | -6.048 | 7.507 |
| Richness | Cu | 0.074 | 0.279 | 0.793 | 0.892 | -0.474 | 0.621 |
| Richness | Mn | -19.057 | 15.702 | 0.232 | 0.350 | -49.833 | 11.720 |
| Richness | Mo | -2.103 | 7.719 | 0.787 | 0.892 | -17.232 | 13.026 |
| Richness | Fe | -0.214 | 0.115 | 0.070 | 0.140 | -0.440 | 0.011 |
| Richness | Time of milk sampling: Afternoon | -4.454 | 39.166 | 0.910 | 0.951 | -81.220 | 72.311 |
| Richness | Time of milk sampling: Night | -30.046 | 41.511 | 0.473 | 0.640 | -111.408 | 51.316 |
| Richness | Zn | -0.001 | 0.040 | 0.983 | 0.983 | -0.078 | 0.077 |
| Richness | Lactose | -0.525 | 1.425 | 0.714 | 0.865 | -3.318 | 2.268 |
| Richness | Lactoferrin | -24.861 | 16.562 | 0.141 | 0.251 | -57.322 | 7.600 |
| Richness | Lysozyme | 31.978 | 64.382 | 0.622 | 0.773 | -94.210 | 158.166 |
| Richness | sIgA | 93.327 | 77.595 | 0.236 | 0.350 | -58.760 | 245.414 |
| Richness | Delivery mode: emergency caesarean | -63.032 | 46.444 | 0.182 | 0.291 | -154.063 | 27.998 |
| Richness | Delivery mode: planned caesarean | -62.988 | 42.082 | 0.142 | 0.251 | -145.469 | 19.494 |
| Richness | First week pacifier: yes | 55.028 | 39.108 | 0.167 | 0.284 | -21.623 | 131.679 |
| Richness | Siblings: yes | -33.593 | 38.959 | 0.393 | 0.548 | -109.952 | 42.766 |
| Richness | Pre-pregnancy BMI: high and obese | 23.766 | 39.571 | 0.551 | 0.704 | -53.793 | 101.324 |
| *Streptococcus mitis* | Ca | 0.008 | 0.005 | 0.133 | 0.255 | -0.002 | 0.018 |
| *Streptococcus mitis* | Na | 0.005 | 0.004 | 0.268 | 0.441 | -0.004 | 0.014 |
| *Streptococcus mitis* | K | 0.003 | 0.004 | 0.460 | 0.572 | -0.005 | 0.011 |
| *Streptococcus mitis* | P | 0.009 | 0.013 | 0.520 | 0.629 | -0.018 | 0.035 |
| *Streptococcus mitis* | Mg | 0.006 | 0.036 | 0.866 | 0.905 | -0.064 | 0.076 |
| *Streptococcus mitis* | I | 0.004 | 0.003 | 0.221 | 0.389 | -0.002 | 0.009 |
| *Streptococcus mitis* | Se | 0.004 | 0.051 | 0.943 | 0.964 | -0.096 | 0.104 |
| *Streptococcus mitis* | Cu | -0.004 | 0.004 | 0.296 | 0.460 | -0.012 | 0.004 |
| *Streptococcus mitis* | Mn | 0.001 | 0.236 | 0.997 | 0.997 | -0.461 | 0.463 |
| *Streptococcus mitis* | Mo | 0.111 | 0.113 | 0.331 | 0.491 | -0.110 | 0.332 |
| *Streptococcus mitis* | Fe | 0.003 | 0.002 | 0.073 | 0.152 | 0.000 | 0.006 |
| *Streptococcus mitis* | Time of milk sampling: Afternoon | -0.865 | 0.576 | 0.141 | 0.259 | -1.993 | 0.263 |
| *Streptococcus mitis* | Time of milk sampling: Night | 0.383 | 0.610 | 0.533 | 0.629 | -0.813 | 1.579 |
| *Streptococcus mitis* | Zn | 0.001 | 0.001 | 0.344 | 0.495 | -0.001 | 0.002 |
| *Streptococcus mitis* | Lactose | 0.017 | 0.021 | 0.410 | 0.563 | -0.024 | 0.058 |
| *Streptococcus mitis* | Lactoferrin | 0.199 | 0.249 | 0.428 | 0.563 | -0.289 | 0.687 |
| *Streptococcus mitis* | Lysozyme | -0.988 | 0.941 | 0.300 | 0.460 | -2.833 | 0.857 |
| *Streptococcus mitis* | sIgA | -0.282 | 1.164 | 0.809 | 0.866 | -2.563 | 1.999 |
| *Streptococcus mitis* | Delivery mode: emergency caesarean | 0.517 | 0.692 | 0.460 | 0.572 | -0.840 | 1.873 |
| *Streptococcus mitis* | Delivery mode: planned caesarean | 0.963 | 0.627 | 0.132 | 0.255 | -0.266 | 2.192 |
| *Streptococcus mitis* | First week pacifier: yes | -0.328 | 0.567 | 0.566 | 0.635 | -1.439 | 0.783 |
| *Streptococcus mitis* | Siblings: yes | 0.288 | 0.578 | 0.621 | 0.680 | -0.846 | 1.421 |
| *Streptococcus mitis* | Pre-pregnancy BMI: high and obese | -0.464 | 0.576 | 0.424 | 0.563 | -1.593 | 0.664 |
| *Gemella haemolysans* | Ca | 0.005 | 0.009 | 0.592 | 0.801 | -0.012 | 0.022 |
| *Gemella haemolysans* | Na | 0.003 | 0.007 | 0.728 | 0.881 | -0.012 | 0.017 |
| *Gemella haemolysans* | K | 0.000 | 0.006 | 0.980 | 0.982 | -0.012 | 0.013 |
| *Gemella haemolysans* | P | 0.004 | 0.022 | 0.860 | 0.920 | -0.039 | 0.046 |
| *Gemella haemolysans* | Mg | 0.108 | 0.055 | 0.056 | 0.099 | 0.000 | 0.216 |
| *Gemella haemolysans* | I | -0.004 | 0.005 | 0.452 | 0.647 | -0.013 | 0.006 |
| *Gemella haemolysans* | Se | -0.030 | 0.082 | 0.718 | 0.881 | -0.191 | 0.131 |
| *Gemella haemolysans* | Cu | -0.008 | 0.007 | 0.203 | 0.334 | -0.021 | 0.004 |
| *Gemella haemolysans* | Mn | -0.279 | 0.378 | 0.464 | 0.647 | -1.019 | 0.461 |
| *Gemella haemolysans* | Mo | 0.212 | 0.181 | 0.249 | 0.394 | -0.143 | 0.566 |
| *Gemella haemolysans* | Fe | 0.001 | 0.003 | 0.695 | 0.881 | -0.004 | 0.006 |
| *Gemella haemolysans* | Time of milk sampling: Afternoon | -2.845 | 0.855 | 0.002 | **0.005** | -4.520 | -1.170 |
| *Gemella haemolysans* | Time of milk sampling: Night | -2.579 | 0.906 | 0.007 | **0.014** | -4.355 | -0.803 |
| *Gemella haemolysans* | Zn | 0.000 | 0.001 | 0.982 | 0.982 | -0.002 | 0.002 |
| *Gemella haemolysans* | Lactose | -0.009 | 0.034 | 0.803 | 0.900 | -0.075 | 0.058 |
| *Gemella haemolysans* | Lactoferrin | 0.019 | 0.404 | 0.964 | 0.982 | -0.774 | 0.811 |
| *Gemella haemolysans* | Lysozyme | 3.593 | 1.435 | 0.016 | **0.032** | 0.779 | 6.406 |
| *Gemella haemolysans* | sIgA | 4.380 | 1.754 | 0.016 | **0.032** | 0.942 | 7.819 |
| *Gemella haemolysans* | Delivery mode: emergency caesarean | -2.633 | 1.066 | 0.018 | **0.032** | -4.721 | -0.544 |
| *Gemella haemolysans* | Delivery mode: planned caesarean | 0.277 | 0.965 | 0.775 | 0.900 | -1.615 | 2.170 |
| *Gemella haemolysans* | First week pacifier: yes | 0.188 | 0.942 | 0.843 | 0.920 | -1.659 | 2.034 |
| *Gemella haemolysans* | Siblings: yes | 1.677 | 0.899 | 0.069 | 0.118 | -0.086 | 3.440 |
| *Gemella haemolysans* | Pre-pregnancy BMI: high and obese | 0.258 | 0.937 | 0.784 | 0.900 | -1.578 | 2.095 |
| *Rothia mucilaginosa* | Ca | -0.004 | 0.008 | 0.605 | 0.679 | -0.021 | 0.012 |
| *Rothia mucilaginosa* | Na | -0.013 | 0.007 | 0.058 | 0.105 | -0.027 | 0.000 |
| *Rothia mucilaginosa* | K | -0.004 | 0.006 | 0.552 | 0.635 | -0.016 | 0.008 |
| *Rothia mucilaginosa* | P | -0.021 | 0.021 | 0.312 | 0.388 | -0.062 | 0.019 |
| *Rothia mucilaginosa* | Mg | -0.075 | 0.054 | 0.175 | 0.251 | -0.181 | 0.031 |
| *Rothia mucilaginosa* | I | 0.006 | 0.005 | 0.216 | 0.284 | -0.003 | 0.015 |
| *Rothia mucilaginosa* | Se | -0.026 | 0.079 | 0.746 | 0.780 | -0.182 | 0.130 |
| *Rothia mucilaginosa* | Cu | 0.002 | 0.006 | 0.779 | 0.796 | -0.011 | 0.014 |
| *Rothia mucilaginosa* | Mn | -0.560 | 0.357 | 0.124 | 0.190 | -1.260 | 0.139 |
| *Rothia mucilaginosa* | Mo | -0.300 | 0.172 | 0.087 | 0.141 | -0.637 | 0.036 |
| *Rothia mucilaginosa* | Fe | -0.006 | 0.003 | 0.038 | 0.080 | -0.011 | 0.000 |
| *Rothia mucilaginosa* | Time of milk sampling: Afternoon | 0.118 | 0.884 | 0.895 | 0.895 | -1.615 | 1.850 |
| *Rothia mucilaginosa* | Time of milk sampling: Night | 1.709 | 0.937 | 0.076 | 0.129 | -0.127 | 3.545 |
| *Rothia mucilaginosa* | Zn | 0.000 | 0.001 | 0.643 | 0.688 | -0.002 | 0.001 |
| *Rothia mucilaginosa* | Lactose | 0.040 | 0.032 | 0.216 | 0.284 | -0.023 | 0.104 |
| *Rothia mucilaginosa* | Lactoferrin | -0.308 | 0.388 | 0.432 | 0.510 | -1.068 | 0.453 |
| *Rothia mucilaginosa* | Lysozyme | -3.987 | 1.354 | 0.005 | **0.011** | -6.642 | -1.333 |
| *Rothia mucilaginosa* | sIgA | -2.260 | 1.781 | 0.211 | 0.284 | -5.751 | 1.230 |
| *Rothia mucilaginosa* | Delivery mode: emergency caesarean | 1.856 | 1.065 | 0.089 | 0.141 | -0.232 | 3.944 |
| *Rothia mucilaginosa* | Delivery mode: planned caesarean | 1.053 | 0.965 | 0.282 | 0.360 | -0.839 | 2.945 |
| *Rothia mucilaginosa* | First week pacifier: yes | 1.693 | 0.874 | 0.060 | 0.105 | -0.020 | 3.406 |
| *Rothia mucilaginosa* | Siblings: yes | -1.744 | 0.863 | 0.050 | 0.099 | -3.436 | -0.051 |
| *Rothia mucilaginosa* | Pre-pregnancy BMI: high and obese | 0.778 | 0.875 | 0.379 | 0.459 | -0.936 | 2.492 |
| *Streptococcus salivarius* group 1 | Ca | 0.003 | 0.013 | 0.839 | 0.896 | -0.023 | 0.029 |
| *Streptococcus salivarius* group 1 | Na | -0.016 | 0.011 | 0.150 | 0.300 | -0.037 | 0.005 |
| *Streptococcus salivarius* group 1 | K | 0.001 | 0.010 | 0.919 | 0.919 | -0.018 | 0.020 |
| *Streptococcus salivarius* group 1 | P | -0.020 | 0.033 | 0.539 | 0.681 | -0.084 | 0.044 |
| *Streptococcus salivarius* group 1 | Mg | -0.057 | 0.086 | 0.510 | 0.671 | -0.227 | 0.112 |
| *Streptococcus salivarius* group 1 | I | -0.004 | 0.007 | 0.547 | 0.681 | -0.018 | 0.010 |
| *Streptococcus salivarius* group 1 | Se | -0.158 | 0.122 | 0.202 | 0.355 | -0.398 | 0.081 |
| *Streptococcus salivarius* group 1 | Cu | 0.002 | 0.010 | 0.857 | 0.896 | -0.018 | 0.022 |
| *Streptococcus salivarius* group 1 | Mn | -1.065 | 0.552 | 0.060 | 0.146 | -2.146 | 0.016 |
| *Streptococcus salivarius* group 1 | Mo | -0.032 | 0.278 | 0.909 | 0.919 | -0.577 | 0.513 |
| *Streptococcus salivarius* group 1 | Fe | -0.008 | 0.004 | 0.052 | 0.133 | -0.016 | 0.000 |
| *Streptococcus salivarius* group 1 | Time of milk sampling: Afternoon | 2.357 | 1.384 | 0.096 | 0.211 | -0.356 | 5.069 |
| *Streptococcus salivarius* group 1 | Time of milk sampling: Night | -0.985 | 1.467 | 0.506 | 0.671 | -3.860 | 1.890 |
| *Streptococcus salivarius* group 1 | Zn | -0.001 | 0.001 | 0.349 | 0.517 | -0.004 | 0.001 |
| *Streptococcus salivarius* group 1 | Lactose | -0.027 | 0.051 | 0.600 | 0.708 | -0.127 | 0.073 |
| *Streptococcus salivarius* group 1 | Lactoferrin | -0.896 | 0.596 | 0.140 | 0.294 | -2.064 | 0.273 |
| *Streptococcus salivarius* group 1 | Lysozyme | -2.913 | 2.282 | 0.209 | 0.355 | -7.386 | 1.560 |
| *Streptococcus salivarius* group 1 | sIgA | -2.271 | 2.820 | 0.425 | 0.611 | -7.798 | 3.255 |
| *Streptococcus salivarius* group 1 | Delivery mode: emergency caesarean | 1.934 | 1.699 | 0.261 | 0.401 | -1.395 | 5.264 |
| *Streptococcus salivarius* group 1 | Delivery mode: planned caesarean | -0.862 | 1.539 | 0.579 | 0.700 | -3.878 | 2.155 |
| *Streptococcus salivarius* group 1 | First week pacifier: yes | 1.643 | 1.426 | 0.256 | 0.401 | -1.153 | 4.438 |
| *Streptococcus salivarius* group 1 | Siblings: yes | -0.980 | 1.407 | 0.490 | 0.671 | -3.738 | 1.778 |
| *Streptococcus salivarius* group 1 | Pre-pregnancy BMI: high and obese | -0.381 | 1.392 | 0.786 | 0.881 | -3.109 | 2.346 |
| *Veillonella* sp. | Ca | 0.013 | 0.013 | 0.332 | 0.558 | -0.013 | 0.038 |
| *Veillonella* sp. | Na | 0.001 | 0.011 | 0.900 | 0.920 | -0.020 | 0.023 |
| *Veillonella* sp. | K | -0.002 | 0.010 | 0.860 | 0.899 | -0.021 | 0.017 |
| *Veillonella* sp. | P | -0.008 | 0.033 | 0.805 | 0.861 | -0.072 | 0.056 |
| *Veillonella* sp. | Mg | 0.161 | 0.084 | 0.060 | 0.153 | -0.002 | 0.325 |
| *Veillonella* sp. | I | -0.016 | 0.007 | 0.025 | 0.077 | -0.029 | -0.002 |
| *Veillonella* sp. | Se | -0.086 | 0.124 | 0.493 | 0.656 | -0.329 | 0.157 |
| *Veillonella* sp. | Cu | 0.003 | 0.010 | 0.731 | 0.820 | -0.016 | 0.023 |
| *Veillonella* sp. | Mn | -0.519 | 0.571 | 0.368 | 0.558 | -1.638 | 0.599 |
| *Veillonella* sp. | Mo | -0.290 | 0.275 | 0.298 | 0.548 | -0.829 | 0.249 |
| *Veillonella* sp. | Fe | -0.005 | 0.004 | 0.237 | 0.454 | -0.014 | 0.003 |
| *Veillonella* sp. | Time of milk sampling: Afternoon | -1.219 | 1.466 | 0.411 | 0.581 | -4.092 | 1.654 |
| *Veillonella* sp. | Time of milk sampling: Night | -1.432 | 1.553 | 0.362 | 0.558 | -4.477 | 1.612 |
| *Veillonella* sp. | Zn | 0.001 | 0.001 | 0.417 | 0.581 | -0.002 | 0.004 |
| *Veillonella* sp. | Lactose | 0.015 | 0.051 | 0.779 | 0.853 | -0.086 | 0.115 |
| *Veillonella* sp. | Lactoferrin | -0.253 | 0.612 | 0.681 | 0.797 | -1.452 | 0.946 |
| *Veillonella* sp. | Lysozyme | -3.304 | 2.274 | 0.154 | 0.323 | -7.761 | 1.153 |
| *Veillonella* sp. | sIgA | 0.021 | 2.846 | 0.994 | 0.994 | -5.557 | 5.599 |
| *Veillonella* sp. | Delivery mode: emergency caesarean | -3.526 | 1.639 | 0.037 | 0.107 | -6.739 | -0.313 |
| *Veillonella* sp. | Delivery mode: planned caesarean | 0.590 | 1.485 | 0.693 | 0.797 | -2.321 | 3.501 |
| *Veillonella* sp. | First week pacifier: yes | -1.276 | 1.425 | 0.376 | 0.558 | -4.069 | 1.518 |
| *Veillonella* sp. | Siblings: yes | -1.282 | 1.404 | 0.366 | 0.558 | -4.034 | 1.470 |
| *Veillonella* sp. | Pre-pregnancy BMI: high and obese | 1.910 | 1.391 | 0.177 | 0.354 | -0.817 | 4.637 |
| *Neisseria subflava* | Ca | -0.003 | 0.011 | 0.747 | 0.904 | -0.024 | 0.017 |
| *Neisseria subflava* | Na | 0.002 | 0.009 | 0.815 | 0.914 | -0.015 | 0.020 |
| *Neisseria subflava* | K | 0.009 | 0.008 | 0.236 | 0.638 | -0.006 | 0.024 |
| *Neisseria subflava* | P | 0.025 | 0.026 | 0.347 | 0.648 | -0.026 | 0.076 |
| *Neisseria subflava* | Mg | 0.084 | 0.068 | 0.229 | 0.638 | -0.051 | 0.218 |
| *Neisseria subflava* | I | 0.004 | 0.006 | 0.503 | 0.772 | -0.007 | 0.015 |
| *Neisseria subflava* | Se | 0.057 | 0.100 | 0.569 | 0.786 | -0.138 | 0.252 |
| *Neisseria subflava* | Cu | 0.000 | 0.008 | 0.958 | 0.958 | -0.016 | 0.015 |
| *Neisseria subflava* | Mn | 0.429 | 0.456 | 0.352 | 0.648 | -0.465 | 1.324 |
| *Neisseria subflava* | Mo | 0.199 | 0.221 | 0.372 | 0.659 | -0.234 | 0.632 |
| *Neisseria subflava* | Fe | 0.004 | 0.003 | 0.201 | 0.638 | -0.002 | 0.011 |
| *Neisseria subflava* | Time of milk sampling: Afternoon | -0.843 | 1.176 | 0.477 | 0.771 | -3.147 | 1.461 |
| *Neisseria subflava* | Time of milk sampling: Night | 0.657 | 1.246 | 0.601 | 0.790 | -1.785 | 3.099 |
| *Neisseria subflava* | Zn | 0.000 | 0.001 | 0.736 | 0.904 | -0.003 | 0.002 |
| *Neisseria subflava* | Lactose | -0.012 | 0.041 | 0.770 | 0.908 | -0.093 | 0.069 |
| *Neisseria subflava* | Lactoferrin | -0.284 | 0.489 | 0.564 | 0.786 | -1.241 | 0.674 |
| *Neisseria subflava* | Lysozyme | 0.121 | 1.864 | 0.949 | 0.958 | -3.533 | 3.774 |
| *Neisseria subflava* | sIgA | 0.400 | 2.277 | 0.861 | 0.943 | -4.062 | 4.863 |
| *Neisseria subflava* | Delivery mode: emergency caesarean | -1.517 | 1.346 | 0.266 | 0.638 | -4.155 | 1.121 |
| *Neisseria subflava* | Delivery mode: planned caesarean | 1.281 | 1.220 | 0.299 | 0.638 | -1.109 | 3.672 |
| *Neisseria subflava* | First week pacifier: yes | 0.063 | 1.145 | 0.956 | 0.958 | -2.180 | 2.307 |
| *Neisseria subflava* | Siblings: yes | 0.874 | 1.127 | 0.442 | 0.753 | -1.334 | 3.082 |
| *Neisseria subflava* | Pre-pregnancy BMI: high and obese | 2.179 | 1.094 | 0.053 | 0.311 | 0.036 | 4.323 |
| *Streptococcus oralis* | Ca | -0.004 | 0.014 | 0.796 | 0.880 | -0.031 | 0.024 |
| *Streptococcus oralis* | Na | -0.018 | 0.011 | 0.130 | 0.248 | -0.040 | 0.005 |
| *Streptococcus oralis* | K | -0.011 | 0.010 | 0.291 | 0.432 | -0.030 | 0.009 |
| *Streptococcus oralis* | P | -0.017 | 0.035 | 0.616 | 0.709 | -0.085 | 0.050 |
| *Streptococcus oralis* | Mg | -0.174 | 0.088 | 0.056 | 0.142 | -0.347 | -0.001 |
| *Streptococcus oralis* | I | -0.001 | 0.008 | 0.879 | 0.917 | -0.016 | 0.014 |
| *Streptococcus oralis* | Se | -0.003 | 0.132 | 0.979 | 0.979 | -0.262 | 0.256 |
| *Streptococcus oralis* | Cu | 0.006 | 0.011 | 0.565 | 0.692 | -0.015 | 0.027 |
| *Streptococcus oralis* | Mn | 0.080 | 0.610 | 0.897 | 0.917 | -1.115 | 1.274 |
| *Streptococcus oralis* | Mo | -0.379 | 0.289 | 0.198 | 0.337 | -0.946 | 0.188 |
| *Streptococcus oralis* | Fe | -0.003 | 0.005 | 0.582 | 0.692 | -0.011 | 0.006 |
| *Streptococcus oralis* | Time of milk sampling: Afternoon | 1.378 | 1.547 | 0.378 | 0.544 | -1.655 | 4.411 |
| *Streptococcus oralis* | Time of milk sampling: Night | 2.322 | 1.640 | 0.165 | 0.303 | -0.893 | 5.536 |
| *Streptococcus oralis* | Zn | 0.001 | 0.001 | 0.445 | 0.585 | -0.002 | 0.004 |
| *Streptococcus oralis* | Lactose | 0.090 | 0.053 | 0.097 | 0.222 | -0.014 | 0.193 |
| *Streptococcus oralis* | Lactoferrin | -0.151 | 0.648 | 0.817 | 0.880 | -1.422 | 1.120 |
| *Streptococcus oralis* | Lysozyme | -3.849 | 2.394 | 0.115 | 0.248 | -8.542 | 0.844 |
| *Streptococcus oralis* | sIgA | -6.664 | 2.836 | 0.023 | 0.072 | -12.222 | -1.106 |
| *Streptococcus oralis* | Delivery mode: emergency caesarean | 1.431 | 1.827 | 0.438 | 0.585 | -2.149 | 5.011 |
| *Streptococcus oralis* | Delivery mode: planned caesarean | 0.907 | 1.655 | 0.586 | 0.692 | -2.336 | 4.151 |
| *Streptococcus oralis* | First week pacifier: yes | -1.731 | 1.436 | 0.235 | 0.361 | -4.546 | 1.084 |
| *Streptococcus oralis* | Siblings: yes | -1.809 | 1.475 | 0.227 | 0.361 | -4.700 | 1.081 |
| *Streptococcus oralis* | Pre-pregnancy BMI: high and obese | -1.240 | 1.489 | 0.410 | 0.571 | -4.158 | 1.678 |
| *Haemophilus haemolyticus* | Ca | -0.011 | 0.014 | 0.451 | 0.691 | -0.039 | 0.017 |
| *Haemophilus haemolyticus* | Na | -0.001 | 0.012 | 0.952 | 0.969 | -0.025 | 0.023 |
| *Haemophilus haemolyticus* | K | 0.007 | 0.010 | 0.536 | 0.796 | -0.014 | 0.027 |
| *Haemophilus haemolyticus* | P | 0.074 | 0.034 | 0.036 | 0.117 | 0.007 | 0.141 |
| *Haemophilus haemolyticus* | Mg | 0.092 | 0.094 | 0.334 | 0.568 | -0.092 | 0.276 |
| *Haemophilus haemolyticus* | I | 0.002 | 0.008 | 0.775 | 0.944 | -0.013 | 0.018 |
| *Haemophilus haemolyticus* | Se | 0.264 | 0.130 | 0.049 | 0.133 | 0.009 | 0.519 |
| *Haemophilus haemolyticus* | Cu | 0.006 | 0.011 | 0.615 | 0.868 | -0.016 | 0.027 |
| *Haemophilus haemolyticus* | Mn | 1.262 | 0.599 | 0.041 | 0.126 | 0.088 | 2.437 |
| *Haemophilus haemolyticus* | Mo | 0.054 | 0.304 | 0.859 | 0.969 | -0.542 | 0.651 |
| *Haemophilus haemolyticus* | Fe | 0.009 | 0.005 | 0.061 | 0.156 | 0.000 | 0.018 |
| *Haemophilus haemolyticus* | Time of milk sampling: Afternoon | -1.762 | 1.548 | 0.262 | 0.482 | -4.795 | 1.272 |
| *Haemophilus haemolyticus* | Time of milk sampling: Night | -0.393 | 1.640 | 0.812 | 0.958 | -3.608 | 2.822 |
| *Haemophilus haemolyticus* | Zn | 0.000 | 0.002 | 0.780 | 0.944 | -0.003 | 0.003 |
| *Haemophilus haemolyticus* | Lactose | -0.004 | 0.056 | 0.939 | 0.969 | -0.115 | 0.106 |
| *Haemophilus haemolyticus* | Lactoferrin | 0.026 | 0.670 | 0.969 | 0.969 | -1.287 | 1.339 |
| *Haemophilus haemolyticus* | Lysozyme | 4.489 | 2.451 | 0.074 | 0.170 | -0.315 | 9.294 |
| *Haemophilus haemolyticus* | sIgA | 5.530 | 2.993 | 0.072 | 0.170 | -0.336 | 11.397 |
| *Haemophilus haemolyticus* | Delivery mode: emergency caesarean | -1.615 | 1.884 | 0.396 | 0.651 | -5.309 | 2.078 |
| *Haemophilus haemolyticus* | Delivery mode: planned caesarean | 0.178 | 1.707 | 0.917 | 0.969 | -3.168 | 3.524 |
| *Haemophilus haemolyticus* | First week pacifier: yes | 0.240 | 1.537 | 0.876 | 0.969 | -2.772 | 3.253 |
| *Haemophilus haemolyticus* | Siblings: yes | 0.546 | 1.547 | 0.726 | 0.944 | -2.486 | 3.577 |
| *Haemophilus haemolyticus* | Pre-pregnancy BMI: high and obese | 0.112 | 1.566 | 0.943 | 0.969 | -2.956 | 3.181 |
| *Veillonella nakazawae* | Ca | 0.000 | 0.010 | 0.994 | 0.994 | -0.020 | 0.020 |
| *Veillonella nakazawae* | Na | -0.016 | 0.008 | 0.068 | 0.125 | -0.032 | 0.001 |
| *Veillonella nakazawae* | K | -0.011 | 0.007 | 0.141 | 0.232 | -0.025 | 0.003 |
| *Veillonella nakazawae* | P | -0.057 | 0.024 | 0.024 | **0.047** | -0.104 | -0.009 |
| *Veillonella nakazawae* | Mg | -0.101 | 0.067 | 0.136 | 0.232 | -0.231 | 0.029 |
| *Veillonella nakazawae* | I | -0.007 | 0.006 | 0.194 | 0.288 | -0.018 | 0.004 |
| *Veillonella nakazawae* | Se | -0.222 | 0.092 | 0.020 | **0.042** | -0.402 | -0.042 |
| *Veillonella nakazawae* | Cu | 0.000 | 0.008 | 0.986 | 0.994 | -0.016 | 0.015 |
| *Veillonella nakazawae* | Mn | -0.446 | 0.447 | 0.324 | 0.447 | -1.322 | 0.430 |
| *Veillonella nakazawae* | Mo | -0.027 | 0.219 | 0.903 | 0.944 | -0.455 | 0.402 |
| *Veillonella nakazawae* | Fe | -0.007 | 0.003 | 0.032 | 0.061 | -0.013 | -0.001 |
| *Veillonella nakazawae* | Time of milk sampling: Afternoon | 1.432 | 1.082 | 0.193 | 0.288 | -0.689 | 3.553 |
| *Veillonella nakazawae* | Time of milk sampling: Night | 2.068 | 1.147 | 0.079 | 0.139 | -0.179 | 4.316 |
| *Veillonella nakazawae* | Zn | -0.001 | 0.001 | 0.460 | 0.556 | -0.003 | 0.001 |
| *Veillonella nakazawae* | Lactose | -0.011 | 0.040 | 0.792 | 0.847 | -0.090 | 0.068 |
| *Veillonella nakazawae* | Lactoferrin | -0.677 | 0.470 | 0.157 | 0.249 | -1.598 | 0.244 |
| *Veillonella nakazawae* | Lysozyme | -4.150 | 1.715 | 0.020 | **0.042** | -7.511 | -0.789 |
| *Veillonella nakazawae* | sIgA | -2.547 | 2.199 | 0.253 | 0.364 | -6.857 | 1.764 |
| *Veillonella nakazawae* | Delivery mode: emergency caesarean | 0.830 | 1.355 | 0.544 | 0.625 | -1.826 | 3.486 |
| *Veillonella nakazawae* | Delivery mode: planned caesarean | -0.518 | 1.228 | 0.675 | 0.740 | -2.925 | 1.889 |
| *Veillonella nakazawae* | First week pacifier: yes | 0.821 | 1.129 | 0.471 | 0.556 | -1.392 | 3.034 |
| *Veillonella nakazawae* | Siblings: yes | -1.041 | 1.101 | 0.349 | 0.447 | -3.199 | 1.116 |
| *Veillonella nakazawae* | Pre-pregnancy BMI: high and obese | -1.066 | 1.107 | 0.341 | 0.447 | -3.236 | 1.104 |
| *Staphylococcus lugdunensis* | Ca | -0.007 | 0.007 | 0.318 | 0.731 | -0.020 | 0.007 |
| *Staphylococcus lugdunensis* | Na | -0.004 | 0.006 | 0.542 | 0.949 | -0.015 | 0.008 |
| *Staphylococcus lugdunensis* | K | -0.001 | 0.005 | 0.907 | 0.949 | -0.010 | 0.009 |
| *Staphylococcus lugdunensis* | P | -0.004 | 0.017 | 0.821 | 0.949 | -0.038 | 0.030 |
| *Staphylococcus lugdunensis* | Mg | 0.010 | 0.046 | 0.827 | 0.949 | -0.080 | 0.100 |
| *Staphylococcus lugdunensis* | I | 0.007 | 0.004 | 0.049 | 0.298 | 0.000 | 0.014 |
| *Staphylococcus lugdunensis* | Se | -0.009 | 0.066 | 0.893 | 0.949 | -0.137 | 0.120 |
| *Staphylococcus lugdunensis* | Cu | 0.001 | 0.005 | 0.876 | 0.949 | -0.010 | 0.011 |
| *Staphylococcus lugdunensis* | Mn | -0.075 | 0.302 | 0.806 | 0.949 | -0.668 | 0.518 |
| *Staphylococcus lugdunensis* | Mo | 0.098 | 0.146 | 0.506 | 0.949 | -0.188 | 0.383 |
| *Staphylococcus lugdunensis* | Fe | 0.000 | 0.002 | 0.841 | 0.949 | -0.005 | 0.004 |
| *Staphylococcus lugdunensis* | Time of milk sampling: Afternoon | -0.821 | 0.787 | 0.303 | 0.731 | -2.364 | 0.721 |
| *Staphylococcus lugdunensis* | Time of milk sampling: Night | -0.076 | 0.834 | 0.928 | 0.949 | -1.711 | 1.558 |
| *Staphylococcus lugdunensis* | Zn | 0.001 | 0.001 | 0.256 | 0.731 | -0.001 | 0.002 |
| *Staphylococcus lugdunensis* | Lactose | 0.043 | 0.026 | 0.105 | 0.438 | -0.008 | 0.095 |
| *Staphylococcus lugdunensis* | Lactoferrin | 0.496 | 0.313 | 0.120 | 0.460 | -0.117 | 1.110 |
| *Staphylococcus lugdunensis* | Lysozyme | 2.372 | 1.169 | 0.049 | 0.298 | 0.082 | 4.663 |
| *Staphylococcus lugdunensis* | sIgA | -0.376 | 1.494 | 0.802 | 0.949 | -3.304 | 2.552 |
| *Staphylococcus lugdunensis* | Delivery mode: emergency caesarean | 0.301 | 0.913 | 0.743 | 0.949 | -1.489 | 2.092 |
| *Staphylococcus lugdunensis* | Delivery mode: planned caesarean | 0.138 | 0.828 | 0.868 | 0.949 | -1.484 | 1.760 |
| *Staphylococcus lugdunensis* | First week pacifier: yes | 0.908 | 0.746 | 0.230 | 0.706 | -0.553 | 2.369 |
| *Staphylococcus lugdunensis* | Siblings: yes | 0.175 | 0.744 | 0.815 | 0.949 | -1.283 | 1.634 |
| *Staphylococcus lugdunensis* | Pre-pregnancy BMI: high and obese | -0.332 | 0.743 | 0.657 | 0.949 | -1.788 | 1.123 |
| *Haemophilus parainfluenzae* | Ca | -0.005 | 0.011 | 0.618 | 0.805 | -0.026 | 0.015 |
| *Haemophilus parainfluenzae* | Na | -0.005 | 0.009 | 0.577 | 0.805 | -0.022 | 0.012 |
| *Haemophilus parainfluenzae* | K | 0.005 | 0.008 | 0.511 | 0.805 | -0.010 | 0.020 |
| *Haemophilus parainfluenzae* | P | -0.002 | 0.026 | 0.952 | 0.973 | -0.053 | 0.050 |
| *Haemophilus parainfluenzae* | Mg | 0.067 | 0.069 | 0.337 | 0.805 | -0.068 | 0.201 |
| *Haemophilus parainfluenzae* | I | 0.003 | 0.006 | 0.630 | 0.805 | -0.008 | 0.014 |
| *Haemophilus parainfluenzae* | Se | 0.041 | 0.099 | 0.683 | 0.826 | -0.154 | 0.235 |
| *Haemophilus parainfluenzae* | Cu | -0.004 | 0.008 | 0.605 | 0.805 | -0.020 | 0.012 |
| *Haemophilus parainfluenzae* | Mn | -0.385 | 0.455 | 0.403 | 0.805 | -1.278 | 0.508 |
| *Haemophilus parainfluenzae* | Mo | 0.007 | 0.222 | 0.975 | 0.975 | -0.428 | 0.443 |
| *Haemophilus parainfluenzae* | Fe | 0.001 | 0.004 | 0.852 | 0.973 | -0.006 | 0.008 |
| *Haemophilus parainfluenzae* | Time of milk sampling: Afternoon | -0.957 | 1.194 | 0.428 | 0.805 | -3.297 | 1.384 |
| *Haemophilus parainfluenzae* | Time of milk sampling: Night | 0.501 | 1.266 | 0.694 | 0.826 | -1.980 | 2.982 |
| *Haemophilus parainfluenzae* | Zn | 0.000 | 0.001 | 0.700 | 0.826 | -0.002 | 0.003 |
| *Haemophilus parainfluenzae* | Lactose | 0.004 | 0.041 | 0.920 | 0.973 | -0.076 | 0.085 |
| *Haemophilus parainfluenzae* | Lactoferrin | -0.552 | 0.481 | 0.257 | 0.740 | -1.496 | 0.391 |
| *Haemophilus parainfluenzae* | Lysozyme | 1.267 | 1.847 | 0.496 | 0.805 | -2.353 | 4.886 |
| *Haemophilus parainfluenzae* | sIgA | 3.794 | 2.194 | 0.091 | 0.418 | -0.506 | 8.094 |
| *Haemophilus parainfluenzae* | Delivery mode: emergency caesarean | -1.254 | 1.343 | 0.356 | 0.805 | -3.886 | 1.379 |
| *Haemophilus parainfluenzae* | Delivery mode: planned caesarean | 1.437 | 1.217 | 0.244 | 0.740 | -0.948 | 3.822 |
| *Haemophilus parainfluenzae* | First week pacifier: yes | 0.640 | 1.139 | 0.577 | 0.805 | -1.593 | 2.872 |
| *Haemophilus parainfluenzae* | Siblings: yes | -0.149 | 1.130 | 0.896 | 0.973 | -2.363 | 2.066 |
| *Haemophilus parainfluenzae* | Pre-pregnancy BMI: high and obese | 0.580 | 1.125 | 0.608 | 0.805 | -1.624 | 2.784 |
| Unclassified *Flavobacteriaceae* | Ca | 0.036 | 0.011 | 0.003 | **0.034** | 0.014 | 0.059 |
| Unclassified *Flavobacteriaceae* | Na | 0.000 | 0.011 | 0.993 | 0.993 | -0.021 | 0.021 |
| Unclassified *Flavobacteriaceae* | K | 0.012 | 0.009 | 0.203 | 0.445 | -0.006 | 0.029 |
| Unclassified *Flavobacteriaceae* | P | 0.005 | 0.032 | 0.864 | 0.947 | -0.056 | 0.067 |
| Unclassified *Flavobacteriaceae* | Mg | 0.129 | 0.081 | 0.120 | 0.324 | -0.030 | 0.289 |
| Unclassified *Flavobacteriaceae* | I | 0.000 | 0.007 | 0.982 | 0.993 | -0.014 | 0.013 |
| Unclassified *Flavobacteriaceae* | Se | -0.002 | 0.120 | 0.984 | 0.993 | -0.238 | 0.233 |
| Unclassified *Flavobacteriaceae* | Cu | 0.019 | 0.009 | 0.048 | 0.182 | 0.001 | 0.037 |
| Unclassified *Flavobacteriaceae* | Mn | -0.617 | 0.546 | 0.264 | 0.528 | -1.687 | 0.452 |
| Unclassified *Flavobacteriaceae* | Mo | 0.188 | 0.266 | 0.484 | 0.696 | -0.334 | 0.710 |
| Unclassified *Flavobacteriaceae* | Fe | -0.003 | 0.004 | 0.418 | 0.660 | -0.012 | 0.005 |
| Unclassified *Flavobacteriaceae* | Time of milk sampling: Afternoon | 1.019 | 1.442 | 0.484 | 0.696 | -1.808 | 3.846 |
| Unclassified *Flavobacteriaceae* | Time of milk sampling: Night | 0.386 | 1.529 | 0.802 | 0.900 | -2.611 | 3.382 |
| Unclassified *Flavobacteriaceae* | Zn | -0.001 | 0.001 | 0.319 | 0.578 | -0.004 | 0.001 |
| Unclassified *Flavobacteriaceae* | Lactose | -0.021 | 0.049 | 0.670 | 0.857 | -0.118 | 0.076 |
| Unclassified *Flavobacteriaceae* | Lactoferrin | -0.182 | 0.588 | 0.759 | 0.900 | -1.335 | 0.971 |
| Unclassified *Flavobacteriaceae* | Lysozyme | -1.942 | 2.219 | 0.386 | 0.653 | -6.291 | 2.407 |
| Unclassified *Flavobacteriaceae* | sIgA | -5.216 | 2.617 | 0.053 | 0.186 | -10.346 | -0.086 |
| Unclassified *Flavobacteriaceae* | Delivery mode: emergency caesarean | 0.499 | 1.621 | 0.760 | 0.900 | -2.678 | 3.675 |
| Unclassified *Flavobacteriaceae* | Delivery mode: planned caesarean | -2.261 | 1.468 | 0.131 | 0.335 | -5.139 | 0.617 |
| Unclassified *Flavobacteriaceae* | First week pacifier: yes | -1.815 | 1.364 | 0.191 | 0.439 | -4.489 | 0.860 |
| Unclassified *Flavobacteriaceae* | Siblings: yes | -0.863 | 1.356 | 0.528 | 0.736 | -3.521 | 1.795 |
| Unclassified *Flavobacteriaceae* | Pre-pregnancy BMI: high and obese | 0.358 | 1.378 | 0.797 | 0.900 | -2.343 | 3.058 |
| *Streptococcus parasanguinis* | Ca | 0.003 | 0.012 | 0.835 | 0.875 | -0.021 | 0.026 |
| *Streptococcus parasanguinis* | Na | -0.004 | 0.010 | 0.695 | 0.787 | -0.024 | 0.016 |
| *Streptococcus parasanguinis* | K | 0.004 | 0.009 | 0.643 | 0.779 | -0.013 | 0.021 |
| *Streptococcus parasanguinis* | P | 0.019 | 0.030 | 0.534 | 0.713 | -0.040 | 0.077 |
| *Streptococcus parasanguinis* | Mg | 0.093 | 0.078 | 0.240 | 0.525 | -0.060 | 0.247 |
| *Streptococcus parasanguinis* | I | -0.007 | 0.007 | 0.289 | 0.567 | -0.020 | 0.006 |
| *Streptococcus parasanguinis* | Se | -0.109 | 0.113 | 0.342 | 0.629 | -0.330 | 0.113 |
| *Streptococcus parasanguinis* | Cu | -0.006 | 0.009 | 0.486 | 0.696 | -0.024 | 0.012 |
| *Streptococcus parasanguinis* | Mn | -0.455 | 0.523 | 0.389 | 0.677 | -1.479 | 0.570 |
| *Streptococcus parasanguinis* | Mo | -0.178 | 0.254 | 0.486 | 0.696 | -0.675 | 0.319 |
| *Streptococcus parasanguinis* | Fe | -0.008 | 0.004 | 0.056 | 0.148 | -0.015 | 0.000 |
| *Streptococcus parasanguinis* | Time of milk sampling: Afternoon | 0.507 | 1.311 | 0.701 | 0.787 | -2.064 | 3.077 |
| *Streptococcus parasanguinis* | Time of milk sampling: Night | 0.161 | 1.390 | 0.909 | 0.929 | -2.564 | 2.885 |
| *Streptococcus parasanguinis* | Zn | 0.001 | 0.001 | 0.447 | 0.696 | -0.002 | 0.004 |
| *Streptococcus parasanguinis* | Lactose | -0.029 | 0.047 | 0.543 | 0.713 | -0.121 | 0.063 |
| *Streptococcus parasanguinis* | Lactoferrin | -0.705 | 0.551 | 0.207 | 0.502 | -1.784 | 0.374 |
| *Streptococcus parasanguinis* | Lysozyme | -1.445 | 2.120 | 0.499 | 0.696 | -5.601 | 2.711 |
| *Streptococcus parasanguinis* | sIgA | 2.959 | 2.565 | 0.255 | 0.533 | -2.069 | 7.987 |
| *Streptococcus parasanguinis* | Delivery mode: emergency caesarean | 0.032 | 1.580 | 0.984 | 0.984 | -3.064 | 3.129 |
| *Streptococcus parasanguinis* | Delivery mode: planned caesarean | -1.194 | 1.431 | 0.409 | 0.677 | -4.000 | 1.612 |
| *Streptococcus parasanguinis* | First week pacifier: yes | 2.471 | 1.268 | 0.058 | 0.148 | -0.014 | 4.957 |
| *Streptococcus parasanguinis* | Siblings: yes | -1.066 | 1.287 | 0.412 | 0.677 | -3.589 | 1.456 |
| *Streptococcus parasanguinis* | Pre-pregnancy BMI: high and obese | -0.387 | 1.301 | 0.767 | 0.841 | -2.938 | 2.163 |
| *Bifidobacterium longum* | Ca | 0.005 | 0.008 | 0.518 | 0.907 | -0.010 | 0.020 |
| *Bifidobacterium longum* | Na | -0.006 | 0.006 | 0.331 | 0.663 | -0.019 | 0.006 |
| *Bifidobacterium longum* | K | 0.002 | 0.006 | 0.727 | 0.907 | -0.009 | 0.013 |
| *Bifidobacterium longum* | P | 0.001 | 0.019 | 0.977 | 0.992 | -0.037 | 0.038 |
| *Bifidobacterium longum* | Mg | 0.011 | 0.051 | 0.833 | 0.939 | -0.089 | 0.111 |
| *Bifidobacterium longum* | I | -0.007 | 0.004 | 0.084 | 0.387 | -0.015 | 0.001 |
| *Bifidobacterium longum* | Se | -0.037 | 0.073 | 0.620 | 0.907 | -0.180 | 0.107 |
| *Bifidobacterium longum* | Cu | 0.002 | 0.006 | 0.793 | 0.936 | -0.010 | 0.013 |
| *Bifidobacterium longum* | Mn | -0.364 | 0.334 | 0.281 | 0.647 | -1.019 | 0.290 |
| *Bifidobacterium longum* | Mo | -0.203 | 0.161 | 0.214 | 0.547 | -0.518 | 0.112 |
| *Bifidobacterium longum* | Fe | -0.003 | 0.003 | 0.262 | 0.635 | -0.008 | 0.002 |
| *Bifidobacterium longum* | Time of milk sampling: Afternoon | 0.719 | 0.865 | 0.411 | 0.787 | -0.977 | 2.416 |
| *Bifidobacterium longum* | Time of milk sampling: Night | -0.555 | 0.917 | 0.548 | 0.907 | -2.353 | 1.243 |
| *Bifidobacterium longum* | Zn | 0.000 | 0.001 | 0.921 | 0.963 | -0.002 | 0.002 |
| *Bifidobacterium longum* | Lactose | 0.018 | 0.030 | 0.562 | 0.907 | -0.041 | 0.077 |
| *Bifidobacterium longum* | Lactoferrin | -0.462 | 0.353 | 0.197 | 0.547 | -1.154 | 0.230 |
| *Bifidobacterium longum* | Lysozyme | -0.563 | 1.366 | 0.682 | 0.907 | -3.240 | 2.114 |
| *Bifidobacterium longum* | sIgA | -1.219 | 1.662 | 0.467 | 0.860 | -4.476 | 2.038 |
| *Bifidobacterium longum* | Delivery mode: emergency caesarean | 2.137 | 0.961 | 0.031 | 0.223 | 0.255 | 4.020 |
| *Bifidobacterium longum* | Delivery mode: planned caesarean | -0.292 | 0.870 | 0.739 | 0.907 | -1.997 | 1.414 |
| *Bifidobacterium longum* | First week pacifier: yes | 0.420 | 0.807 | 0.606 | 0.907 | -1.163 | 2.002 |
| *Bifidobacterium longum* | Siblings: yes | -0.089 | 0.833 | 0.915 | 0.963 | -1.721 | 1.543 |
| *Bifidobacterium longum* | Pre-pregnancy BMI: high and obese | 0.411 | 0.815 | 0.617 | 0.907 | -1.187 | 2.009 |
| *Lactobacillus gasseri* | Ca | 0.005 | 0.011 | 0.664 | 0.825 | -0.016 | 0.025 |
| *Lactobacillus gasseri* | Na | -0.001 | 0.009 | 0.914 | 0.967 | -0.019 | 0.017 |
| *Lactobacillus gasseri* | K | 0.004 | 0.008 | 0.582 | 0.766 | -0.011 | 0.019 |
| *Lactobacillus gasseri* | P | 0.002 | 0.026 | 0.925 | 0.967 | -0.049 | 0.054 |
| *Lactobacillus gasseri* | Mg | -0.075 | 0.069 | 0.285 | 0.504 | -0.210 | 0.060 |
| *Lactobacillus gasseri* | I | -0.007 | 0.006 | 0.199 | 0.412 | -0.019 | 0.004 |
| *Lactobacillus gasseri* | Se | -0.079 | 0.099 | 0.433 | 0.642 | -0.274 | 0.116 |
| *Lactobacillus gasseri* | Cu | 0.012 | 0.008 | 0.142 | 0.345 | -0.004 | 0.027 |
| *Lactobacillus gasseri* | Mn | -0.455 | 0.457 | 0.325 | 0.554 | -1.350 | 0.441 |
| *Lactobacillus gasseri* | Mo | -0.282 | 0.219 | 0.206 | 0.412 | -0.712 | 0.148 |
| *Lactobacillus gasseri* | Fe | -0.003 | 0.003 | 0.417 | 0.642 | -0.010 | 0.004 |
| *Lactobacillus gasseri* | Time of milk sampling: Afternoon | 2.072 | 1.167 | 0.084 | 0.282 | -0.216 | 4.360 |
| *Lactobacillus gasseri* | Time of milk sampling: Night | 0.915 | 1.237 | 0.464 | 0.659 | -1.509 | 3.340 |
| *Lactobacillus gasseri* | Zn | -0.002 | 0.001 | 0.173 | 0.399 | -0.004 | 0.001 |
| *Lactobacillus gasseri* | Lactose | -0.062 | 0.040 | 0.128 | 0.345 | -0.141 | 0.016 |
| *Lactobacillus gasseri* | Lactoferrin | -0.822 | 0.475 | 0.091 | 0.282 | -1.754 | 0.110 |
| *Lactobacillus gasseri* | Lysozyme | -2.207 | 1.838 | 0.236 | 0.435 | -5.810 | 1.396 |
| *Lactobacillus gasseri* | sIgA | -2.784 | 2.243 | 0.221 | 0.424 | -7.181 | 1.613 |
| *Lactobacillus gasseri* | Delivery mode: emergency caesarean | 1.100 | 1.386 | 0.432 | 0.642 | -1.617 | 3.818 |
| *Lactobacillus gasseri* | Delivery mode: planned caesarean | 0.067 | 1.256 | 0.958 | 0.979 | -2.396 | 2.529 |
| *Lactobacillus gasseri* | First week pacifier: yes | -0.608 | 1.151 | 0.600 | 0.767 | -2.863 | 1.647 |
| *Lactobacillus gasseri* | Siblings: yes | 0.026 | 1.137 | 0.982 | 0.982 | -2.203 | 2.255 |
| *Lactobacillus gasseri* | Pre-pregnancy BMI: high and obese | -0.265 | 1.149 | 0.819 | 0.941 | -2.517 | 1.986 |

Ca, calcium; Na, sodium; K, potassium; P, phosphorus; Mg, magnesium; I, iodine; Se, selenium; Cu, copper; Mn, manganese; Mo, molybdenum; Fe, iron; Zn, zinc; sIgA, secretory immunoglobulin A.

**Supplementary Table S8**. Outputs from covariate-adjusted linear regression models assessing the associations between human milk component intakes and the infant oral microbiome at 3 months postpartum.

| **Response variable** | **Explanatory variables** | **Estimate** | **Standard Error** | **P-value** | **BH corrected P-value** | **Lower CI** | **Upper CI** |
| --- | --- | --- | --- | --- | --- | --- | --- |
| Shannon diversity | Ca | -0.000003 | 0.000001 | 0.044 | 0.083 | -0.0000053 | -0.0000002 |
| Shannon diversity | Na | -0.000004 | 0.000002 | 0.021 | **0.041** | -0.0000074 | -0.0000008 |
| Shannon diversity | K | -0.000001 | 0.000001 | 0.059 | 0.106 | -0.0000028 | 0.0000000 |
| Shannon diversity | P | -0.000005 | 0.000003 | 0.098 | 0.163 | -0.0000103 | 0.0000007 |
| Shannon diversity | Mg | -0.000012 | 0.000013 | 0.365 | 0.432 | -0.0000366 | 0.0000132 |
| Shannon diversity | I | -0.000002 | 0.000001 | 0.011 | **0.022** | -0.0000041 | -0.0000006 |
| Shannon diversity | Se | -0.000016 | 0.000018 | 0.364 | 0.432 | -0.0000513 | 0.0000186 |
| Shannon diversity | Cu | -0.000001 | 0.000001 | 0.218 | 0.282 | -0.0000038 | 0.0000008 |
| Shannon diversity | Mn | -0.000130 | 0.000102 | 0.207 | 0.282 | -0.0003294 | 0.0000688 |
| Shannon diversity | Mo | -0.000019 | 0.000047 | 0.683 | 0.732 | -0.0001121 | 0.0000732 |
| Shannon diversity | Fe | -0.000001 | 0.000001 | 0.135 | 0.203 | -0.0000026 | 0.0000003 |
| Shannon diversity | Time of milk sampling: Afternoon | 0.005798 | 0.235433 | 0.980 | 0.980 | -0.4556520 | 0.4672473 |
| Shannon diversity | Time of milk sampling: Night | -0.113836 | 0.226406 | 0.618 | 0.678 | -0.5575919 | 0.3299189 |
| Shannon diversity | Zn | 0.000000 | 0.000000 | 0.119 | 0.185 | -0.0000009 | 0.0000001 |
| Shannon diversity | Lactose | -0.000009 | 0.000005 | 0.110 | 0.177 | -0.0000191 | 0.0000017 |
| Shannon diversity | Lactoferrin | -0.000265 | 0.000081 | 0.002 | **0.005** | -0.0004248 | -0.0001058 |
| Shannon diversity | Lysozyme | -0.000137 | 0.000463 | 0.769 | 0.805 | -0.0010454 | 0.0007714 |
| Shannon diversity | sIgA | 0.000706 | 0.000565 | 0.219 | 0.282 | -0.0004020 | 0.0018134 |
| Shannon diversity | Delivery mode: emergency caesarean | -0.480652 | 0.261436 | 0.074 | 0.128 | -0.9930677 | 0.0317629 |
| Shannon diversity | Delivery mode: planned caesarean | -0.329586 | 0.261436 | 0.215 | 0.282 | -0.8420012 | 0.1828294 |
| Shannon diversity | First week pacifier: yes | 0.295641 | 0.210446 | 0.168 | 0.244 | -0.1168336 | 0.7081152 |
| Shannon diversity | Siblings: yes | -0.187873 | 0.218722 | 0.396 | 0.456 | -0.6165674 | 0.2408215 |
| Shannon diversity | Pre-pregnancy BMI: high and obese | 0.008862 | 0.228911 | 0.969 | 0.980 | -0.4398032 | 0.4575265 |
| Richness | Ca | -0.000435 | 0.000249 | 0.089 | 0.153 | -0.0009238 | 0.0000531 |
| Richness | Na | -0.000738 | 0.000319 | 0.026 | 0.054 | -0.0013637 | -0.0001123 |
| Richness | K | -0.000223 | 0.000135 | 0.106 | 0.177 | -0.0004868 | 0.0000412 |
| Richness | P | -0.000791 | 0.000533 | 0.146 | 0.218 | -0.0018359 | 0.0002533 |
| Richness | Mg | -0.002321 | 0.002376 | 0.335 | 0.408 | -0.0069783 | 0.0023360 |
| Richness | I | -0.000378 | 0.000169 | 0.032 | 0.062 | -0.0007102 | -0.0000458 |
| Richness | Se | -0.002965 | 0.003340 | 0.380 | 0.439 | -0.0095118 | 0.0035812 |
| Richness | Cu | -0.000215 | 0.000221 | 0.335 | 0.408 | -0.0006473 | 0.0002171 |
| Richness | Mn | -0.027252 | 0.018930 | 0.158 | 0.222 | -0.0643556 | 0.0098514 |
| Richness | Mo | -0.002140 | 0.008868 | 0.811 | 0.848 | -0.0195222 | 0.0152419 |
| Richness | Fe | -0.000219 | 0.000135 | 0.114 | 0.182 | -0.0004832 | 0.0000460 |
| Richness | Time of milk sampling: Afternoon | -8.287256 | 43.385612 | 0.850 | 0.869 | -93.3230 | 76.7485438 |
| Richness | Time of milk sampling: Night | -48.145241 | 41.721996 | 0.256 | 0.339 | -129.9203 | 33.6298708 |
| Richness | Zn | -0.000085 | 0.000046 | 0.068 | 0.123 | -0.0001746 | 0.0000039 |
| Richness | Lactose | -0.001594 | 0.000996 | 0.117 | 0.182 | -0.0035454 | 0.0003575 |
| Richness | Lactoferrin | -0.039587 | 0.015985 | 0.018 | 0.380 | -0.0709181 | -0.0082564 |
| Richness | Lysozyme | -0.026629 | 0.086803 | 0.761 | 0.815 | -0.1967629 | 0.1435051 |
| Richness | sIgA | 0.118318 | 0.106278 | 0.272 | 0.350 | -0.0899873 | 0.3266231 |
| Richness | Delivery mode: emergency caesarean | -94.084339 | 48.525077 | 0.060 | 0.112 | -189.19349 | 1.02481 |
| Richness | Delivery mode: planned caesarean | -71.054339 | 48.525077 | 0.151 | 0.220 | -166.16349 | 24.0548128 |
| Richness | First week pacifier: yes | 55.186846 | 39.603529 | 0.172 | 0.234 | -22.43607 | 132.8097638 |
| Richness | Siblings: yes | -19.431968 | 41.236977 | 0.640 | 0.703 | -100.25644 | 61.3925072 |
| Richness | Pre-pregnancy BMI: high and obese | 4.126491 | 42.659126 | 0.923 | 0.923 | -79.48540 | 87.7383772 |
| *Streptococcus mitis* | Ca | 0.000005 | 0.000004 | 0.179 | 0.350 | -0.0000022 | 0.0000122 |
| *Streptococcus mitis* | Na | 0.000006 | 0.000005 | 0.213 | 0.368 | -0.0000034 | 0.0000156 |
| *Streptococcus mitis* | K | 0.000002 | 0.000002 | 0.313 | 0.440 | -0.0000019 | 0.0000059 |
| *Streptococcus mitis* | P | 0.000007 | 0.000008 | 0.363 | 0.494 | -0.0000082 | 0.0000226 |
| *Streptococcus mitis* | Mg | 0.000015 | 0.000035 | 0.664 | 0.740 | -0.0000529 | 0.0000834 |
| *Streptococcus mitis* | I | 0.000003 | 0.000003 | 0.229 | 0.368 | -0.0000019 | 0.0000081 |
| *Streptococcus mitis* | Se | 0.000036 | 0.000049 | 0.466 | 0.567 | -0.0000594 | 0.0001309 |
| *Streptococcus mitis* | Cu | 0.000000 | 0.000003 | 0.926 | 0.926 | -0.0000066 | 0.0000060 |
| *Streptococcus mitis* | Mn | 0.000211 | 0.000280 | 0.454 | 0.567 | -0.0003366 | 0.0007590 |
| *Streptococcus mitis* | Mo | 0.000106 | 0.000127 | 0.409 | 0.525 | -0.0001434 | 0.0003564 |
| *Streptococcus mitis* | Fe | 0.000004 | 0.000002 | 0.054 | 0.115 | 0.0000001 | 0.0000076 |
| *Streptococcus mitis* | Time of milk sampling: Afternoon | -0.558413 | 0.619450 | 0.373 | 0.494 | -1.7725350 | 0.6557095 |
| *Streptococcus mitis* | Time of milk sampling: Night | 0.364616 | 0.595697 | 0.544 | 0.644 | -0.8029509 | 1.5321828 |
| *Streptococcus mitis* | Zn | 0.000001 | 0.000001 | 0.227 | 0.368 | -0.0000005 | 0.0000022 |
| *Streptococcus mitis* | Lactose | 0.000018 | 0.000015 | 0.217 | 0.368 | -0.0000103 | 0.0000470 |
| *Streptococcus mitis* | Lactoferrin | 0.000438 | 0.000239 | 0.075 | 0.153 | -0.0000307 | 0.0009068 |
| *Streptococcus mitis* | Lysozyme | -0.000601 | 0.001256 | 0.635 | 0.733 | -0.0030623 | 0.0018606 |
| *Streptococcus mitis* | sIgA | -0.000392 | 0.001563 | 0.803 | 0.835 | -0.0034557 | 0.0026723 |
| *Streptococcus mitis* | Delivery mode: emergency caesarean | 0.789581 | 0.726600 | 0.284 | 0.425 | -0.6345546 | 2.2137162 |
| *Streptococcus mitis* | Delivery mode: planned caesarean | 0.911331 | 0.726600 | 0.217 | 0.368 | -0.5128046 | 2.3354662 |
| *Streptococcus mitis* | First week pacifier: yes | -0.249328 | 0.589084 | 0.675 | 0.740 | -1.4039324 | 0.9052760 |
| *Streptococcus mitis* | Siblings: yes | 0.139717 | 0.598915 | 0.817 | 0.835 | -1.0341570 | 1.3135912 |
| *Streptococcus mitis* | Pre-pregnancy BMI: high and obese | -0.697484 | 0.612257 | 0.262 | 0.406 | -1.8975082 | 0.5025409 |
| *Gemella haemolysans* | Ca | -0.000008 | 0.000006 | 0.181 | 0.247 | -0.0000193 | 0.0000035 |
| *Gemella haemolysans* | Na | -0.000002 | 0.000008 | 0.785 | 0.803 | -0.0000175 | 0.0000132 |
| *Gemella haemolysans* | K | -0.000005 | 0.000003 | 0.106 | 0.159 | -0.0000112 | 0.0000009 |
| *Gemella haemolysans* | P | -0.000023 | 0.000012 | 0.065 | 0.109 | -0.0000465 | 0.0000008 |
| *Gemella haemolysans* | Mg | -0.000016 | 0.000055 | 0.777 | 0.803 | -0.0001242 | 0.0000926 |
| *Gemella haemolysans* | I | -0.000007 | 0.000004 | 0.090 | 0.139 | -0.0000148 | 0.0000009 |
| *Gemella haemolysans* | Se | -0.000096 | 0.000076 | 0.217 | 0.287 | -0.0002448 | 0.0000536 |
| *Gemella haemolysans* | Cu | -0.000011 | 0.000005 | 0.036 | 0.067 | -0.0000201 | -0.0000010 |
| *Gemella haemolysans* | Mn | -0.000505 | 0.000440 | 0.258 | 0.331 | -0.0013671 | 0.0003569 |
| *Gemella haemolysans* | Mo | 0.000127 | 0.000203 | 0.537 | 0.671 | -0.0002717 | 0.0005251 |
| *Gemella haemolysans* | Fe | -0.000001 | 0.000003 | 0.781 | 0.803 | -0.0000065 | 0.0000049 |
| *Gemella haemolysans* | Time of milk sampling: Afternoon | -2.355940 | 0.935161 | 0.016 | **0.033** | -4.1888548 | -0.5230247 |
| *Gemella haemolysans* | Time of milk sampling: Night | -2.234117 | 0.899302 | 0.018 | **0.034** | -3.9967496 | -0.4714852 |
| *Gemella haemolysans* | Zn | -0.000002 | 0.000001 | 0.049 | 0.088 | -0.0000042 | -0.0000001 |
| *Gemella haemolysans* | Lactose | -0.000040 | 0.000023 | 0.087 | 0.139 | -0.0000846 | 0.0000047 |
| *Gemella haemolysans* | Lactoferrin | -0.000173 | 0.000395 | 0.663 | 0.768 | -0.0009471 | 0.0006007 |
| *Gemella haemolysans* | Lysozyme | 0.003092 | 0.001938 | 0.119 | 0.172 | -0.0007070 | 0.0068907 |
| *Gemella haemolysans* | sIgA | 0.003691 | 0.002414 | 0.134 | 0.189 | -0.0010396 | 0.0084214 |
| *Gemella haemolysans* | Delivery mode: emergency caesarean | -3.039150 | 1.082194 | 0.008 | **0.017** | -5.1602498 | -0.9180507 |
| *Gemella haemolysans* | Delivery mode: planned caesarean | -0.577189 | 1.082194 | 0.597 | 0.726 | -2.6982881 | 1.5439110 |
| *Gemella haemolysans* | First week pacifier: yes | 0.385655 | 0.935012 | 0.682 | 0.768 | -1.4469689 | 2.2182791 |
| *Gemella haemolysans* | Siblings: yes | 1.729457 | 0.910701 | 0.065 | 0.109 | -0.0555171 | 3.5144321 |
| *Gemella haemolysans* | Pre-pregnancy BMI: high and obese | -0.018394 | 0.994054 | 0.985 | 0.985 | -1.9667404 | 1.9299520 |
| *Rothia mucilaginosa* | Ca | -0.000005 | 0.000005 | 0.366 | 0.445 | -0.0000157 | 0.0000057 |
| *Rothia mucilaginosa* | Na | -0.000015 | 0.000007 | 0.034 | 0.069 | -0.0000286 | -0.0000017 |
| *Rothia mucilaginosa* | K | -0.000002 | 0.000003 | 0.462 | 0.520 | -0.0000080 | 0.0000036 |
| *Rothia mucilaginosa* | P | -0.000011 | 0.000012 | 0.341 | 0.445 | -0.0000339 | 0.0000115 |
| *Rothia mucilaginosa* | Mg | -0.000046 | 0.000051 | 0.375 | 0.445 | -0.0001454 | 0.0000541 |
| *Rothia mucilaginosa* | I | 0.000002 | 0.000004 | 0.528 | 0.566 | -0.0000051 | 0.0000099 |
| *Rothia mucilaginosa* | Se | -0.000056 | 0.000072 | 0.438 | 0.505 | -0.0001964 | 0.0000842 |
| *Rothia mucilaginosa* | Cu | -0.000002 | 0.000005 | 0.737 | 0.772 | -0.0000110 | 0.0000077 |
| *Rothia mucilaginosa* | Mn | -0.000781 | 0.000396 | 0.056 | 0.100 | -0.0015578 | -0.0000046 |
| *Rothia mucilaginosa* | Mo | -0.000265 | 0.000185 | 0.159 | 0.231 | -0.0006281 | 0.0000971 |
| *Rothia mucilaginosa* | Fe | -0.000006 | 0.000003 | 0.044 | 0.083 | -0.0000110 | -0.0000003 |
| *Rothia mucilaginosa* | Time of milk sampling: Afternoon | -0.587124 | 0.873510 | 0.506 | 0.555 | -2.2992031 | 1.1249558 |
| *Rothia mucilaginosa* | Time of milk sampling: Night | 1.231164 | 0.840015 | 0.151 | 0.227 | -0.4152660 | 2.8775938 |
| *Rothia mucilaginosa* | Zn | 0.000000 | 0.000001 | 0.972 | 0.972 | -0.0000020 | 0.0000020 |
| *Rothia mucilaginosa* | Lactose | 0.000006 | 0.000022 | 0.771 | 0.789 | -0.0000366 | 0.0000495 |
| *Rothia mucilaginosa* | Lactoferrin | -0.000338 | 0.000364 | 0.359 | 0.445 | -0.0010510 | 0.0003750 |
| *Rothia mucilaginosa* | Lysozyme | -0.003890 | 0.001751 | 0.032 | 0.069 | -0.0073227 | -0.0004579 |
| *Rothia mucilaginosa* | sIgA | -0.004239 | 0.002207 | 0.062 | 0.103 | -0.0085642 | 0.0000866 |
| *Rothia mucilaginosa* | Delivery mode: emergency caesarean | 1.825027 | 1.050139 | 0.090 | 0.145 | -0.2332460 | 3.8833001 |
| *Rothia mucilaginosa* | Delivery mode: planned caesarean | 1.375412 | 1.050139 | 0.198 | 0.279 | -0.6828610 | 3.4336851 |
| *Rothia mucilaginosa* | First week pacifier: yes | 1.382005 | 0.836659 | 0.107 | 0.166 | -0.2578477 | 3.0218567 |
| *Rothia mucilaginosa* | Siblings: yes | -1.641891 | 0.844481 | 0.059 | 0.102 | -3.2970726 | 0.0132916 |
| *Rothia mucilaginosa* | Pre-pregnancy BMI: high and obese | 0.977397 | 0.874246 | 0.271 | 0.369 | -0.7361259 | 2.6909197 |
| *Streptococcus salivarius* group 1 | Ca | -0.000003 | 0.000009 | 0.768 | 0.843 | -0.0000199 | 0.0000146 |
| *Streptococcus salivarius* group 1 | Na | -0.000017 | 0.000011 | 0.132 | 0.258 | -0.0000394 | 0.0000047 |
| *Streptococcus salivarius* group 1 | K | -0.000002 | 0.000005 | 0.618 | 0.776 | -0.0000117 | 0.0000069 |
| *Streptococcus salivarius* group 1 | P | -0.000012 | 0.000019 | 0.506 | 0.695 | -0.0000488 | 0.0000239 |
| *Streptococcus salivarius* group 1 | Mg | -0.000081 | 0.000081 | 0.323 | 0.472 | -0.0002396 | 0.0000775 |
| *Streptococcus salivarius* group 1 | I | -0.000004 | 0.000006 | 0.525 | 0.695 | -0.0000159 | 0.0000080 |
| *Streptococcus salivarius* group 1 | Se | -0.000113 | 0.000113 | 0.324 | 0.472 | -0.0003359 | 0.0001090 |
| *Streptococcus salivarius* group 1 | Cu | 0.000001 | 0.000008 | 0.886 | 0.929 | -0.0000138 | 0.0000160 |
| *Streptococcus salivarius* group 1 | Mn | -0.001087 | 0.000639 | 0.097 | 0.198 | -0.0023389 | 0.0001643 |
| *Streptococcus salivarius* group 1 | Mo | -0.000037 | 0.000302 | 0.904 | 0.929 | -0.0006294 | 0.0005557 |
| *Streptococcus salivarius* group 1 | Fe | -0.000008 | 0.000004 | 0.083 | 0.179 | -0.0000164 | 0.0000008 |
| *Streptococcus salivarius* group 1 | Time of milk sampling: Afternoon | 2.000400 | 1.408558 | 0.164 | 0.307 | -0.7603742 | 4.7611745 |
| *Streptococcus salivarius* group 1 | Time of milk sampling: Night | -1.844180 | 1.354547 | 0.182 | 0.314 | -4.4990929 | 0.8107327 |
| *Streptococcus salivarius* group 1 | Zn | -0.000002 | 0.000002 | 0.179 | 0.314 | -0.0000053 | 0.0000009 |
| *Streptococcus salivarius* group 1 | Lactose | -0.000017 | 0.000035 | 0.638 | 0.776 | -0.0000850 | 0.0000519 |
| *Streptococcus salivarius* group 1 | Lactoferrin | -0.000283 | 0.000584 | 0.631 | 0.776 | -0.0014280 | 0.0008618 |
| *Streptococcus salivarius* group 1 | Lysozyme | -0.003638 | 0.002903 | 0.218 | 0.350 | -0.0093283 | 0.0020521 |
| *Streptococcus salivarius* group 1 | sIgA | -0.004552 | 0.003605 | 0.214 | 0.350 | -0.0116179 | 0.0025142 |
| *Streptococcus salivarius* group 1 | Delivery mode: emergency caesarean | 0.671883 | 1.753134 | 0.704 | 0.833 | -2.7642587 | 4.1080254 |
| *Streptococcus salivarius* group 1 | Delivery mode: planned caesarean | -0.587712 | 1.753134 | 0.739 | 0.833 | -4.0238536 | 2.8484305 |
| *Streptococcus salivarius* group 1 | First week pacifier: yes | 1.365332 | 1.368748 | 0.325 | 0.472 | -1.3174141 | 4.0480790 |
| *Streptococcus salivarius* group 1 | Siblings: yes | 0.162396 | 1.408739 | 0.909 | 0.929 | -2.5987334 | 2.9235250 |
| *Streptococcus salivarius* group 1 | Pre-pregnancy BMI: high and obese | -0.026245 | 1.464093 | 0.986 | 0.986 | -2.8958669 | 2.8433762 |
| *Veillonella* sp. | Ca | -0.000011 | 0.000009 | 0.243 | 0.331 | -0.0000282 | 0.0000069 |
| *Veillonella* sp. | Na | -0.000009 | 0.000012 | 0.431 | 0.508 | -0.0000328 | 0.0000138 |
| *Veillonella* sp. | K | -0.000008 | 0.000005 | 0.109 | 0.182 | -0.0000171 | 0.0000015 |
| *Veillonella* sp. | P | -0.000034 | 0.000018 | 0.070 | 0.125 | -0.0000707 | 0.0000017 |
| *Veillonella* sp. | Mg | -0.000026 | 0.000085 | 0.764 | 0.782 | -0.0001913 | 0.0001402 |
| *Veillonella* sp. | I | -0.000017 | 0.000006 | 0.006 | **0.012** | -0.0000280 | -0.0000055 |
| *Veillonella* sp. | Se | -0.000189 | 0.000115 | 0.108 | 0.182 | -0.0004141 | 0.0000361 |
| *Veillonella* sp. | Cu | -0.000011 | 0.000008 | 0.148 | 0.229 | -0.0000263 | 0.0000037 |
| *Veillonella* sp. | Mn | -0.000722 | 0.000674 | 0.291 | 0.363 | -0.0020433 | 0.0005990 |
| *Veillonella* sp. | Mo | -0.000483 | 0.000303 | 0.119 | 0.191 | -0.0010760 | 0.0001108 |
| *Veillonella* sp. | Fe | -0.000009 | 0.000005 | 0.052 | 0.102 | -0.0000187 | -0.0000002 |
| *Veillonella* sp. | Time of milk sampling: Afternoon | 0.332798 | 1.516099 | 0.827 | 0.827 | -2.6387555 | 3.3043521 |
| *Veillonella* sp. | Time of milk sampling: Night | -0.995241 | 1.457964 | 0.499 | 0.548 | -3.8528505 | 1.8623694 |
| *Veillonella* sp. | Zn | -0.000001 | 0.000002 | 0.665 | 0.696 | -0.0000040 | 0.0000025 |
| *Veillonella* sp. | Lactose | -0.000040 | 0.000036 | 0.267 | 0.343 | -0.0001099 | 0.0000297 |
| *Veillonella* sp. | Lactoferrin | -0.000705 | 0.000595 | 0.243 | 0.331 | -0.0018708 | 0.0004607 |
| *Veillonella* sp. | Lysozyme | -0.006622 | 0.002870 | 0.026 | 0.054 | -0.0122472 | -0.0009970 |
| *Veillonella* sp. | sIgA | -0.004356 | 0.003736 | 0.251 | 0.332 | -0.0116781 | 0.0029668 |
| *Veillonella* sp. | Delivery mode: emergency caesarean | -3.419906 | 1.729466 | 0.055 | 0.104 | -6.8096605 | -0.0301519 |
| *Veillonella* sp. | Delivery mode: planned caesarean | -1.180721 | 1.729466 | 0.499 | 0.548 | -4.5704750 | 2.2090337 |
| *Veillonella* sp. | First week pacifier: yes | -1.079188 | 1.382846 | 0.440 | 0.508 | -3.7895665 | 1.6311897 |
| *Veillonella* sp. | Siblings: yes | -2.027372 | 1.419209 | 0.161 | 0.234 | -4.8090226 | 0.7542776 |
| *Veillonella* sp. | Pre-pregnancy BMI: high and obese | 1.458220 | 1.502745 | 0.338 | 0.411 | -1.4871608 | 4.4036003 |
| *Neisseria subflava* | Ca | 0.000003 | 0.000007 | 0.692 | 0.841 | -0.0000114 | 0.0000173 |
| *Neisseria subflava* | Na | 0.000007 | 0.000010 | 0.501 | 0.806 | -0.0000123 | 0.0000253 |
| *Neisseria subflava* | K | 0.000004 | 0.000004 | 0.298 | 0.639 | -0.0000035 | 0.0000117 |
| *Neisseria subflava* | P | 0.000017 | 0.000015 | 0.259 | 0.639 | -0.0000124 | 0.0000474 |
| *Neisseria subflava* | Mg | 0.000109 | 0.000066 | 0.106 | 0.433 | -0.0000201 | 0.0002380 |
| *Neisseria subflava* | I | 0.000003 | 0.000005 | 0.602 | 0.817 | -0.0000073 | 0.0000126 |
| *Neisseria subflava* | Se | 0.000079 | 0.000095 | 0.407 | 0.678 | -0.0001062 | 0.0002650 |
| *Neisseria subflava* | Cu | 0.000002 | 0.000006 | 0.770 | 0.858 | -0.0000105 | 0.0000142 |
| *Neisseria subflava* | Mn | 0.000893 | 0.000531 | 0.101 | 0.433 | -0.0001485 | 0.0019340 |
| *Neisseria subflava* | Mo | 0.000264 | 0.000248 | 0.294 | 0.639 | -0.0002218 | 0.0007493 |
| *Neisseria subflava* | Fe | 0.000007 | 0.000004 | 0.092 | 0.433 | -0.0000009 | 0.0000142 |
| *Neisseria subflava* | Time of milk sampling: Afternoon | -0.590290 | 1.234885 | 0.635 | 0.817 | -3.0106655 | 1.8300847 |
| *Neisseria subflava* | Time of milk sampling: Night | 0.633042 | 1.187534 | 0.597 | 0.817 | -1.6945238 | 2.9606084 |
| *Neisseria subflava* | Zn | 0.000000 | 0.000001 | 0.820 | 0.858 | -0.0000023 | 0.0000029 |
| *Neisseria subflava* | Lactose | 0.000027 | 0.000029 | 0.361 | 0.668 | -0.0000298 | 0.0000830 |
| *Neisseria subflava* | Lactoferrin | -0.000120 | 0.000487 | 0.806 | 0.858 | -0.0010742 | 0.0008333 |
| *Neisseria subflava* | Lysozyme | 0.000800 | 0.002458 | 0.747 | 0.858 | -0.0040176 | 0.0056168 |
| *Neisseria subflava* | sIgA | 0.001733 | 0.003044 | 0.573 | 0.817 | -0.0042346 | 0.0076998 |
| *Neisseria subflava* | Delivery mode: emergency caesarean | -1.533964 | 1.421235 | 0.287 | 0.639 | -4.3195854 | 1.2516574 |
| *Neisseria subflava* | Delivery mode: planned caesarean | 1.210935 | 1.421235 | 0.400 | 0.678 | -1.5746865 | 3.9965563 |
| *Neisseria subflava* | First week pacifier: yes | 0.152237 | 1.148808 | 0.895 | 0.895 | -2.0994277 | 2.4039013 |
| *Neisseria subflava* | Siblings: yes | 0.733357 | 1.165193 | 0.533 | 0.807 | -1.5504211 | 3.0171346 |
| *Neisseria subflava* | Pre-pregnancy BMI: high and obese | 1.907033 | 1.184667 | 0.116 | 0.433 | -0.4149147 | 4.2289803 |
| *Streptococcus oralis* | Ca | 0.000013 | 0.000009 | 0.180 | 0.342 | -0.0000055 | 0.0000306 |
| *Streptococcus oralis* | Na | -0.000007 | 0.000012 | 0.551 | 0.670 | -0.0000315 | 0.0000167 |
| *Streptococcus oralis* | K | 0.000006 | 0.000005 | 0.274 | 0.425 | -0.0000042 | 0.0000153 |
| *Streptococcus oralis* | P | 0.000033 | 0.000019 | 0.090 | 0.226 | -0.0000043 | 0.0000709 |
| *Streptococcus oralis* | Mg | 0.000054 | 0.000087 | 0.535 | 0.669 | -0.0001160 | 0.0002250 |
| *Streptococcus oralis* | I | 0.000003 | 0.000007 | 0.661 | 0.744 | -0.0000099 | 0.0000157 |
| *Streptococcus oralis* | Se | 0.000081 | 0.000122 | 0.509 | 0.669 | -0.0001577 | 0.0003204 |
| *Streptococcus oralis* | Cu | 0.000012 | 0.000008 | 0.143 | 0.322 | -0.0000037 | 0.0000273 |
| *Streptococcus oralis* | Mn | 0.000366 | 0.000704 | 0.606 | 0.712 | -0.0010133 | 0.0017455 |
| *Streptococcus oralis* | Mo | -0.000202 | 0.000321 | 0.533 | 0.669 | -0.0008311 | 0.0004273 |
| *Streptococcus oralis* | Fe | 0.000001 | 0.000005 | 0.901 | 0.921 | -0.0000093 | 0.0000106 |
| *Streptococcus oralis* | Time of milk sampling: Afternoon | 0.046514 | 1.626073 | 0.977 | 0.977 | -3.1405896 | 3.2336168 |
| *Streptococcus oralis* | Time of milk sampling: Night | 1.775550 | 1.563721 | 0.263 | 0.423 | -1.2893442 | 4.8404440 |
| *Streptococcus oralis* | Zn | 0.000004 | 0.000002 | 0.011 | **0.037** | 0.0000011 | 0.0000073 |
| *Streptococcus oralis* | Lactose | 0.000085 | 0.000035 | 0.019 | 0.058 | 0.0000168 | 0.0001532 |
| *Streptococcus oralis* | Lactoferrin | 0.000134 | 0.000625 | 0.831 | 0.869 | -0.0010900 | 0.0013590 |
| *Streptococcus oralis* | Lysozyme | -0.001045 | 0.003155 | 0.742 | 0.795 | -0.0072289 | 0.0051381 |
| *Streptococcus oralis* | sIgA | -0.005104 | 0.003838 | 0.191 | 0.344 | -0.0126271 | 0.0024191 |
| *Streptococcus oralis* | Delivery mode: emergency caesarean | 1.623875 | 1.851156 | 0.386 | 0.560 | -2.0043913 | 5.2521405 |
| *Streptococcus oralis* | Delivery mode: planned caesarean | 1.355510 | 1.851156 | 0.469 | 0.639 | -2.2727564 | 4.9837755 |
| *Streptococcus oralis* | First week pacifier: yes | -1.989104 | 1.401221 | 0.164 | 0.335 | -4.7354980 | 0.7572901 |
| *Streptococcus oralis* | Siblings: yes | -1.995274 | 1.468980 | 0.182 | 0.342 | -4.8744758 | 0.8839275 |
| *Streptococcus oralis* | Pre-pregnancy BMI: high and obese | -0.603839 | 1.542083 | 0.698 | 0.766 | -3.6263224 | 2.4186438 |
| *Haemophilus haemolyticus* | Ca | -0.000005 | 0.000010 | 0.643 | 0.904 | -0.0000240 | 0.0000148 |
| *Haemophilus haemolyticus* | Na | 0.000000 | 0.000013 | 0.982 | 0.982 | -0.0000259 | 0.0000253 |
| *Haemophilus haemolyticus* | K | 0.000001 | 0.000005 | 0.878 | 0.957 | -0.0000097 | 0.0000113 |
| *Haemophilus haemolyticus* | P | 0.000022 | 0.000021 | 0.298 | 0.517 | -0.0000188 | 0.0000625 |
| *Haemophilus haemolyticus* | Mg | 0.000060 | 0.000092 | 0.515 | 0.859 | -0.0001197 | 0.0002402 |
| *Haemophilus haemolyticus* | I | -0.000001 | 0.000007 | 0.853 | 0.957 | -0.0000148 | 0.0000122 |
| *Haemophilus haemolyticus* | Se | 0.000202 | 0.000125 | 0.115 | 0.235 | -0.0000436 | 0.0004478 |
| *Haemophilus haemolyticus* | Cu | 0.000002 | 0.000009 | 0.844 | 0.957 | -0.0000151 | 0.0000185 |
| *Haemophilus haemolyticus* | Mn | 0.001554 | 0.000703 | 0.033 | 0.083 | 0.0001759 | 0.0029311 |
| *Haemophilus haemolyticus* | Mo | -0.000071 | 0.000340 | 0.836 | 0.957 | -0.0007380 | 0.0005965 |
| *Haemophilus haemolyticus* | Fe | 0.000008 | 0.000005 | 0.120 | 0.235 | -0.0000019 | 0.0000187 |
| *Haemophilus haemolyticus* | Time of milk sampling: Afternoon | -0.944767 | 1.689959 | 0.579 | 0.869 | -4.2570867 | 2.3675537 |
| *Haemophilus haemolyticus* | Time of milk sampling: Night | -0.177371 | 1.625158 | 0.914 | 0.957 | -3.3626807 | 3.0079386 |
| *Haemophilus haemolyticus* | Zn | 0.000001 | 0.000002 | 0.571 | 0.869 | -0.0000025 | 0.0000046 |
| *Haemophilus haemolyticus* | Lactose | 0.000004 | 0.000039 | 0.915 | 0.957 | -0.0000731 | 0.0000815 |
| *Haemophilus haemolyticus* | Lactoferrin | 0.000179 | 0.000659 | 0.787 | 0.957 | -0.0011131 | 0.0014716 |
| *Haemophilus haemolyticus* | Lysozyme | 0.006302 | 0.003179 | 0.055 | 0.129 | 0.0000709 | 0.0125331 |
| *Haemophilus haemolyticus* | sIgA | 0.005787 | 0.004038 | 0.160 | 0.300 | -0.0021283 | 0.0137016 |
| *Haemophilus haemolyticus* | Delivery mode: emergency caesarean | -1.105681 | 1.969798 | 0.578 | 0.869 | -4.9664858 | 2.7551239 |
| *Haemophilus haemolyticus* | Delivery mode: planned caesarean | -1.041866 | 1.969798 | 0.600 | 0.871 | -4.9026711 | 2.8189386 |
| *Haemophilus haemolyticus* | First week pacifier: yes | 0.563223 | 1.559438 | 0.720 | 0.926 | -2.4932746 | 3.6197214 |
| *Haemophilus haemolyticus* | Siblings: yes | -0.062547 | 1.587113 | 0.969 | 0.982 | -3.1732882 | 3.0481944 |
| *Haemophilus haemolyticus* | Pre-pregnancy BMI: high and obese | 0.232921 | 1.655560 | 0.889 | 0.957 | -3.0119760 | 3.4778179 |
| *Veillonella nakazawae* | Ca | 0.000004 | 0.000008 | 0.616 | 0.711 | -0.0000110 | 0.0000187 |
| *Veillonella nakazawae* | Na | -0.000012 | 0.000010 | 0.229 | 0.413 | -0.0000313 | 0.0000073 |
| *Veillonella nakazawae* | K | 0.000001 | 0.000004 | 0.891 | 0.911 | -0.0000075 | 0.0000086 |
| *Veillonella nakazawae* | P | -0.000003 | 0.000016 | 0.870 | 0.911 | -0.0000343 | 0.0000290 |
| *Veillonella nakazawae* | Mg | -0.000039 | 0.000071 | 0.588 | 0.706 | -0.0001769 | 0.0000997 |
| *Veillonella nakazawae* | I | -0.000003 | 0.000005 | 0.596 | 0.706 | -0.0000132 | 0.0000075 |
| *Veillonella nakazawae* | Se | -0.000120 | 0.000097 | 0.224 | 0.413 | -0.0003114 | 0.0000706 |
| *Veillonella nakazawae* | Cu | 0.000002 | 0.000007 | 0.749 | 0.843 | -0.0000107 | 0.0000150 |
| *Veillonella nakazawae* | Mn | -0.000459 | 0.000567 | 0.423 | 0.577 | -0.0015713 | 0.0006527 |
| *Veillonella nakazawae* | Mo | 0.000059 | 0.000261 | 0.822 | 0.881 | -0.0004528 | 0.0005710 |
| *Veillonella nakazawae* | Fe | -0.000005 | 0.000004 | 0.185 | 0.377 | -0.0000132 | 0.0000024 |
| *Veillonella nakazawae* | Time of milk sampling: Afternoon | 1.200336 | 1.281476 | 0.355 | 0.519 | -1.3113577 | 3.7120289 |
| *Veillonella nakazawae* | Time of milk sampling: Night | 1.540715 | 1.232338 | 0.219 | 0.413 | -0.8746681 | 3.9560974 |
| *Veillonella nakazawae* | Zn | 0.000000 | 0.000001 | 0.954 | 0.954 | -0.0000028 | 0.0000027 |
| *Veillonella nakazawae* | Lactose | 0.000017 | 0.000030 | 0.581 | 0.706 | -0.0000423 | 0.0000759 |
| *Veillonella nakazawae* | Lactoferrin | -0.000342 | 0.000503 | 0.500 | 0.662 | -0.0013291 | 0.0006443 |
| *Veillonella nakazawae* | Lysozyme | -0.004286 | 0.002466 | 0.090 | 0.193 | -0.0091187 | 0.0005463 |
| *Veillonella nakazawae* | sIgA | -0.003225 | 0.003137 | 0.310 | 0.499 | -0.0093731 | 0.0029234 |
| *Veillonella nakazawae* | Delivery mode: emergency caesarean | 0.860790 | 1.493752 | 0.568 | 0.706 | -2.0669642 | 3.7885447 |
| *Veillonella nakazawae* | Delivery mode: planned caesarean | -1.391030 | 1.493752 | 0.358 | 0.519 | -4.3187847 | 1.5367242 |
| *Veillonella nakazawae* | First week pacifier: yes | 1.000092 | 1.188370 | 0.405 | 0.570 | -1.3291133 | 3.3292967 |
| *Veillonella nakazawae* | Siblings: yes | -1.252225 | 1.201203 | 0.304 | 0.499 | -3.6065827 | 1.1021333 |
| *Veillonella nakazawae* | Pre-pregnancy BMI: high and obese | -1.243194 | 1.248446 | 0.326 | 0.505 | -3.6901479 | 1.2037599 |
| *Staphylococcus lugdunensis* | Ca | -0.000001 | 0.000005 | 0.836 | 0.940 | -0.0000107 | 0.0000087 |
| *Staphylococcus lugdunensis* | Na | -0.000002 | 0.000006 | 0.741 | 0.940 | -0.0000149 | 0.0000106 |
| *Staphylococcus lugdunensis* | K | 0.000000 | 0.000003 | 0.987 | 0.987 | -0.0000053 | 0.0000052 |
| *Staphylococcus lugdunensis* | P | -0.000001 | 0.000010 | 0.898 | 0.940 | -0.0000219 | 0.0000192 |
| *Staphylococcus lugdunensis* | Mg | -0.000011 | 0.000046 | 0.808 | 0.940 | -0.0001012 | 0.0000788 |
| *Staphylococcus lugdunensis* | I | 0.000006 | 0.000003 | 0.077 | 0.384 | -0.0000005 | 0.0000125 |
| *Staphylococcus lugdunensis* | Se | -0.000012 | 0.000064 | 0.851 | 0.940 | -0.0001385 | 0.0001141 |
| *Staphylococcus lugdunensis* | Cu | 0.000001 | 0.000004 | 0.782 | 0.940 | -0.0000072 | 0.0000095 |
| *Staphylococcus lugdunensis* | Mn | -0.000068 | 0.000371 | 0.855 | 0.940 | -0.0007956 | 0.0006587 |
| *Staphylococcus lugdunensis* | Mo | 0.000034 | 0.000169 | 0.842 | 0.940 | -0.0002981 | 0.0003663 |
| *Staphylococcus lugdunensis* | Fe | -0.000001 | 0.000003 | 0.752 | 0.940 | -0.0000061 | 0.0000044 |
| *Staphylococcus lugdunensis* | Time of milk sampling: Afternoon | -0.621468 | 0.862739 | 0.476 | 0.829 | -2.3124366 | 1.0695007 |
| *Staphylococcus lugdunensis* | Time of milk sampling: Night | -0.107836 | 0.829657 | 0.897 | 0.940 | -1.7339642 | 1.5182931 |
| *Staphylococcus lugdunensis* | Zn | 0.000001 | 0.000001 | 0.386 | 0.801 | -0.0000010 | 0.0000026 |
| *Staphylococcus lugdunensis* | Lactose | 0.000015 | 0.000019 | 0.450 | 0.829 | -0.0000233 | 0.0000531 |
| *Staphylococcus lugdunensis* | Lactoferrin | 0.000558 | 0.000316 | 0.085 | 0.384 | -0.0000616 | 0.0011777 |
| *Staphylococcus lugdunensis* | Lysozyme | 0.000988 | 0.001653 | 0.554 | 0.886 | -0.0022520 | 0.0042274 |
| *Staphylococcus lugdunensis* | sIgA | -0.001173 | 0.002054 | 0.571 | 0.886 | -0.0051992 | 0.0028524 |
| *Staphylococcus lugdunensis* | Delivery mode: emergency caesarean | 0.656670 | 0.974371 | 0.504 | 0.841 | -1.2530979 | 2.5664369 |
| *Staphylococcus lugdunensis* | Delivery mode: planned caesarean | 0.823172 | 0.974371 | 0.403 | 0.801 | -1.0865959 | 2.7329389 |
| *Staphylococcus lugdunensis* | First week pacifier: yes | 1.007540 | 0.719465 | 0.170 | 0.551 | -0.4026116 | 2.4176914 |
| *Staphylococcus lugdunensis* | Siblings: yes | 0.049675 | 0.790071 | 0.950 | 0.972 | -1.4988640 | 1.5982144 |
| *Staphylococcus lugdunensis* | Pre-pregnancy BMI: high and obese | -0.145467 | 0.818546 | 0.860 | 0.940 | -1.7498177 | 1.4588835 |
| *Haemophilus parainfluenzae* | Ca | -0.000004 | 0.000008 | 0.652 | 0.863 | -0.0000193 | 0.0000121 |
| *Haemophilus parainfluenzae* | Na | -0.000007 | 0.000011 | 0.533 | 0.812 | -0.0000272 | 0.0000140 |
| *Haemophilus parainfluenzae* | K | -0.000001 | 0.000004 | 0.821 | 0.940 | -0.0000095 | 0.0000075 |
| *Haemophilus parainfluenzae* | P | -0.000011 | 0.000017 | 0.525 | 0.812 | -0.0000440 | 0.0000223 |
| *Haemophilus parainfluenzae* | Mg | 0.000022 | 0.000075 | 0.770 | 0.917 | -0.0001241 | 0.0001681 |
| *Haemophilus parainfluenzae* | I | 0.000000 | 0.000006 | 0.950 | 0.981 | -0.0000106 | 0.0000113 |
| *Haemophilus parainfluenzae* | Se | -0.000003 | 0.000105 | 0.981 | 0.981 | -0.0002077 | 0.0002026 |
| *Haemophilus parainfluenzae* | Cu | -0.000005 | 0.000007 | 0.481 | 0.801 | -0.0000184 | 0.0000086 |
| *Haemophilus parainfluenzae* | Mn | -0.000775 | 0.000590 | 0.197 | 0.402 | -0.0019311 | 0.0003811 |
| *Haemophilus parainfluenzae* | Mo | -0.000161 | 0.000274 | 0.561 | 0.815 | -0.0006980 | 0.0003767 |
| *Haemophilus parainfluenzae* | Fe | -0.000001 | 0.000004 | 0.775 | 0.917 | -0.0000095 | 0.0000071 |
| *Haemophilus parainfluenzae* | Time of milk sampling: Afternoon | -0.112661 | 1.364530 | 0.935 | 0.981 | -2.7871412 | 2.5618183 |
| *Haemophilus parainfluenzae* | Time of milk sampling: Night | 1.944167 | 1.312208 | 0.147 | 0.354 | -0.6277597 | 4.5160945 |
| *Haemophilus parainfluenzae* | Zn | 0.000000 | 0.000001 | 0.962 | 0.981 | -0.0000030 | 0.0000028 |
| *Haemophilus parainfluenzae* | Lactose | -0.000011 | 0.000032 | 0.725 | 0.907 | -0.0000737 | 0.0000511 |
| *Haemophilus parainfluenzae* | Lactoferrin | -0.000709 | 0.000521 | 0.182 | 0.389 | -0.0017310 | 0.0003126 |
| *Haemophilus parainfluenzae* | Lysozyme | -0.001653 | 0.002683 | 0.542 | 0.812 | -0.0069122 | 0.0036070 |
| *Haemophilus parainfluenzae* | sIgA | 0.003418 | 0.003304 | 0.307 | 0.576 | -0.0030589 | 0.0098946 |
| *Haemophilus parainfluenzae* | Delivery mode: emergency caesarean | -1.303252 | 1.555106 | 0.407 | 0.711 | -4.3512594 | 1.7447559 |
| *Haemophilus parainfluenzae* | Delivery mode: planned caesarean | 1.760522 | 1.555106 | 0.265 | 0.518 | -1.2874856 | 4.8085297 |
| *Haemophilus parainfluenzae* | First week pacifier: yes | 0.621637 | 1.256669 | 0.624 | 0.850 | -1.8414329 | 3.0847078 |
| *Haemophilus parainfluenzae* | Siblings: yes | -0.267700 | 1.282376 | 0.836 | 0.940 | -2.7811572 | 2.2457579 |
| *Haemophilus parainfluenzae* | Pre-pregnancy BMI: high and obese | 0.554092 | 1.338689 | 0.681 | 0.876 | -2.0697374 | 3.1779216 |
| Unclassified *Flavobacteriaceae* | Ca | 0.000009 | 0.000009 | 0.344 | 0.615 | -0.0000092 | 0.0000268 |
| Unclassified *Flavobacteriaceae* | Na | -0.000002 | 0.000012 | 0.875 | 0.919 | -0.0000258 | 0.0000220 |
| Unclassified *Flavobacteriaceae* | K | -0.000001 | 0.000005 | 0.913 | 0.934 | -0.0000103 | 0.0000092 |
| Unclassified *Flavobacteriaceae* | P | -0.000014 | 0.000019 | 0.467 | 0.725 | -0.0000525 | 0.0000239 |
| Unclassified *Flavobacteriaceae* | Mg | 0.000038 | 0.000086 | 0.663 | 0.785 | -0.0001308 | 0.0002063 |
| Unclassified *Flavobacteriaceae* | I | -0.000004 | 0.000006 | 0.588 | 0.747 | -0.0000161 | 0.0000091 |
| Unclassified *Flavobacteriaceae* | Se | -0.000054 | 0.000121 | 0.658 | 0.785 | -0.0002902 | 0.0001826 |
| Unclassified *Flavobacteriaceae* | Cu | 0.000006 | 0.000008 | 0.462 | 0.725 | -0.0000097 | 0.0000215 |
| Unclassified *Flavobacteriaceae* | Mn | -0.000659 | 0.000688 | 0.344 | 0.615 | -0.0020077 | 0.0006902 |
| Unclassified *Flavobacteriaceae* | Mo | 0.000174 | 0.000317 | 0.586 | 0.747 | -0.0004471 | 0.0007950 |
| Unclassified *Flavobacteriaceae* | Fe | -0.000005 | 0.000005 | 0.351 | 0.615 | -0.0000145 | 0.0000051 |
| Unclassified *Flavobacteriaceae* | Time of milk sampling: Afternoon | 1.466036 | 1.599394 | 0.365 | 0.615 | -1.6687759 | 4.6008479 |
| Unclassified *Flavobacteriaceae* | Time of milk sampling: Night | -0.022073 | 1.538065 | 0.989 | 0.989 | -3.0366813 | 2.9925345 |
| Unclassified *Flavobacteriaceae* | Zn | -0.000002 | 0.000002 | 0.179 | 0.423 | -0.0000055 | 0.0000010 |
| Unclassified *Flavobacteriaceae* | Lactose | -0.000033 | 0.000036 | 0.369 | 0.615 | -0.0001046 | 0.0000383 |
| Unclassified *Flavobacteriaceae* | Lactoferrin | -0.000357 | 0.000614 | 0.564 | 0.747 | -0.0015601 | 0.0008455 |
| Unclassified *Flavobacteriaceae* | Lysozyme | -0.003339 | 0.003069 | 0.283 | 0.579 | -0.0093536 | 0.0026754 |
| Unclassified *Flavobacteriaceae* | sIgA | -0.006749 | 0.003715 | 0.077 | 0.231 | -0.0140312 | 0.0005323 |
| Unclassified *Flavobacteriaceae* | Delivery mode: emergency caesarean | 0.958906 | 1.801517 | 0.598 | 0.747 | -2.5720666 | 4.4898794 |
| Unclassified *Flavobacteriaceae* | Delivery mode: planned caesarean | -2.260498 | 1.801517 | 0.217 | 0.489 | -5.7914709 | 1.2704751 |
| Unclassified *Flavobacteriaceae* | First week pacifier: yes | -1.981226 | 1.424441 | 0.172 | 0.423 | -4.7731296 | 0.8106772 |
| Unclassified *Flavobacteriaceae* | Siblings: yes | -0.955336 | 1.474262 | 0.521 | 0.747 | -3.8448899 | 1.9342171 |
| Unclassified *Flavobacteriaceae* | Pre-pregnancy BMI: high and obese | 0.479965 | 1.544077 | 0.758 | 0.860 | -2.5464271 | 3.5063567 |
| *Streptococcus parasanguinis* | Ca | -0.000009 | 0.000008 | 0.300 | 0.434 | -0.0000244 | 0.0000074 |
| *Streptococcus parasanguinis* | Na | -0.000011 | 0.000011 | 0.308 | 0.434 | -0.0000319 | 0.0000099 |
| *Streptococcus parasanguinis* | K | -0.000004 | 0.000004 | 0.349 | 0.476 | -0.0000127 | 0.0000044 |
| *Streptococcus parasanguinis* | P | -0.000019 | 0.000017 | 0.271 | 0.434 | -0.0000526 | 0.0000145 |
| *Streptococcus parasanguinis* | Mg | -0.000038 | 0.000076 | 0.620 | 0.692 | -0.0001871 | 0.0001111 |
| *Streptococcus parasanguinis* | I | -0.000008 | 0.000006 | 0.151 | 0.272 | -0.0000190 | 0.0000028 |
| *Streptococcus parasanguinis* | Se | -0.000163 | 0.000104 | 0.124 | 0.242 | -0.0003668 | 0.0000402 |
| *Streptococcus parasanguinis* | Cu | -0.000007 | 0.000007 | 0.301 | 0.434 | -0.0000210 | 0.0000064 |
| *Streptococcus parasanguinis* | Mn | -0.000468 | 0.000612 | 0.449 | 0.578 | -0.0016669 | 0.0007319 |
| *Streptococcus parasanguinis* | Mo | -0.000112 | 0.000281 | 0.693 | 0.725 | -0.0006628 | 0.0004390 |
| *Streptococcus parasanguinis* | Fe | -0.000007 | 0.000004 | 0.091 | 0.186 | -0.0000160 | 0.0000010 |
| *Streptococcus parasanguinis* | Time of milk sampling: Afternoon | 0.295374 | 1.387780 | 0.833 | 0.833 | -2.4246743 | 3.0154227 |
| *Streptococcus parasanguinis* | Time of milk sampling: Night | -0.626443 | 1.334566 | 0.642 | 0.692 | -3.2421915 | 1.9893057 |
| *Streptococcus parasanguinis* | Zn | -0.000001 | 0.000001 | 0.437 | 0.578 | -0.0000041 | 0.0000018 |
| *Streptococcus parasanguinis* | Lactose | -0.000047 | 0.000032 | 0.145 | 0.272 | -0.0001094 | 0.0000150 |
| *Streptococcus parasanguinis* | Lactoferrin | -0.000759 | 0.000532 | 0.162 | 0.280 | -0.0018018 | 0.0002837 |
| *Streptococcus parasanguinis* | Lysozyme | -0.003352 | 0.002705 | 0.223 | 0.371 | -0.0086541 | 0.0019506 |
| *Streptococcus parasanguinis* | sIgA | 0.002370 | 0.003405 | 0.491 | 0.597 | -0.0043034 | 0.0090435 |
| *Streptococcus parasanguinis* | Delivery mode: emergency caesarean | -0.968644 | 1.626871 | 0.555 | 0.646 | -4.1573107 | 2.2200224 |
| *Streptococcus parasanguinis* | Delivery mode: planned caesarean | -0.957626 | 1.626871 | 0.560 | 0.646 | -4.1462928 | 2.2310403 |
| *Streptococcus parasanguinis* | First week pacifier: yes | 2.328769 | 1.212325 | 0.062 | 0.133 | -0.0473873 | 4.7049252 |
| *Streptococcus parasanguinis* | Siblings: yes | -0.327402 | 1.311325 | 0.804 | 0.822 | -2.8975989 | 2.2427951 |
| *Streptococcus parasanguinis* | Pre-pregnancy BMI: high and obese | -1.006705 | 1.361688 | 0.464 | 0.580 | -3.6756142 | 1.6622041 |
| *Bifidobacterium longum* | Ca | -0.000004 | 0.000005 | 0.444 | 0.540 | -0.0000139 | 0.0000061 |
| *Bifidobacterium longum* | Na | -0.000009 | 0.000007 | 0.179 | 0.415 | -0.0000220 | 0.0000039 |
| *Bifidobacterium longum* | K | -0.000003 | 0.000003 | 0.347 | 0.474 | -0.0000080 | 0.0000028 |
| *Bifidobacterium longum* | P | -0.000011 | 0.000011 | 0.314 | 0.456 | -0.0000320 | 0.0000101 |
| *Bifidobacterium longum* | Mg | -0.000058 | 0.000047 | 0.221 | 0.415 | -0.0001501 | 0.0000336 |
| *Bifidobacterium longum* | I | -0.000005 | 0.000003 | 0.178 | 0.415 | -0.0000116 | 0.0000020 |
| *Bifidobacterium longum* | Se | -0.000063 | 0.000066 | 0.344 | 0.474 | -0.0001933 | 0.0000664 |
| *Bifidobacterium longum* | Cu | -0.000001 | 0.000004 | 0.826 | 0.844 | -0.0000097 | 0.0000077 |
| *Bifidobacterium longum* | Mn | -0.000476 | 0.000378 | 0.216 | 0.415 | -0.0012170 | 0.0002658 |
| *Bifidobacterium longum* | Mo | -0.000190 | 0.000174 | 0.281 | 0.436 | -0.0005302 | 0.0001504 |
| *Bifidobacterium longum* | Fe | -0.000003 | 0.000003 | 0.292 | 0.439 | -0.0000083 | 0.0000024 |
| *Bifidobacterium longum* | Time of milk sampling: Afternoon | 0.212025 | 0.881946 | 0.811 | 0.844 | -1.5165892 | 1.9406390 |
| *Bifidobacterium longum* | Time of milk sampling: Night | -0.727495 | 0.848128 | 0.397 | 0.525 | -2.3898258 | 0.9348354 |
| *Bifidobacterium longum* | Zn | -0.000001 | 0.000001 | 0.238 | 0.428 | -0.0000029 | 0.0000007 |
| *Bifidobacterium longum* | Lactose | -0.000016 | 0.000020 | 0.436 | 0.540 | -0.0000556 | 0.0000238 |
| *Bifidobacterium longum* | Lactoferrin | -0.000427 | 0.000335 | 0.210 | 0.415 | -0.0010825 | 0.0002293 |
| *Bifidobacterium longum* | Lysozyme | -0.002106 | 0.001693 | 0.221 | 0.415 | -0.0054243 | 0.0012116 |
| *Bifidobacterium longum* | sIgA | -0.001347 | 0.002133 | 0.532 | 0.629 | -0.0055279 | 0.0028344 |
| *Bifidobacterium longum* | Delivery mode: emergency caesarean | 1.318426 | 1.002140 | 0.196 | 0.415 | -0.6457677 | 3.2826192 |
| *Bifidobacterium longum* | Delivery mode: planned caesarean | -0.106864 | 1.002140 | 0.916 | 0.916 | -2.0710579 | 1.8573290 |
| *Bifidobacterium longum* | First week pacifier: yes | 0.376133 | 0.794906 | 0.639 | 0.694 | -1.1818819 | 1.9341486 |
| *Bifidobacterium longum* | Siblings: yes | 0.670380 | 0.814312 | 0.415 | 0.534 | -0.9256714 | 2.2664305 |
| *Bifidobacterium longum* | Pre-pregnancy BMI: high and obese | 0.501889 | 0.853093 | 0.560 | 0.630 | -1.1701734 | 2.1739516 |
| *Lactobacillus gasseri* | Ca | 0.000003 | 0.000008 | 0.668 | 0.850 | -0.0000118 | 0.0000185 |
| *Lactobacillus gasseri* | Na | 0.000000 | 0.000010 | 0.998 | 0.998 | -0.0000200 | 0.0000199 |
| *Lactobacillus gasseri* | K | 0.000002 | 0.000004 | 0.573 | 0.850 | -0.0000058 | 0.0000105 |
| *Lactobacillus gasseri* | P | 0.000008 | 0.000016 | 0.639 | 0.850 | -0.0000243 | 0.0000397 |
| *Lactobacillus gasseri* | Mg | -0.000023 | 0.000072 | 0.750 | 0.899 | -0.0001639 | 0.0001177 |
| *Lactobacillus gasseri* | I | -0.000005 | 0.000005 | 0.404 | 0.726 | -0.0000150 | 0.0000060 |
| *Lactobacillus gasseri* | Se | -0.000008 | 0.000101 | 0.934 | 0.977 | -0.0002062 | 0.0001893 |
| *Lactobacillus gasseri* | Cu | 0.000007 | 0.000007 | 0.301 | 0.668 | -0.0000060 | 0.0000198 |
| *Lactobacillus gasseri* | Mn | -0.000164 | 0.000580 | 0.779 | 0.899 | -0.0013016 | 0.0009737 |
| *Lactobacillus gasseri* | Mo | -0.000274 | 0.000262 | 0.302 | 0.668 | -0.0007869 | 0.0002394 |
| *Lactobacillus gasseri* | Fe | -0.000002 | 0.000004 | 0.559 | 0.850 | -0.0000102 | 0.0000055 |
| *Lactobacillus gasseri* | Time of milk sampling: Afternoon | 2.737211 | 1.285304 | 0.040 | 0.195 | 0.2180157 | 5.2564059 |
| *Lactobacillus gasseri* | Time of milk sampling: Night | 0.808204 | 1.236019 | 0.517 | 0.850 | -1.6143928 | 3.2308009 |
| *Lactobacillus gasseri* | Zn | -0.000001 | 0.000001 | 0.386 | 0.726 | -0.0000040 | 0.0000015 |
| *Lactobacillus gasseri* | Lactose | -0.000014 | 0.000031 | 0.645 | 0.850 | -0.0000743 | 0.0000458 |
| *Lactobacillus gasseri* | Lactoferrin | -0.000510 | 0.000508 | 0.321 | 0.668 | -0.0015054 | 0.0004851 |
| *Lactobacillus gasseri* | Lysozyme | -0.002204 | 0.002575 | 0.397 | 0.726 | -0.0072517 | 0.0028433 |
| *Lactobacillus gasseri* | sIgA | -0.003865 | 0.003169 | 0.230 | 0.608 | -0.0100764 | 0.0023457 |
| *Lactobacillus gasseri* | Delivery mode: emergency caesarean | 1.513306 | 1.522677 | 0.327 | 0.668 | -1.4711407 | 4.4977524 |
| *Lactobacillus gasseri* | Delivery mode: planned caesarean | -0.320142 | 1.522677 | 0.835 | 0.925 | -3.3045884 | 2.6643046 |
| *Lactobacillus gasseri* | First week pacifier: yes | -0.504732 | 1.213976 | 0.680 | 0.850 | -2.8841246 | 1.8746603 |
| *Lactobacillus gasseri* | Siblings: yes | -0.191324 | 1.236481 | 0.878 | 0.941 | -2.6148273 | 2.2321794 |
| *Lactobacillus gasseri* | Pre-pregnancy BMI: high and obese | -0.398888 | 1.291290 | 0.759 | 0.899 | -2.9298169 | 2.1320411 |

| **Response variable** | **Explanatory variables** | **Estimate** | **Standard Error** | **P-value** | **BH corrected P-value** | **Lower CI** | **Upper CI** |
| --- | --- | --- | --- | --- | --- | --- | --- |
| Shannon diversity | Ca | -0.000003 | 0.000001 | 0.046 | 0.083 | -0.000005 | 0.000000 |
| Shannon diversity | Na | -0.000004 | 0.000002 | 0.021 | **0.040** | -0.000007 | -0.000001 |
| Shannon diversity | K | -0.000001 | 0.000001 | 0.065 | 0.112 | -0.000003 | 0.000000 |
| Shannon diversity | P | -0.000004 | 0.000003 | 0.122 | 0.181 | -0.000010 | 0.000001 |
| Shannon diversity | Mg | -0.000011 | 0.000012 | 0.368 | 0.440 | -0.000036 | 0.000013 |
| Shannon diversity | I | -0.000002 | 0.000001 | 0.008 | **0.015** | -0.000004 | -0.000001 |
| Shannon diversity | Se | -0.000014 | 0.000017 | 0.413 | 0.480 | -0.000048 | 0.000020 |
| Shannon diversity | Cu | -0.000001 | 0.000001 | 0.206 | 0.270 | -0.000004 | 0.000001 |
| Shannon diversity | Mn | -0.000126 | 0.000100 | 0.214 | 0.270 | -0.000321 | 0.000069 |
| Shannon diversity | Mo | -0.000016 | 0.000046 | 0.736 | 0.772 | -0.000106 | 0.000075 |
| Shannon diversity | Fe | -0.000001 | 0.000001 | 0.159 | 0.220 | -0.000002 | 0.000000 |
| Shannon diversity | Zn | 0.000000 | 0.000000 | 0.113 | 0.174 | -0.000001 | 0.000000 |
| Shannon diversity | Lactose | -0.000009 | 0.000005 | 0.104 | 0.165 | -0.000019 | 0.000002 |
| Shannon diversity | Lactoferrin | -0.000249 | 0.000080 | 0.003 | **0.007** | -0.000405 | -0.000093 |
| Shannon diversity | Lysozyme | -0.000083 | 0.000447 | 0.855 | 0.857 | -0.000960 | 0.000795 |
| Shannon diversity | sIgA | 0.000704 | 0.000551 | 0.209 | 0.270 | -0.000376 | 0.001783 |
| Shannon diversity | Delivery mode: planned caesarean | 0.141178 | 0.318494 | 0.660 | 0.709 | -0.483071 | 0.765426 |
| Shannon diversity | Delivery mode: emergency caesarean | 0.465767 | 0.256017 | 0.076 | 0.126 | -0.036027 | 0.967561 |
| Shannon diversity | First week pacifier: yes | 0.309211 | 0.203924 | 0.137 | 0.197 | -0.090481 | 0.708903 |
| Shannon diversity | Siblings: yes | -0.152468 | 0.206852 | 0.465 | 0.526 | -0.557898 | 0.252963 |
| Shannon diversity | Pre-pregnancy BMI: high and obese | 0.040188 | 0.221166 | 0.857 | 0.857 | -0.393296 | 0.473673 |
| Richness | Ca | -0.000429 | 0.000250 | 0.093 | 0.154 | -0.000919 | 0.000060 |
| Richness | Na | -0.000720 | 0.000322 | 0.031 | 0.060 | -0.001351 | -0.000089 |
| Richness | K | -0.000204 | 0.000136 | 0.139 | 0.207 | -0.000470 | 0.000061 |
| Richness | P | -0.000663 | 0.000534 | 0.221 | 0.297 | -0.001709 | 0.000382 |
| Richness | Mg | -0.002234 | 0.002390 | 0.355 | 0.449 | -0.006918 | 0.002450 |
| Richness | I | -0.000382 | 0.000168 | 0.028 | 0.057 | -0.000711 | -0.000054 |
| Richness | Se | -0.002196 | 0.003338 | 0.514 | 0.582 | -0.008738 | 0.004347 |
| Richness | Cu | -0.000202 | 0.000221 | 0.366 | 0.450 | -0.000635 | 0.000231 |
| Richness | Mn | -0.025444 | 0.019079 | 0.190 | 0.263 | -0.062839 | 0.011951 |
| Richness | Mo | -0.000738 | 0.008864 | 0.934 | 0.956 | -0.018110 | 0.016635 |
| Richness | Fe | -0.000185 | 0.000133 | 0.171 | 0.246 | -0.000445 | 0.000075 |
| Richness | Zn | -0.000082 | 0.000046 | 0.082 | 0.141 | -0.000172 | 0.000008 |
| Richness | Lactose | -0.001637 | 0.000999 | 0.109 | 0.174 | -0.003596 | 0.000322 |
| Richness | Lactoferrin | -0.033688 | 0.016153 | 0.043 | 0.081 | -0.065349 | -0.002028 |
| Richness | Lysozyme | -0.001605 | 0.085865 | 0.985 | 0.985 | -0.169901 | 0.166690 |
| Richness | sIgA | 0.109206 | 0.106383 | 0.311 | 0.405 | -0.099305 | 0.317717 |
| Richness | Delivery mode: planned caesarean | 5.319738 | 59.925537 | 0.930 | 0.956 | -112.134315 | 122.773791 |
| Richness | Delivery mode: emergency caesarean | 91.442933 | 48.170361 | 0.065 | 0.116 | -2.970974 | 185.856841 |
| Richness | First week pacifier: yes | 61.386880 | 39.210030 | 0.125 | 0.192 | -15.464778 | 138.238538 |
| Richness | Siblings: yes | -18.280673 | 39.835438 | 0.649 | 0.715 | -96.358133 | 59.796786 |
| Richness | Pre-pregnancy BMI: high and obese | 15.197000 | 42.282514 | 0.721 | 0.775 | -67.676728 | 98.070728 |
| *Streptococcus mitis* | Ca | 0.000005 | 0.000004 | 0.194 | 0.347 | -0.000002 | 0.000012 |
| *Streptococcus mitis* | Na | 0.000006 | 0.000005 | 0.228 | 0.350 | -0.000003 | 0.000015 |
| *Streptococcus mitis* | K | 0.000002 | 0.000002 | 0.348 | 0.482 | -0.000002 | 0.000006 |
| *Streptococcus mitis* | P | 0.000006 | 0.000008 | 0.438 | 0.588 | -0.000009 | 0.000021 |
| *Streptococcus mitis* | Mg | 0.000014 | 0.000034 | 0.680 | 0.770 | -0.000053 | 0.000081 |
| *Streptococcus mitis* | I | 0.000003 | 0.000002 | 0.183 | 0.343 | -0.000002 | 0.000008 |
| *Streptococcus mitis* | Se | 0.000030 | 0.000047 | 0.536 | 0.652 | -0.000063 | 0.000123 |
| *Streptococcus mitis* | Cu | 0.000000 | 0.000003 | 0.941 | 0.941 | -0.000006 | 0.000006 |
| *Streptococcus mitis* | Mn | 0.000198 | 0.000275 | 0.475 | 0.601 | -0.000341 | 0.000737 |
| *Streptococcus mitis* | Mo | 0.000094 | 0.000125 | 0.458 | 0.597 | -0.000151 | 0.000339 |
| *Streptococcus mitis* | Fe | 0.000003 | 0.000002 | 0.097 | 0.189 | 0.000000 | 0.000007 |
| *Streptococcus mitis* | Zn | 0.000001 | 0.000001 | 0.220 | 0.350 | 0.000000 | 0.000002 |
| *Streptococcus mitis* | Lactose | 0.000018 | 0.000014 | 0.210 | 0.347 | -0.000010 | 0.000046 |
| *Streptococcus mitis* | Lactoferrin | 0.000401 | 0.000233 | 0.093 | 0.189 | -0.000056 | 0.000857 |
| *Streptococcus mitis* | Lysozyme | -0.000739 | 0.001213 | 0.546 | 0.652 | -0.003117 | 0.001639 |
| *Streptococcus mitis* | sIgA | -0.000421 | 0.001528 | 0.784 | 0.843 | -0.003415 | 0.002574 |
| *Streptococcus mitis* | Delivery mode: planned caesarean | 0.148933 | 0.886734 | 0.867 | 0.910 | -1.589066 | 1.886932 |
| *Streptococcus mitis* | Delivery mode: emergency caesarean | -0.743517 | 0.712790 | 0.303 | 0.435 | -2.140585 | 0.653551 |
| *Streptococcus mitis* | First week pacifier: yes | -0.308147 | 0.573349 | 0.594 | 0.690 | -1.431911 | 0.815617 |
| *Streptococcus mitis* | Siblings: yes | 0.052695 | 0.566779 | 0.926 | 0.941 | -1.058191 | 1.163582 |
| *Streptococcus mitis* | Pre-pregnancy BMI: high and obese | -0.755782 | 0.589331 | 0.207 | 0.347 | -1.910871 | 0.399308 |
| *Gemella haemolysans* | Ca | -0.000008 | 0.000006 | 0.183 | 0.238 | -0.000019 | 0.000003 |
| *Gemella haemolysans* | Na | -0.000002 | 0.000008 | 0.776 | 0.794 | -0.000017 | 0.000013 |
| *Gemella haemolysans* | K | -0.000005 | 0.000003 | 0.091 | 0.139 | -0.000011 | 0.000001 |
| *Gemella haemolysans* | P | -0.000023 | 0.000012 | 0.052 | 0.089 | -0.000046 | -0.000001 |
| *Gemella haemolysans* | Mg | -0.000017 | 0.000054 | 0.763 | 0.794 | -0.000123 | 0.000090 |
| *Gemella haemolysans* | I | -0.000007 | 0.000004 | 0.094 | 0.140 | -0.000014 | 0.000001 |
| *Gemella haemolysans* | Se | -0.000102 | 0.000074 | 0.176 | 0.236 | -0.000247 | 0.000043 |
| *Gemella haemolysans* | Cu | -0.000011 | 0.000005 | 0.030 | 0.059 | -0.000020 | -0.000001 |
| *Gemella haemolysans* | Mn | -0.000524 | 0.000432 | 0.232 | 0.293 | -0.001370 | 0.000322 |
| *Gemella haemolysans* | Mo | 0.000110 | 0.000199 | 0.585 | 0.698 | -0.000281 | 0.000500 |
| *Gemella haemolysans* | Fe | -0.000002 | 0.000003 | 0.616 | 0.716 | -0.000008 | 0.000004 |
| *Gemella haemolysans* | Zn | -0.000002 | 0.000001 | 0.041 | 0.074 | -0.000004 | 0.000000 |
| *Gemella haemolysans* | Lactose | -0.000039 | 0.000022 | 0.089 | 0.139 | -0.000083 | 0.000005 |
| *Gemella haemolysans* | Lactoferrin | -0.000228 | 0.000381 | 0.554 | 0.681 | -0.000975 | 0.000520 |
| *Gemella haemolysans* | Lysozyme | 0.002693 | 0.001890 | 0.162 | 0.224 | -0.001011 | 0.006397 |
| *Gemella haemolysans* | sIgA | 0.003779 | 0.002356 | 0.116 | 0.167 | -0.000839 | 0.008398 |
| *Gemella haemolysans* | Delivery mode: planned caesarean | 2.788978 | 1.321881 | 0.041 | 0.074 | 0.198091 | 5.379864 |
| *Gemella haemolysans* | Delivery mode: emergency caesarean | 3.029763 | 1.062577 | 0.007 | **0.014** | 0.947113 | 5.112413 |
| *Gemella haemolysans* | First week pacifier: yes | 0.296896 | 0.912591 | 0.747 | 0.794 | -1.491783 | 2.085575 |
| *Gemella haemolysans* | Siblings: yes | 1.644926 | 0.863009 | 0.064 | 0.105 | -0.046572 | 3.336423 |
| *Gemella haemolysans* | Pre-pregnancy BMI: high and obese | -0.124190 | 0.961053 | 0.898 | 0.898 | -2.007855 | 1.759474 |
| *Rothia mucilaginosa* | Ca | -0.000005 | 0.000005 | 0.345 | 0.436 | -0.000016 | 0.000005 |
| *Rothia mucilaginosa* | Na | -0.000015 | 0.000007 | 0.028 | 0.053 | -0.000028 | -0.000002 |
| *Rothia mucilaginosa* | K | -0.000002 | 0.000003 | 0.425 | 0.481 | -0.000008 | 0.000003 |
| *Rothia mucilaginosa* | P | -0.000012 | 0.000011 | 0.296 | 0.411 | -0.000034 | 0.000010 |
| *Rothia mucilaginosa* | Mg | -0.000046 | 0.000050 | 0.356 | 0.438 | -0.000144 | 0.000051 |
| *Rothia mucilaginosa* | I | 0.000003 | 0.000004 | 0.484 | 0.534 | -0.000005 | 0.000010 |
| *Rothia mucilaginosa* | Se | -0.000060 | 0.000069 | 0.390 | 0.454 | -0.000196 | 0.000076 |
| *Rothia mucilaginosa* | Cu | -0.000002 | 0.000005 | 0.736 | 0.772 | -0.000011 | 0.000008 |
| *Rothia mucilaginosa* | Mn | -0.000791 | 0.000387 | 0.047 | 0.085 | -0.001548 | -0.000033 |
| *Rothia mucilaginosa* | Mo | -0.000273 | 0.000180 | 0.136 | 0.195 | -0.000625 | 0.000079 |
| *Rothia mucilaginosa* | Fe | -0.000006 | 0.000003 | 0.022 | **0.045** | -0.000012 | -0.000001 |
| *Rothia mucilaginosa* | Zn | 0.000000 | 0.000001 | 0.975 | 0.975 | -0.000002 | 0.000002 |
| *Rothia mucilaginosa* | Lactose | 0.000007 | 0.000021 | 0.762 | 0.781 | -0.000036 | 0.000049 |
| *Rothia mucilaginosa* | Lactoferrin | -0.000352 | 0.000350 | 0.320 | 0.430 | -0.001037 | 0.000333 |
| *Rothia mucilaginosa* | Lysozyme | -0.003918 | 0.001680 | 0.025 | **0.048** | -0.007212 | -0.000625 |
| *Rothia mucilaginosa* | sIgA | -0.004187 | 0.002147 | 0.058 | 0.092 | -0.008394 | 0.000021 |
| *Rothia mucilaginosa* | Delivery mode: planned caesarean | -0.444565 | 1.274921 | 0.729 | 0.772 | -2.943410 | 2.054280 |
| *Rothia mucilaginosa* | Delivery mode: emergency caesarean | -1.780558 | 1.024829 | 0.090 | 0.138 | -3.789222 | 0.228106 |
| *Rothia mucilaginosa* | First week pacifier: yes | 1.296261 | 0.814485 | 0.119 | 0.177 | -0.300128 | 2.892651 |
| *Rothia mucilaginosa* | Siblings: yes | -1.565769 | 0.795078 | 0.056 | 0.092 | -3.124122 | -0.007416 |
| *Rothia mucilaginosa* | Pre-pregnancy BMI: high and obese | 0.829681 | 0.847965 | 0.334 | 0.435 | -0.832330 | 2.491692 |
| *Streptococcus salivarius* group 1 | Ca | -0.000001 | 0.000009 | 0.905 | 0.966 | -0.000018 | 0.000016 |
| *Streptococcus salivarius* group 1 | Na | -0.000016 | 0.000011 | 0.171 | 0.319 | -0.000038 | 0.000006 |
| *Streptococcus salivarius* group 1 | K | -0.000002 | 0.000005 | 0.687 | 0.778 | -0.000011 | 0.000007 |
| *Streptococcus salivarius* group 1 | P | -0.000009 | 0.000018 | 0.615 | 0.766 | -0.000046 | 0.000027 |
| *Streptococcus salivarius* group 1 | Mg | -0.000077 | 0.000082 | 0.349 | 0.536 | -0.000237 | 0.000083 |
| *Streptococcus salivarius* group 1 | I | -0.000005 | 0.000006 | 0.375 | 0.537 | -0.000017 | 0.000006 |
| *Streptococcus salivarius* group 1 | Se | -0.000104 | 0.000113 | 0.363 | 0.537 | -0.000326 | 0.000118 |
| *Streptococcus salivarius* group 1 | Cu | 0.000000 | 0.000008 | 0.996 | 0.996 | -0.000015 | 0.000015 |
| *Streptococcus salivarius* group 1 | Mn | -0.001072 | 0.000644 | 0.103 | 0.212 | -0.002334 | 0.000190 |
| *Streptococcus salivarius* group 1 | Mo | -0.000015 | 0.000303 | 0.960 | 0.983 | -0.000608 | 0.000578 |
| *Streptococcus salivarius* group 1 | Fe | -0.000005 | 0.000005 | 0.240 | 0.414 | -0.000014 | 0.000004 |
| *Streptococcus salivarius* group 1 | Zn | -0.000002 | 0.000002 | 0.150 | 0.293 | -0.000005 | 0.000001 |
| *Streptococcus salivarius* group 1 | Lactose | -0.000014 | 0.000035 | 0.685 | 0.778 | -0.000083 | 0.000055 |
| *Streptococcus salivarius* group 1 | Lactoferrin | -0.000322 | 0.000578 | 0.580 | 0.763 | -0.001455 | 0.000810 |
| *Streptococcus salivarius* group 1 | Lysozyme | -0.003481 | 0.002880 | 0.234 | 0.414 | -0.009125 | 0.002164 |
| *Streptococcus salivarius* group 1 | sIgA | -0.003866 | 0.003627 | 0.293 | 0.466 | -0.010976 | 0.003244 |
| *Streptococcus salivarius* group 1 | Delivery mode: planned caesarean | -0.923834 | 2.189671 | 0.675 | 0.778 | -5.215589 | 3.367921 |
| *Streptococcus salivarius* group 1 | Delivery mode: emergency caesarean | -0.870322 | 1.760138 | 0.624 | 0.766 | -4.320193 | 2.579550 |
| *Streptococcus salivarius* group 1 | First week pacifier: yes | 1.479737 | 1.363082 | 0.284 | 0.466 | -1.191904 | 4.151378 |
| *Streptococcus salivarius* group 1 | Siblings: yes | 0.747008 | 1.358126 | 0.585 | 0.763 | -1.914919 | 3.408935 |
| *Streptococcus salivarius* group 1 | Pre-pregnancy BMI: high and obese | 0.144380 | 1.447070 | 0.921 | 0.966 | -2.691876 | 2.980637 |
| *Veillonella* sp. | Ca | -0.000010 | 0.000009 | 0.260 | 0.326 | -0.000027 | 0.000007 |
| *Veillonella* sp. | Na | -0.000009 | 0.000012 | 0.437 | 0.470 | -0.000032 | 0.000014 |
| *Veillonella* sp. | K | -0.000008 | 0.000005 | 0.101 | 0.161 | -0.000017 | 0.000001 |
| *Veillonella* sp. | P | -0.000034 | 0.000018 | 0.064 | 0.110 | -0.000069 | 0.000001 |
| *Veillonella* sp. | Mg | -0.000025 | 0.000083 | 0.761 | 0.761 | -0.000188 | 0.000137 |
| *Veillonella* sp. | I | -0.000017 | 0.000006 | 0.005 | **0.010** | -0.000027 | -0.000006 |
| *Veillonella* sp. | Se | -0.000192 | 0.000112 | 0.092 | 0.152 | -0.000411 | 0.000026 |
| *Veillonella* sp. | Cu | -0.000012 | 0.000007 | 0.126 | 0.186 | -0.000026 | 0.000003 |
| *Veillonella* sp. | Mn | -0.000739 | 0.000661 | 0.270 | 0.326 | -0.002034 | 0.000556 |
| *Veillonella* sp. | Mo | -0.000488 | 0.000295 | 0.106 | 0.163 | -0.001068 | 0.000091 |
| *Veillonella* sp. | Fe | -0.000009 | 0.000004 | 0.053 | 0.099 | -0.000018 | 0.000000 |
| *Veillonella* sp. | Zn | -0.000001 | 0.000002 | 0.618 | 0.642 | -0.000004 | 0.000002 |
| *Veillonella* sp. | Lactose | -0.000039 | 0.000035 | 0.275 | 0.326 | -0.000107 | 0.000030 |
| *Veillonella* sp. | Lactoferrin | -0.000758 | 0.000573 | 0.193 | 0.267 | -0.001881 | 0.000364 |
| *Veillonella* sp. | Lysozyme | -0.006713 | 0.002764 | 0.020 | **0.040** | -0.012131 | -0.001296 |
| *Veillonella* sp. | sIgA | -0.003995 | 0.003656 | 0.281 | 0.326 | -0.011162 | 0.003171 |
| *Veillonella* sp. | Delivery mode: planned caesarean | 2.718991 | 2.114934 | 0.206 | 0.277 | -1.426279 | 6.864262 |
| *Veillonella* sp. | Delivery mode: emergency caesarean | 3.358128 | 1.700062 | 0.055 | 0.099 | 0.026006 | 6.690250 |
| *Veillonella* sp. | First week pacifier: yes | -1.123534 | 1.346165 | 0.409 | 0.451 | -3.762018 | 1.514950 |
| *Veillonella* sp. | Siblings: yes | -1.676186 | 1.349761 | 0.221 | 0.288 | -4.321717 | 0.969345 |
| *Veillonella* sp. | Pre-pregnancy BMI: high and obese | 1.327488 | 1.454870 | 0.367 | 0.415 | -1.524056 | 4.179033 |
| *Neisseria subflava* | Ca | 0.000003 | 0.000007 | 0.655 | 0.818 | -0.000011 | 0.000017 |
| *Neisseria subflava* | Na | 0.000007 | 0.000009 | 0.465 | 0.725 | -0.000011 | 0.000025 |
| *Neisseria subflava* | K | 0.000004 | 0.000004 | 0.256 | 0.550 | -0.000003 | 0.000012 |
| *Neisseria subflava* | P | 0.000019 | 0.000015 | 0.203 | 0.514 | -0.000010 | 0.000048 |
| *Neisseria subflava* | Mg | 0.000111 | 0.000065 | 0.095 | 0.396 | -0.000016 | 0.000238 |
| *Neisseria subflava* | I | 0.000002 | 0.000005 | 0.685 | 0.818 | -0.000008 | 0.000012 |
| *Neisseria subflava* | Se | 0.000089 | 0.000092 | 0.338 | 0.632 | -0.000091 | 0.000270 |
| *Neisseria subflava* | Cu | 0.000002 | 0.000006 | 0.776 | 0.853 | -0.000010 | 0.000014 |
| *Neisseria subflava* | Mn | 0.000916 | 0.000522 | 0.086 | 0.396 | -0.000106 | 0.001939 |
| *Neisseria subflava* | Mo | 0.000285 | 0.000242 | 0.246 | 0.550 | -0.000190 | 0.000759 |
| *Neisseria subflava* | Fe | 0.000007 | 0.000004 | 0.079 | 0.396 | -0.000001 | 0.000014 |
| *Neisseria subflava* | Zn | 0.000000 | 0.000001 | 0.814 | 0.853 | -0.000002 | 0.000003 |
| *Neisseria subflava* | Lactose | 0.000026 | 0.000028 | 0.358 | 0.642 | -0.000029 | 0.000082 |
| *Neisseria subflava* | Lactoferrin | -0.000062 | 0.000471 | 0.896 | 0.917 | -0.000986 | 0.000862 |
| *Neisseria subflava* | Lysozyme | 0.001121 | 0.002377 | 0.640 | 0.818 | -0.003537 | 0.005779 |
| *Neisseria subflava* | sIgA | 0.001735 | 0.002978 | 0.563 | 0.793 | -0.004101 | 0.007572 |
| *Neisseria subflava* | Delivery mode: planned caesarean | 2.273909 | 1.748562 | 0.201 | 0.514 | -1.153273 | 5.701091 |
| *Neisseria subflava* | Delivery mode: emergency caesarean | 1.464380 | 1.405559 | 0.304 | 0.594 | -1.290516 | 4.219276 |
| *Neisseria subflava* | First week pacifier: yes | 0.284410 | 1.120584 | 0.801 | 0.853 | -1.911934 | 2.480754 |
| *Neisseria subflava* | Siblings: yes | 0.799126 | 1.101366 | 0.472 | 0.725 | -1.359552 | 2.957803 |
| *Neisseria subflava* | Pre-pregnancy BMI: high and obese | 2.033834 | 1.141190 | 0.082 | 0.396 | -0.202898 | 4.270566 |
| *Streptococcus oralis* | Ca | 0.000012 | 0.000009 | 0.187 | 0.310 | -0.000006 | 0.000030 |
| *Streptococcus oralis* | Na | -0.000008 | 0.000012 | 0.520 | 0.670 | -0.000032 | 0.000016 |
| *Streptococcus oralis* | K | 0.000005 | 0.000005 | 0.320 | 0.474 | -0.000005 | 0.000015 |
| *Streptococcus oralis* | P | 0.000029 | 0.000019 | 0.132 | 0.284 | -0.000008 | 0.000066 |
| *Streptococcus oralis* | Mg | 0.000052 | 0.000086 | 0.553 | 0.670 | -0.000118 | 0.000221 |
| *Streptococcus oralis* | I | 0.000003 | 0.000006 | 0.592 | 0.670 | -0.000009 | 0.000016 |
| *Streptococcus oralis* | Se | 0.000059 | 0.000120 | 0.624 | 0.688 | -0.000176 | 0.000295 |
| *Streptococcus oralis* | Cu | 0.000012 | 0.000008 | 0.148 | 0.302 | -0.000004 | 0.000027 |
| *Streptococcus oralis* | Mn | 0.000316 | 0.000698 | 0.653 | 0.702 | -0.001052 | 0.001685 |
| *Streptococcus oralis* | Mo | -0.000240 | 0.000316 | 0.452 | 0.608 | -0.000860 | 0.000380 |
| *Streptococcus oralis* | Fe | 0.000000 | 0.000005 | 0.922 | 0.944 | -0.000010 | 0.000009 |
| *Streptococcus oralis* | Zn | 0.000004 | 0.000002 | 0.013 | **0.039** | 0.000001 | 0.000007 |
| *Streptococcus oralis* | Lactose | 0.000086 | 0.000035 | 0.017 | **0.049** | 0.000018 | 0.000153 |
| *Streptococcus oralis* | Lactoferrin | 0.000010 | 0.000610 | 0.987 | 0.987 | -0.001186 | 0.001206 |
| *Streptococcus oralis* | Lysozyme | -0.001679 | 0.003073 | 0.588 | 0.670 | -0.007701 | 0.004344 |
| *Streptococcus oralis* | sIgA | -0.004907 | 0.003793 | 0.203 | 0.323 | -0.012341 | 0.002527 |
| *Streptococcus oralis* | Delivery mode: planned caesarean | 0.239860 | 2.268910 | 0.916 | 0.944 | -4.207203 | 4.686923 |
| *Streptococcus oralis* | Delivery mode: emergency caesarean | -1.533804 | 1.823834 | 0.405 | 0.581 | -5.108518 | 2.040909 |
| *Streptococcus oralis* | First week pacifier: yes | -2.181392 | 1.375085 | 0.121 | 0.273 | -4.876559 | 0.513775 |
| *Streptococcus oralis* | Siblings: yes | -1.909019 | 1.403059 | 0.181 | 0.310 | -4.659015 | 0.840977 |
| *Streptococcus oralis* | Pre-pregnancy BMI: high and obese | -0.929146 | 1.507961 | 0.541 | 0.670 | -3.884749 | 2.026457 |
| *Haemophilus haemolyticus* | Ca | -0.000003 | 0.000010 | 0.766 | 0.915 | -0.000023 | 0.000017 |
| *Haemophilus haemolyticus* | Na | 0.000001 | 0.000013 | 0.948 | 0.970 | -0.000025 | 0.000027 |
| *Haemophilus haemolyticus* | K | 0.000001 | 0.000005 | 0.864 | 0.953 | -0.000010 | 0.000011 |
| *Haemophilus haemolyticus* | P | 0.000022 | 0.000021 | 0.296 | 0.489 | -0.000019 | 0.000063 |
| *Haemophilus haemolyticus* | Mg | 0.000062 | 0.000093 | 0.506 | 0.806 | -0.000120 | 0.000244 |
| *Haemophilus haemolyticus* | I | -0.000003 | 0.000007 | 0.707 | 0.896 | -0.000016 | 0.000011 |
| *Haemophilus haemolyticus* | Se | 0.000190 | 0.000126 | 0.139 | 0.249 | -0.000057 | 0.000438 |
| *Haemophilus haemolyticus* | Cu | 0.000000 | 0.000009 | 0.968 | 0.970 | -0.000017 | 0.000017 |
| *Haemophilus haemolyticus* | Mn | 0.001524 | 0.000714 | 0.039 | 0.093 | 0.000124 | 0.002924 |
| *Haemophilus haemolyticus* | Mo | -0.000077 | 0.000342 | 0.824 | 0.953 | -0.000747 | 0.000594 |
| *Haemophilus haemolyticus* | Fe | 0.000008 | 0.000005 | 0.116 | 0.216 | -0.000002 | 0.000018 |
| *Haemophilus haemolyticus* | Zn | 0.000001 | 0.000002 | 0.671 | 0.896 | -0.000003 | 0.000004 |
| *Haemophilus haemolyticus* | Lactose | 0.000008 | 0.000040 | 0.851 | 0.953 | -0.000071 | 0.000086 |
| *Haemophilus haemolyticus* | Lactoferrin | 0.000025 | 0.000656 | 0.970 | 0.970 | -0.001261 | 0.001311 |
| *Haemophilus haemolyticus* | Lysozyme | 0.005620 | 0.003198 | 0.086 | 0.178 | -0.000649 | 0.011889 |
| *Haemophilus haemolyticus* | sIgA | 0.006524 | 0.004035 | 0.114 | 0.216 | -0.001385 | 0.014433 |
| *Haemophilus haemolyticus* | Delivery mode: planned caesarean | 0.947190 | 2.479156 | 0.704 | 0.896 | -3.911955 | 5.806336 |
| *Haemophilus haemolyticus* | Delivery mode: emergency caesarean | 0.924694 | 1.992837 | 0.645 | 0.896 | -2.981266 | 4.830655 |
| *Haemophilus haemolyticus* | First week pacifier: yes | 0.545136 | 1.562617 | 0.729 | 0.896 | -2.517594 | 3.607866 |
| *Haemophilus haemolyticus* | Siblings: yes | 0.577759 | 1.540029 | 0.709 | 0.896 | -2.440698 | 3.596217 |
| *Haemophilus haemolyticus* | Pre-pregnancy BMI: high and obese | 0.195343 | 1.646629 | 0.906 | 0.970 | -3.032050 | 3.422736 |
| *Veillonella nakazawae* | Ca | 0.000003 | 0.000008 | 0.708 | 0.801 | -0.000012 | 0.000018 |
| *Veillonella nakazawae* | Na | -0.000013 | 0.000010 | 0.192 | 0.342 | -0.000032 | 0.000006 |
| *Veillonella nakazawae* | K | 0.000000 | 0.000004 | 0.995 | 0.995 | -0.000008 | 0.000008 |
| *Veillonella nakazawae* | P | -0.000006 | 0.000016 | 0.689 | 0.801 | -0.000038 | 0.000025 |
| *Veillonella nakazawae* | Mg | -0.000042 | 0.000071 | 0.552 | 0.720 | -0.000181 | 0.000096 |
| *Veillonella nakazawae* | I | -0.000001 | 0.000005 | 0.784 | 0.842 | -0.000012 | 0.000009 |
| *Veillonella nakazawae* | Se | -0.000137 | 0.000096 | 0.164 | 0.307 | -0.000326 | 0.000053 |
| *Veillonella nakazawae* | Cu | 0.000002 | 0.000007 | 0.706 | 0.801 | -0.000010 | 0.000015 |
| *Veillonella nakazawae* | Mn | -0.000499 | 0.000568 | 0.385 | 0.551 | -0.001613 | 0.000614 |
| *Veillonella nakazawae* | Mo | 0.000018 | 0.000261 | 0.946 | 0.992 | -0.000494 | 0.000529 |
| *Veillonella nakazawae* | Fe | -0.000006 | 0.000004 | 0.131 | 0.255 | -0.000014 | 0.000002 |
| *Veillonella nakazawae* | Zn | 0.000000 | 0.000001 | 0.976 | 0.995 | -0.000003 | 0.000003 |
| *Veillonella nakazawae* | Lactose | 0.000016 | 0.000030 | 0.591 | 0.748 | -0.000043 | 0.000076 |
| *Veillonella nakazawae* | Lactoferrin | -0.000395 | 0.000496 | 0.431 | 0.598 | -0.001367 | 0.000578 |
| *Veillonella nakazawae* | Lysozyme | -0.004655 | 0.002421 | 0.061 | 0.126 | -0.009400 | 0.000089 |
| *Veillonella nakazawae* | sIgA | -0.003400 | 0.003127 | 0.283 | 0.435 | -0.009528 | 0.002728 |
| *Veillonella nakazawae* | Delivery mode: planned caesarean | -1.717363 | 1.874118 | 0.365 | 0.541 | -5.390635 | 1.955909 |
| *Veillonella nakazawae* | Delivery mode: emergency caesarean | -0.707741 | 1.506486 | 0.641 | 0.788 | -3.660452 | 2.244971 |
| *Veillonella nakazawae* | First week pacifier: yes | 0.755290 | 1.186798 | 0.528 | 0.710 | -1.570834 | 3.081414 |
| *Veillonella nakazawae* | Siblings: yes | -1.505202 | 1.151865 | 0.199 | 0.342 | -3.762858 | 0.752454 |
| *Veillonella nakazawae* | Pre-pregnancy BMI: high and obese | -1.505857 | 1.221977 | 0.225 | 0.358 | -3.900933 | 0.889219 |
| *Staphylococcus lugdunensis* | Ca | -0.000001 | 0.000005 | 0.860 | 0.978 | -0.000010 | 0.000009 |
| *Staphylococcus lugdunensis* | Na | -0.000002 | 0.000006 | 0.765 | 0.978 | -0.000014 | 0.000011 |
| *Staphylococcus lugdunensis* | K | 0.000000 | 0.000003 | 0.965 | 0.978 | -0.000005 | 0.000005 |
| *Staphylococcus lugdunensis* | P | 0.000000 | 0.000010 | 0.978 | 0.978 | -0.000020 | 0.000020 |
| *Staphylococcus lugdunensis* | Mg | -0.000010 | 0.000045 | 0.821 | 0.978 | -0.000098 | 0.000078 |
| *Staphylococcus lugdunensis* | I | 0.000006 | 0.000003 | 0.091 | 0.437 | -0.000001 | 0.000012 |
| *Staphylococcus lugdunensis* | Se | -0.000006 | 0.000063 | 0.920 | 0.978 | -0.000129 | 0.000116 |
| *Staphylococcus lugdunensis* | Cu | 0.000001 | 0.000004 | 0.784 | 0.978 | -0.000007 | 0.000009 |
| *Staphylococcus lugdunensis* | Mn | -0.000055 | 0.000363 | 0.880 | 0.978 | -0.000767 | 0.000657 |
| *Staphylococcus lugdunensis* | Mo | 0.000045 | 0.000165 | 0.785 | 0.978 | -0.000278 | 0.000369 |
| *Staphylococcus lugdunensis* | Fe | -0.000001 | 0.000003 | 0.731 | 0.978 | -0.000006 | 0.000004 |
| *Staphylococcus lugdunensis* | Zn | 0.000001 | 0.000001 | 0.376 | 0.781 | -0.000001 | 0.000003 |
| *Staphylococcus lugdunensis* | Lactose | 0.000015 | 0.000019 | 0.444 | 0.796 | -0.000023 | 0.000052 |
| *Staphylococcus lugdunensis* | Lactoferrin | 0.000566 | 0.000304 | 0.070 | 0.437 | -0.000030 | 0.001162 |
| *Staphylococcus lugdunensis* | Lysozyme | 0.001125 | 0.001591 | 0.483 | 0.799 | -0.001993 | 0.004243 |
| *Staphylococcus lugdunensis* | sIgA | -0.001145 | 0.002000 | 0.570 | 0.908 | -0.005065 | 0.002776 |
| *Staphylococcus lugdunensis* | Delivery mode: planned caesarean | -0.062858 | 1.187249 | 0.958 | 0.978 | -2.389865 | 2.264150 |
| *Staphylococcus lugdunensis* | Delivery mode: emergency caesarean | -0.682799 | 0.954354 | 0.478 | 0.799 | -2.553333 | 1.187736 |
| *Staphylococcus lugdunensis* | First week pacifier: yes | 1.047193 | 0.696563 | 0.141 | 0.504 | -0.318071 | 2.412456 |
| *Staphylococcus lugdunensis* | Siblings: yes | 0.105615 | 0.744235 | 0.888 | 0.978 | -1.353086 | 1.564315 |
| *Staphylococcus lugdunensis* | Pre-pregnancy BMI: high and obese | -0.064424 | 0.787979 | 0.935 | 0.978 | -1.608862 | 1.480014 |
| *Haemophilus parainfluenzae* | Ca | -0.000003 | 0.000008 | 0.677 | 0.874 | -0.000019 | 0.000012 |
| *Haemophilus parainfluenzae* | Na | -0.000006 | 0.000010 | 0.555 | 0.855 | -0.000026 | 0.000014 |
| *Haemophilus parainfluenzae* | K | -0.000001 | 0.000004 | 0.874 | 0.964 | -0.000009 | 0.000008 |
| *Haemophilus parainfluenzae* | P | -0.000009 | 0.000016 | 0.605 | 0.855 | -0.000041 | 0.000024 |
| *Haemophilus parainfluenzae* | Mg | 0.000024 | 0.000073 | 0.747 | 0.874 | -0.000120 | 0.000167 |
| *Haemophilus parainfluenzae* | I | 0.000000 | 0.000005 | 0.972 | 0.972 | -0.000011 | 0.000010 |
| *Haemophilus parainfluenzae* | Se | 0.000008 | 0.000102 | 0.935 | 0.972 | -0.000191 | 0.000208 |
| *Haemophilus parainfluenzae* | Cu | -0.000005 | 0.000007 | 0.469 | 0.807 | -0.000018 | 0.000008 |
| *Haemophilus parainfluenzae* | Mn | -0.000747 | 0.000579 | 0.204 | 0.440 | -0.001883 | 0.000389 |
| *Haemophilus parainfluenzae* | Mo | -0.000136 | 0.000268 | 0.614 | 0.855 | -0.000661 | 0.000389 |
| *Haemophilus parainfluenzae* | Fe | -0.000001 | 0.000004 | 0.752 | 0.874 | -0.000009 | 0.000007 |
| *Haemophilus parainfluenzae* | Zn | 0.000000 | 0.000001 | 0.965 | 0.972 | -0.000003 | 0.000003 |
| *Haemophilus parainfluenzae* | Lactose | -0.000011 | 0.000031 | 0.716 | 0.874 | -0.000073 | 0.000050 |
| *Haemophilus parainfluenzae* | Lactoferrin | -0.000634 | 0.000506 | 0.217 | 0.444 | -0.001626 | 0.000357 |
| *Haemophilus parainfluenzae* | Lysozyme | -0.001269 | 0.002597 | 0.628 | 0.855 | -0.006359 | 0.003821 |
| *Haemophilus parainfluenzae* | sIgA | 0.003396 | 0.003225 | 0.298 | 0.583 | -0.002924 | 0.009717 |
| *Haemophilus parainfluenzae* | Delivery mode: planned caesarean | 2.547230 | 1.909907 | 0.190 | 0.440 | -1.196187 | 6.290648 |
| *Haemophilus parainfluenzae* | Delivery mode: emergency caesarean | 1.241358 | 1.535254 | 0.424 | 0.792 | -1.767739 | 4.250456 |
| *Haemophilus parainfluenzae* | First week pacifier: yes | 0.737777 | 1.221600 | 0.549 | 0.855 | -1.656559 | 3.132113 |
| *Haemophilus parainfluenzae* | Siblings: yes | -0.132443 | 1.211207 | 0.913 | 0.972 | -2.506409 | 2.241522 |
| *Haemophilus parainfluenzae* | Pre-pregnancy BMI: high and obese | 0.711325 | 1.290777 | 0.585 | 0.855 | -1.818598 | 3.241248 |
| Unclassified *Flavobacteriaceae* | Ca | 0.000009 | 0.000009 | 0.319 | 0.615 | -0.000008 | 0.000027 |
| Unclassified *Flavobacteriaceae* | Na | -0.000001 | 0.000012 | 0.905 | 0.950 | -0.000025 | 0.000022 |
| Unclassified *Flavobacteriaceae* | K | 0.000000 | 0.000005 | 0.965 | 0.965 | -0.000010 | 0.000009 |
| Unclassified *Flavobacteriaceae* | P | -0.000012 | 0.000019 | 0.536 | 0.745 | -0.000049 | 0.000025 |
| Unclassified *Flavobacteriaceae* | Mg | 0.000040 | 0.000084 | 0.640 | 0.763 | -0.000126 | 0.000205 |
| Unclassified *Flavobacteriaceae* | I | -0.000004 | 0.000006 | 0.524 | 0.745 | -0.000016 | 0.000008 |
| Unclassified *Flavobacteriaceae* | Se | -0.000041 | 0.000117 | 0.728 | 0.824 | -0.000271 | 0.000189 |
| Unclassified *Flavobacteriaceae* | Cu | 0.000006 | 0.000008 | 0.460 | 0.745 | -0.000009 | 0.000021 |
| Unclassified *Flavobacteriaceae* | Mn | -0.000629 | 0.000675 | 0.357 | 0.615 | -0.001953 | 0.000694 |
| Unclassified *Flavobacteriaceae* | Mo | 0.000196 | 0.000309 | 0.529 | 0.745 | -0.000409 | 0.000801 |
| Unclassified *Flavobacteriaceae* | Fe | -0.000003 | 0.000005 | 0.471 | 0.745 | -0.000013 | 0.000006 |
| Unclassified *Flavobacteriaceae* | Zn | -0.000002 | 0.000002 | 0.173 | 0.392 | -0.000005 | 0.000001 |
| Unclassified *Flavobacteriaceae* | Lactose | -0.000033 | 0.000036 | 0.358 | 0.615 | -0.000103 | 0.000037 |
| Unclassified *Flavobacteriaceae* | Lactoferrin | -0.000290 | 0.000593 | 0.627 | 0.763 | -0.001453 | 0.000872 |
| Unclassified *Flavobacteriaceae* | Lysozyme | -0.002875 | 0.002972 | 0.339 | 0.615 | -0.008700 | 0.002950 |
| Unclassified *Flavobacteriaceae* | sIgA | -0.006631 | 0.003627 | 0.075 | 0.230 | -0.013739 | 0.000477 |
| Unclassified *Flavobacteriaceae* | Delivery mode: planned caesarean | -3.219148 | 2.186904 | 0.149 | 0.356 | -7.505481 | 1.067184 |
| Unclassified *Flavobacteriaceae* | Delivery mode: emergency caesarean | -1.034247 | 1.757914 | 0.560 | 0.745 | -4.479760 | 2.411265 |
| Unclassified *Flavobacteriaceae* | First week pacifier: yes | -1.814608 | 1.389894 | 0.199 | 0.428 | -4.538800 | 0.909584 |
| Unclassified *Flavobacteriaceae* | Siblings: yes | -0.758862 | 1.392982 | 0.589 | 0.745 | -3.489106 | 1.971383 |
| Unclassified *Flavobacteriaceae* | Pre-pregnancy BMI: high and obese | 0.667019 | 1.489344 | 0.657 | 0.763 | -2.252095 | 3.586133 |
| *Streptococcus parasanguinis* | Ca | -0.000009 | 0.000008 | 0.274 | 0.437 | -0.000025 | 0.000007 |
| *Streptococcus parasanguinis* | Na | -0.000011 | 0.000011 | 0.302 | 0.440 | -0.000032 | 0.000010 |
| *Streptococcus parasanguinis* | K | -0.000004 | 0.000004 | 0.376 | 0.490 | -0.000012 | 0.000005 |
| *Streptococcus parasanguinis* | P | -0.000017 | 0.000017 | 0.317 | 0.440 | -0.000050 | 0.000016 |
| *Streptococcus parasanguinis* | Mg | -0.000037 | 0.000076 | 0.625 | 0.689 | -0.000185 | 0.000111 |
| *Streptococcus parasanguinis* | I | -0.000008 | 0.000005 | 0.162 | 0.279 | -0.000018 | 0.000003 |
| *Streptococcus parasanguinis* | Se | -0.000146 | 0.000103 | 0.162 | 0.279 | -0.000348 | 0.000055 |
| *Streptococcus parasanguinis* | Cu | -0.000007 | 0.000007 | 0.339 | 0.456 | -0.000020 | 0.000007 |
| *Streptococcus parasanguinis* | Mn | -0.000431 | 0.000608 | 0.482 | 0.595 | -0.001622 | 0.000759 |
| *Streptococcus parasanguinis* | Mo | -0.000086 | 0.000278 | 0.757 | 0.775 | -0.000631 | 0.000458 |
| *Streptococcus parasanguinis* | Fe | -0.000007 | 0.000004 | 0.106 | 0.208 | -0.000015 | 0.000001 |
| *Streptococcus parasanguinis* | Zn | -0.000001 | 0.000001 | 0.484 | 0.595 | -0.000004 | 0.000002 |
| *Streptococcus parasanguinis* | Lactose | -0.000049 | 0.000031 | 0.129 | 0.240 | -0.000110 | 0.000013 |
| *Streptococcus parasanguinis* | Lactoferrin | -0.000617 | 0.000524 | 0.246 | 0.406 | -0.001644 | 0.000410 |
| *Streptococcus parasanguinis* | Lysozyme | -0.002715 | 0.002660 | 0.313 | 0.440 | -0.007927 | 0.002498 |
| *Streptococcus parasanguinis* | sIgA | 0.001982 | 0.003365 | 0.559 | 0.633 | -0.004613 | 0.008578 |
| *Streptococcus parasanguinis* | Delivery mode: planned caesarean | -0.510561 | 1.989505 | 0.799 | 0.799 | -4.409990 | 3.388868 |
| *Streptococcus parasanguinis* | Delivery mode: emergency caesarean | 0.978534 | 1.599237 | 0.544 | 0.633 | -2.155971 | 4.113039 |
| *Streptococcus parasanguinis* | First week pacifier: yes | 2.415347 | 1.191173 | 0.049 | 0.101 | 0.080647 | 4.750046 |
| *Streptococcus parasanguinis* | Siblings: yes | -0.490212 | 1.250260 | 0.697 | 0.749 | -2.940722 | 1.960297 |
| *Streptococcus parasanguinis* | Pre-pregnancy BMI: high and obese | -0.797366 | 1.333621 | 0.553 | 0.633 | -3.411264 | 1.816532 |
| *Bifidobacterium longum* | Ca | -0.000004 | 0.000005 | 0.453 | 0.527 | -0.000013 | 0.000006 |
| *Bifidobacterium longum* | Na | -0.000009 | 0.000006 | 0.181 | 0.387 | -0.000021 | 0.000004 |
| *Bifidobacterium longum* | K | -0.000002 | 0.000003 | 0.365 | 0.475 | -0.000008 | 0.000003 |
| *Bifidobacterium longum* | P | -0.000010 | 0.000010 | 0.351 | 0.475 | -0.000030 | 0.000011 |
| *Bifidobacterium longum* | Mg | -0.000057 | 0.000046 | 0.218 | 0.387 | -0.000147 | 0.000032 |
| *Bifidobacterium longum* | I | -0.000005 | 0.000003 | 0.154 | 0.368 | -0.000011 | 0.000002 |
| *Bifidobacterium longum* | Se | -0.000057 | 0.000064 | 0.377 | 0.475 | -0.000183 | 0.000069 |
| *Bifidobacterium longum* | Cu | -0.000001 | 0.000004 | 0.818 | 0.818 | -0.000009 | 0.000007 |
| *Bifidobacterium longum* | Mn | -0.000463 | 0.000370 | 0.218 | 0.387 | -0.001188 | 0.000262 |
| *Bifidobacterium longum* | Mo | -0.000178 | 0.000169 | 0.300 | 0.429 | -0.000509 | 0.000154 |
| *Bifidobacterium longum* | Fe | -0.000002 | 0.000003 | 0.363 | 0.475 | -0.000007 | 0.000003 |
| *Bifidobacterium longum* | Zn | -0.000001 | 0.000001 | 0.230 | 0.387 | -0.000003 | 0.000001 |
| *Bifidobacterium longum* | Lactose | -0.000016 | 0.000020 | 0.425 | 0.507 | -0.000055 | 0.000023 |
| *Bifidobacterium longum* | Lactoferrin | -0.000390 | 0.000323 | 0.234 | 0.387 | -0.001023 | 0.000243 |
| *Bifidobacterium longum* | Lysozyme | -0.001893 | 0.001634 | 0.253 | 0.403 | -0.005097 | 0.001310 |
| *Bifidobacterium longum* | sIgA | -0.001318 | 0.002074 | 0.529 | 0.568 | -0.005382 | 0.002747 |
| *Bifidobacterium longum* | Delivery mode: planned caesarean | -1.504128 | 1.213828 | 0.223 | 0.387 | -3.883232 | 0.874976 |
| *Bifidobacterium longum* | Delivery mode: emergency caesarean | -1.340434 | 0.975720 | 0.177 | 0.387 | -3.252846 | 0.571977 |
| *Bifidobacterium longum* | First week pacifier: yes | 0.419443 | 0.769563 | 0.589 | 0.617 | -1.088901 | 1.927787 |
| *Bifidobacterium longum* | Siblings: yes | 0.669206 | 0.765358 | 0.387 | 0.475 | -0.830897 | 2.169308 |
| *Bifidobacterium longum* | Pre-pregnancy BMI: high and obese | 0.554196 | 0.819225 | 0.503 | 0.554 | -1.051484 | 2.159876 |
| *Lactobacillus gasseri* | Ca | 0.000004 | 0.000008 | 0.618 | 0.852 | -0.000011 | 0.000019 |
| *Lactobacillus gasseri* | Na | 0.000000 | 0.000010 | 0.974 | 0.987 | -0.000019 | 0.000020 |
| *Lactobacillus gasseri* | K | 0.000002 | 0.000004 | 0.563 | 0.852 | -0.000006 | 0.000010 |
| *Lactobacillus gasseri* | P | 0.000008 | 0.000016 | 0.631 | 0.852 | -0.000023 | 0.000039 |
| *Lactobacillus gasseri* | Mg | -0.000022 | 0.000070 | 0.752 | 0.865 | -0.000161 | 0.000116 |
| *Lactobacillus gasseri* | I | -0.000005 | 0.000005 | 0.359 | 0.648 | -0.000015 | 0.000005 |
| *Lactobacillus gasseri* | Se | -0.000011 | 0.000098 | 0.911 | 0.979 | -0.000203 | 0.000181 |
| *Lactobacillus gasseri* | Cu | 0.000006 | 0.000006 | 0.325 | 0.648 | -0.000006 | 0.000019 |
| *Lactobacillus gasseri* | Mn | -0.000172 | 0.000569 | 0.764 | 0.865 | -0.001286 | 0.000943 |
| *Lactobacillus gasseri* | Mo | -0.000273 | 0.000255 | 0.291 | 0.648 | -0.000774 | 0.000227 |
| *Lactobacillus gasseri* | Fe | -0.000001 | 0.000004 | 0.781 | 0.865 | -0.000009 | 0.000007 |
| *Lactobacillus gasseri* | Zn | -0.000001 | 0.000001 | 0.348 | 0.648 | -0.000004 | 0.000001 |
| *Lactobacillus gasseri* | Lactose | -0.000013 | 0.000030 | 0.664 | 0.852 | -0.000072 | 0.000046 |
| *Lactobacillus gasseri* | Lactoferrin | -0.000541 | 0.000489 | 0.275 | 0.648 | -0.001500 | 0.000417 |
| *Lactobacillus gasseri* | Lysozyme | -0.002290 | 0.002482 | 0.362 | 0.648 | -0.007155 | 0.002576 |
| *Lactobacillus gasseri* | sIgA | -0.003557 | 0.003097 | 0.257 | 0.648 | -0.009628 | 0.002514 |
| *Lactobacillus gasseri* | Delivery mode: planned caesarean | -1.509153 | 1.854083 | 0.420 | 0.723 | -5.143156 | 2.124850 |
| *Lactobacillus gasseri* | Delivery mode: emergency caesarean | -1.559090 | 1.490381 | 0.302 | 0.648 | -4.480236 | 1.362056 |
| *Lactobacillus gasseri* | First week pacifier: yes | -0.500605 | 1.179932 | 0.674 | 0.852 | -2.813271 | 1.812062 |
| *Lactobacillus gasseri* | Siblings: yes | 0.019503 | 1.166448 | 0.987 | 0.987 | -2.266735 | 2.305742 |
| *Lactobacillus gasseri* | Pre-pregnancy BMI: high and obese | -0.400866 | 1.245709 | 0.749 | 0.865 | -2.842455 | 2.040723 |

Ca, calcium; Na, sodium; K, potassium; P, phosphorus; Mg, magnesium; I, iodine; Se, selenium; Cu, copper; Mn, manganese; Mo, molybdenum; Fe, iron; Zn, zinc; sIgA, secretory immunoglobulin A.

**Supplementary Table S9**. Post-hoc power analysis for covariate-adjusted linear regression models based on observed effect sizes using human milk component concentrations

| Response | Human milk component | R2_full | R2_covarite | F^2^ | Power |
| --- | --- | --- | --- | --- | --- |
| *Streptococcus mitis* | sIgA | 0.3013 | 0.2560 | 0.0649 | 0.3101 |
| *Streptococcus mitis* | Cu | 0.2941 | 0.2560 | 0.0541 | 0.2667 |
| *Streptococcus mitis* | I | 0.2869 | 0.2560 | 0.0433 | 0.2229 |
| *Streptococcus mitis* | Lactoferrin | 0.2842 | 0.2560 | 0.0394 | 0.2073 |
| *Streptococcus mitis* | Ca | 0.2834 | 0.2560 | 0.0382 | 0.2024 |
| *Streptococcus mitis* | Fe | 0.2825 | 0.2560 | 0.0369 | 0.1969 |
| *Streptococcus mitis* | Lysozyme | 0.2644 | 0.2560 | 0.0114 | 0.0941 |
| *Streptococcus mitis* | Zn | 0.2610 | 0.2560 | 0.0068 | 0.0760 |
| *Streptococcus mitis* | Lactose | 0.2607 | 0.2560 | 0.0063 | 0.0743 |
| *Streptococcus mitis* | Mn | 0.2605 | 0.2560 | 0.0061 | 0.0734 |
| *Streptococcus mitis* | Se | 0.2595 | 0.2560 | 0.0047 | 0.0680 |
| *Streptococcus mitis* | Mo | 0.2565 | 0.2560 | 0.0007 | 0.0526 |
| *Streptococcus mitis* | Na | 0.2564 | 0.2560 | 0.0005 | 0.0520 |
| *Streptococcus mitis* | P | 0.2561 | 0.2560 | 0.0002 | 0.0507 |
| *Streptococcus mitis* | K | 0.2560 | 0.2560 | 0.0000 | 0.0501 |
| *Streptococcus mitis* | Mg | 0.2560 | 0.2560 | 0.0000 | 0.0500 |
| *Gemella haemolysans* | Lysozyme | 0.4418 | 0.3668 | 0.1344 | 0.5579 |
| *Gemella haemolysans* | Cu | 0.4171 | 0.3668 | 0.0863 | 0.3928 |
| *Gemella haemolysans* | sIgA | 0.4051 | 0.3668 | 0.0643 | 0.3077 |
| *Gemella haemolysans* | Mg | 0.3870 | 0.3668 | 0.0329 | 0.1808 |
| *Gemella haemolysans* | Lactoferrin | 0.3848 | 0.3668 | 0.0292 | 0.1654 |
| *Gemella haemolysans* | P | 0.3776 | 0.3668 | 0.0174 | 0.1180 |
| *Gemella haemolysans* | Na | 0.3714 | 0.3668 | 0.0073 | 0.0781 |
| *Gemella haemolysans* | Lactose | 0.3708 | 0.3668 | 0.0064 | 0.0745 |
| *Gemella haemolysans* | Se | 0.3707 | 0.3668 | 0.0061 | 0.0735 |
| *Gemella haemolysans* | Mo | 0.3683 | 0.3668 | 0.0024 | 0.0590 |
| *Gemella haemolysans* | Mn | 0.3678 | 0.3668 | 0.0015 | 0.0558 |
| *Gemella haemolysans* | Fe | 0.3678 | 0.3668 | 0.0015 | 0.0557 |
| *Gemella haemolysans* | Zn | 0.3674 | 0.3668 | 0.0010 | 0.0536 |
| *Gemella haemolysans* | I | 0.3674 | 0.3668 | 0.0009 | 0.0532 |
| *Gemella haemolysans* | Ca | 0.3671 | 0.3668 | 0.0005 | 0.0518 |
| *Gemella haemolysans* | K | 0.3670 | 0.3668 | 0.0003 | 0.0511 |
| *Rothia mucilaginosa* | Lysozyme | 0.5219 | 0.4216 | 0.2097 | 0.7485 |
| *Rothia mucilaginosa* | Cu | 0.4480 | 0.4216 | 0.0478 | 0.2413 |
| *Rothia mucilaginosa* | Mg | 0.4396 | 0.4216 | 0.0322 | 0.1777 |
| *Rothia mucilaginosa* | Mn | 0.4381 | 0.4216 | 0.0294 | 0.1664 |
| *Rothia mucilaginosa* | Lactose | 0.4314 | 0.4216 | 0.0172 | 0.1171 |
| *Rothia mucilaginosa* | sIgA | 0.4314 | 0.4216 | 0.0171 | 0.1169 |
| *Rothia mucilaginosa* | Fe | 0.4308 | 0.4216 | 0.0162 | 0.1130 |
| *Rothia mucilaginosa* | Ca | 0.4286 | 0.4216 | 0.0123 | 0.0975 |
| *Rothia mucilaginosa* | I | 0.4249 | 0.4216 | 0.0057 | 0.0716 |
| *Rothia mucilaginosa* | Lactoferrin | 0.4247 | 0.4216 | 0.0054 | 0.0705 |
| *Rothia mucilaginosa* | Na | 0.4240 | 0.4216 | 0.0041 | 0.0655 |
| *Rothia mucilaginosa* | Se | 0.4234 | 0.4216 | 0.0031 | 0.0617 |
| *Rothia mucilaginosa* | P | 0.4230 | 0.4216 | 0.0025 | 0.0593 |
| *Rothia mucilaginosa* | Mo | 0.4228 | 0.4216 | 0.0020 | 0.0575 |
| *Rothia mucilaginosa* | K | 0.4221 | 0.4216 | 0.0008 | 0.0530 |
| *Rothia mucilaginosa* | Zn | 0.4217 | 0.4216 | 0.0001 | 0.0505 |
| *Streptococcus salivarius* group 1 | Mn | 0.3916 | 0.3286 | 0.1036 | 0.4557 |
| *Streptococcus salivarius* group 1 | Se | 0.3860 | 0.3286 | 0.0936 | 0.4196 |
| *Streptococcus salivarius* group 1 | Fe | 0.3756 | 0.3286 | 0.0754 | 0.3512 |
| *Streptococcus salivarius* group 1 | Lysozyme | 0.3730 | 0.3286 | 0.0709 | 0.3337 |
| *Streptococcus salivarius* group 1 | I | 0.3695 | 0.3286 | 0.0650 | 0.3104 |
| *Streptococcus salivarius* group 1 | P | 0.3639 | 0.3286 | 0.0555 | 0.2726 |
| *Streptococcus salivarius* group 1 | sIgA | 0.3598 | 0.3286 | 0.0488 | 0.2452 |
| *Streptococcus salivarius* group 1 | Zn | 0.3587 | 0.3286 | 0.0470 | 0.2381 |
| *Streptococcus salivarius* group 1 | Mo | 0.3499 | 0.3286 | 0.0328 | 0.1805 |
| *Streptococcus salivarius* group 1 | Lactoferrin | 0.3436 | 0.3286 | 0.0230 | 0.1403 |
| *Streptococcus salivarius* group 1 | Na | 0.3407 | 0.3286 | 0.0184 | 0.1220 |
| *Streptococcus salivarius* group 1 | Ca | 0.3371 | 0.3286 | 0.0128 | 0.0997 |
| *Streptococcus salivarius* group 1 | Cu | 0.3370 | 0.3286 | 0.0127 | 0.0992 |
| *Streptococcus salivarius* group 1 | Lactose | 0.3334 | 0.3286 | 0.0072 | 0.0776 |
| *Streptococcus salivarius* group 1 | Mg | 0.3310 | 0.3286 | 0.0036 | 0.0637 |
| *Streptococcus salivarius* group 1 | K | 0.3287 | 0.3286 | 0.0002 | 0.0509 |
| *Veillonella* sp. | Mo | 0.3663 | 0.2750 | 0.1440 | 0.5868 |
| *Veillonella* sp. | I | 0.3594 | 0.2750 | 0.1316 | 0.5493 |
| *Veillonella* sp. | Mg | 0.3276 | 0.2750 | 0.0782 | 0.3618 |
| *Veillonella* sp. | Lysozyme | 0.3272 | 0.2750 | 0.0776 | 0.3596 |
| *Veillonella* sp. | Lactose | 0.3266 | 0.2750 | 0.0765 | 0.3556 |
| *Veillonella* sp. | Zn | 0.3183 | 0.2750 | 0.0634 | 0.3041 |
| *Veillonella* sp. | Fe | 0.3027 | 0.2750 | 0.0397 | 0.2083 |
| *Veillonella* sp. | Ca | 0.2908 | 0.2750 | 0.0222 | 0.1374 |
| *Veillonella* sp. | sIgA | 0.2860 | 0.2750 | 0.0154 | 0.1098 |
| *Veillonella* sp. | Se | 0.2802 | 0.2750 | 0.0072 | 0.0778 |
| *Veillonella* sp. | Na | 0.2789 | 0.2750 | 0.0054 | 0.0707 |
| *Veillonella* sp. | P | 0.2780 | 0.2750 | 0.0041 | 0.0655 |
| *Veillonella* sp. | Cu | 0.2764 | 0.2750 | 0.0018 | 0.0569 |
| *Veillonella* sp. | Mn | 0.2761 | 0.2750 | 0.0015 | 0.0558 |
| *Veillonella* sp. | Lactoferrin | 0.2760 | 0.2750 | 0.0013 | 0.0550 |
| *Veillonella* sp. | K | 0.2750 | 0.2750 | 0.0000 | 0.0500 |
| *Neisseria subflava* | Fe | 0.3182 | 0.2479 | 0.1031 | 0.4539 |
| *Neisseria subflava* | P | 0.3163 | 0.2479 | 0.1000 | 0.4431 |
| *Neisseria subflava* | Mn | 0.3150 | 0.2479 | 0.0979 | 0.4355 |
| *Neisseria subflava* | K | 0.3122 | 0.2479 | 0.0935 | 0.4196 |
| *Neisseria subflava* | Mg | 0.2847 | 0.2479 | 0.0514 | 0.2559 |
| *Neisseria subflava* | Mo | 0.2745 | 0.2479 | 0.0367 | 0.1960 |
| *Neisseria subflava* | Se | 0.2682 | 0.2479 | 0.0277 | 0.1596 |
| *Neisseria subflava* | I | 0.2607 | 0.2479 | 0.0173 | 0.1175 |
| *Neisseria subflava* | sIgA | 0.2575 | 0.2479 | 0.0129 | 0.0998 |
| *Neisseria subflava* | Zn | 0.2543 | 0.2479 | 0.0086 | 0.0831 |
| *Neisseria subflava* | Lactoferrin | 0.2532 | 0.2479 | 0.0070 | 0.0769 |
| *Neisseria subflava* | Cu | 0.2507 | 0.2479 | 0.0037 | 0.0641 |
| *Neisseria subflava* | Ca | 0.2500 | 0.2479 | 0.0028 | 0.0608 |
| *Neisseria subflava* | Lysozyme | 0.2492 | 0.2479 | 0.0017 | 0.0566 |
| *Neisseria subflava* | Na | 0.2487 | 0.2479 | 0.0010 | 0.0538 |
| *Neisseria subflava* | Lactose | 0.2480 | 0.2479 | 0.0001 | 0.0505 |
| *Streptococcus oralis* | Mg | 0.2991 | 0.2165 | 0.1179 | 0.5050 |
| *Streptococcus oralis* | sIgA | 0.2724 | 0.2165 | 0.0769 | 0.3568 |
| *Streptococcus oralis* | Cu | 0.2715 | 0.2165 | 0.0755 | 0.3517 |
| *Streptococcus oralis* | Na | 0.2644 | 0.2165 | 0.0651 | 0.3108 |
| *Streptococcus oralis* | Lactose | 0.2640 | 0.2165 | 0.0646 | 0.3088 |
| *Streptococcus oralis* | Mo | 0.2468 | 0.2165 | 0.0403 | 0.2110 |
| *Streptococcus oralis* | Lysozyme | 0.2384 | 0.2165 | 0.0287 | 0.1637 |
| *Streptococcus oralis* | K | 0.2351 | 0.2165 | 0.0243 | 0.1457 |
| *Streptococcus oralis* | Zn | 0.2250 | 0.2165 | 0.0110 | 0.0927 |
| *Streptococcus oralis* | Fe | 0.2225 | 0.2165 | 0.0077 | 0.0796 |
| *Streptococcus oralis* | Mn | 0.2212 | 0.2165 | 0.0061 | 0.0732 |
| *Streptococcus oralis* | Ca | 0.2173 | 0.2165 | 0.0011 | 0.0540 |
| *Streptococcus oralis* | I | 0.2172 | 0.2165 | 0.0010 | 0.0537 |
| *Streptococcus oralis* | Lactoferrin | 0.2169 | 0.2165 | 0.0006 | 0.0522 |
| *Streptococcus oralis* | P | 0.2168 | 0.2165 | 0.0004 | 0.0514 |
| *Streptococcus oralis* | Se | 0.2167 | 0.2165 | 0.0003 | 0.0510 |
| *Haemophilus haemolyticus* | Mn | 0.2673 | 0.0807 | 0.2548 | 0.8260 |
| *Haemophilus haemolyticus* | Se | 0.2280 | 0.0807 | 0.1909 | 0.7086 |
| *Haemophilus haemolyticus* | P | 0.2213 | 0.0807 | 0.1806 | 0.6845 |
| *Haemophilus haemolyticus* | Fe | 0.1854 | 0.0807 | 0.1285 | 0.5396 |
| *Haemophilus haemolyticus* | Lysozyme | 0.1732 | 0.0807 | 0.1120 | 0.4849 |
| *Haemophilus haemolyticus* | sIgA | 0.1225 | 0.0807 | 0.0476 | 0.2407 |
| *Haemophilus haemolyticus* | Ca | 0.1102 | 0.0807 | 0.0333 | 0.1821 |
| *Haemophilus haemolyticus* | Mg | 0.0991 | 0.0807 | 0.0204 | 0.1301 |
| *Haemophilus haemolyticus* | Zn | 0.0954 | 0.0807 | 0.0163 | 0.1137 |
| *Haemophilus haemolyticus* | K | 0.0937 | 0.0807 | 0.0144 | 0.1059 |
| *Haemophilus haemolyticus* | Lactoferrin | 0.0860 | 0.0807 | 0.0059 | 0.0725 |
| *Haemophilus haemolyticus* | Cu | 0.0857 | 0.0807 | 0.0055 | 0.0712 |
| *Haemophilus haemolyticus* | Lactose | 0.0825 | 0.0807 | 0.0020 | 0.0577 |
| *Haemophilus haemolyticus* | I | 0.0824 | 0.0807 | 0.0019 | 0.0571 |
| *Haemophilus haemolyticus* | Na | 0.0816 | 0.0807 | 0.0010 | 0.0537 |
| *Haemophilus haemolyticus* | Mo | 0.0809 | 0.0807 | 0.0002 | 0.0509 |
| *Veillonella nakazawae* | P | 0.2480 | 0.1138 | 0.1784 | 0.6792 |
| *Veillonella nakazawae* | Lysozyme | 0.2222 | 0.1138 | 0.1394 | 0.5730 |
| *Veillonella nakazawae* | Fe | 0.2214 | 0.1138 | 0.1382 | 0.5694 |
| *Veillonella nakazawae* | Se | 0.2202 | 0.1138 | 0.1364 | 0.5642 |
| *Veillonella nakazawae* | Na | 0.1632 | 0.1138 | 0.0589 | 0.2863 |
| *Veillonella nakazawae* | I | 0.1629 | 0.1138 | 0.0586 | 0.2848 |
| *Veillonella nakazawae* | Mn | 0.1576 | 0.1138 | 0.0519 | 0.2581 |
| *Veillonella nakazawae* | K | 0.1535 | 0.1138 | 0.0469 | 0.2378 |
| *Veillonella nakazawae* | sIgA | 0.1481 | 0.1138 | 0.0402 | 0.2105 |
| *Veillonella nakazawae* | Lactoferrin | 0.1439 | 0.1138 | 0.0351 | 0.1898 |
| *Veillonella nakazawae* | Mg | 0.1312 | 0.1138 | 0.0200 | 0.1285 |
| *Veillonella nakazawae* | Zn | 0.1202 | 0.1138 | 0.0073 | 0.0779 |
| *Veillonella nakazawae* | Ca | 0.1186 | 0.1138 | 0.0054 | 0.0706 |
| *Veillonella nakazawae* | Lactose | 0.1155 | 0.1138 | 0.0019 | 0.0572 |
| *Veillonella nakazawae* | Mo | 0.1153 | 0.1138 | 0.0016 | 0.0561 |
| *Veillonella nakazawae* | Cu | 0.1142 | 0.1138 | 0.0004 | 0.0515 |
| *Staphylococcus lugdunensis* | I | 0.2150 | 0.1281 | 0.1107 | 0.4805 |
| *Staphylococcus lugdunensis* | Lysozyme | 0.2142 | 0.1281 | 0.1096 | 0.4768 |
| *Staphylococcus lugdunensis* | Mo | 0.1559 | 0.1281 | 0.0329 | 0.1808 |
| *Staphylococcus lugdunensis* | Lactoferrin | 0.1550 | 0.1281 | 0.0319 | 0.1765 |
| *Staphylococcus lugdunensis* | Lactose | 0.1510 | 0.1281 | 0.0269 | 0.1563 |
| *Staphylococcus lugdunensis* | Zn | 0.1481 | 0.1281 | 0.0234 | 0.1422 |
| *Staphylococcus lugdunensis* | Ca | 0.1453 | 0.1281 | 0.0201 | 0.1288 |
| *Staphylococcus lugdunensis* | Cu | 0.1411 | 0.1281 | 0.0151 | 0.1089 |
| *Staphylococcus lugdunensis* | sIgA | 0.1308 | 0.1281 | 0.0031 | 0.0618 |
| *Staphylococcus lugdunensis* | Se | 0.1298 | 0.1281 | 0.0019 | 0.0574 |
| *Staphylococcus lugdunensis* | P | 0.1291 | 0.1281 | 0.0011 | 0.0542 |
| *Staphylococcus lugdunensis* | Mn | 0.1288 | 0.1281 | 0.0008 | 0.0532 |
| *Staphylococcus lugdunensis* | K | 0.1288 | 0.1281 | 0.0008 | 0.0532 |
| *Staphylococcus lugdunensis* | Na | 0.1286 | 0.1281 | 0.0005 | 0.0520 |
| *Staphylococcus lugdunensis* | Fe | 0.1282 | 0.1281 | 0.0001 | 0.0505 |
| *Staphylococcus lugdunensis* | Mg | 0.1281 | 0.1281 | 0.0000 | 0.0500 |
| *Haemophilus parainfluenzae* | sIgA | 0.1869 | 0.1505 | 0.0447 | 0.2288 |
| *Haemophilus parainfluenzae* | Na | 0.1757 | 0.1505 | 0.0305 | 0.1709 |
| *Haemophilus parainfluenzae* | Lactoferrin | 0.1713 | 0.1505 | 0.0251 | 0.1490 |
| *Haemophilus parainfluenzae* | Lysozyme | 0.1711 | 0.1505 | 0.0248 | 0.1478 |
| *Haemophilus parainfluenzae* | Mg | 0.1709 | 0.1505 | 0.0246 | 0.1468 |
| *Haemophilus parainfluenzae* | Cu | 0.1661 | 0.1505 | 0.0187 | 0.1231 |
| *Haemophilus parainfluenzae* | I | 0.1633 | 0.1505 | 0.0153 | 0.1095 |
| *Haemophilus parainfluenzae* | Mn | 0.1619 | 0.1505 | 0.0136 | 0.1028 |
| *Haemophilus parainfluenzae* | Mo | 0.1602 | 0.1505 | 0.0115 | 0.0945 |
| *Haemophilus parainfluenzae* | Se | 0.1560 | 0.1505 | 0.0065 | 0.0750 |
| *Haemophilus parainfluenzae* | K | 0.1557 | 0.1505 | 0.0061 | 0.0732 |
| *Haemophilus parainfluenzae* | Zn | 0.1554 | 0.1505 | 0.0057 | 0.0720 |
| *Haemophilus parainfluenzae* | Lactose | 0.1551 | 0.1505 | 0.0054 | 0.0705 |
| *Haemophilus parainfluenzae* | Ca | 0.1547 | 0.1505 | 0.0050 | 0.0690 |
| *Haemophilus parainfluenzae* | P | 0.1506 | 0.1505 | 0.0000 | 0.0502 |
| *Haemophilus parainfluenzae* | Fe | 0.1506 | 0.1505 | 0.0000 | 0.0501 |
| Unknown *Flavobacteriaceae* sp. | Ca | 0.3170 | 0.1265 | 0.2788 | 0.8581 |
| Unknown *Flavobacteriaceae* sp. | Cu | 0.2079 | 0.1265 | 0.1027 | 0.4524 |
| Unknown *Flavobacteriaceae* sp. | Mg | 0.2020 | 0.1265 | 0.0945 | 0.4232 |
| Unknown *Flavobacteriaceae* sp. | sIgA | 0.1966 | 0.1265 | 0.0871 | 0.3959 |
| Unknown *Flavobacteriaceae* sp. | K | 0.1747 | 0.1265 | 0.0583 | 0.2837 |
| Unknown *Flavobacteriaceae* sp. | Mn | 0.1709 | 0.1265 | 0.0535 | 0.2646 |
| Unknown *Flavobacteriaceae* sp. | Lysozyme | 0.1554 | 0.1265 | 0.0342 | 0.1858 |
| Unknown *Flavobacteriaceae* sp. | Mo | 0.1408 | 0.1265 | 0.0166 | 0.1149 |
| Unknown *Flavobacteriaceae* sp. | Fe | 0.1399 | 0.1265 | 0.0155 | 0.1103 |
| Unknown *Flavobacteriaceae* sp. | Zn | 0.1375 | 0.1265 | 0.0127 | 0.0994 |
| Unknown *Flavobacteriaceae* sp. | Lactoferrin | 0.1361 | 0.1265 | 0.0111 | 0.0930 |
| Unknown *Flavobacteriaceae* sp. | P | 0.1280 | 0.1265 | 0.0016 | 0.0562 |
| Unknown *Flavobacteriaceae* sp. | Lactose | 0.1275 | 0.1265 | 0.0011 | 0.0542 |
| Unknown *Flavobacteriaceae* sp. | Se | 0.1270 | 0.1265 | 0.0005 | 0.0520 |
| Unknown *Flavobacteriaceae* sp. | Na | 0.1267 | 0.1265 | 0.0002 | 0.0507 |
| Unknown *Flavobacteriaceae* sp. | I | 0.1267 | 0.1265 | 0.0002 | 0.0507 |
| *Streptococcus parasanguinis* | Fe | 0.2255 | 0.1655 | 0.0775 | 0.3593 |
| *Streptococcus parasanguinis* | Mg | 0.2172 | 0.1655 | 0.0661 | 0.3147 |
| *Streptococcus parasanguinis* | I | 0.2151 | 0.1655 | 0.0632 | 0.3034 |
| *Streptococcus parasanguinis* | Cu | 0.2092 | 0.1655 | 0.0553 | 0.2716 |
| *Streptococcus parasanguinis* | Se | 0.2042 | 0.1655 | 0.0486 | 0.2447 |
| *Streptococcus parasanguinis* | Lactoferrin | 0.2038 | 0.1655 | 0.0481 | 0.2426 |
| *Streptococcus parasanguinis* | Lysozyme | 0.1951 | 0.1655 | 0.0368 | 0.1966 |
| *Streptococcus parasanguinis* | Zn | 0.1844 | 0.1655 | 0.0232 | 0.1411 |
| *Streptococcus parasanguinis* | sIgA | 0.1805 | 0.1655 | 0.0183 | 0.1216 |
| *Streptococcus parasanguinis* | Ca | 0.1777 | 0.1655 | 0.0148 | 0.1077 |
| *Streptococcus parasanguinis* | Mn | 0.1717 | 0.1655 | 0.0075 | 0.0788 |
| *Streptococcus parasanguinis* | Na | 0.1695 | 0.1655 | 0.0048 | 0.0685 |
| *Streptococcus parasanguinis* | Mo | 0.1682 | 0.1655 | 0.0032 | 0.0622 |
| *Streptococcus parasanguinis* | K | 0.1673 | 0.1655 | 0.0022 | 0.0582 |
| *Streptococcus parasanguinis* | Lactose | 0.1672 | 0.1655 | 0.0021 | 0.0579 |
| *Streptococcus parasanguinis* | P | 0.1656 | 0.1655 | 0.0001 | 0.0504 |
| *Bifidobacterium longum* | I | 0.4294 | 0.2918 | 0.2411 | 0.8049 |
| *Bifidobacterium longum* | Lactoferrin | 0.4157 | 0.2918 | 0.2120 | 0.7530 |
| *Bifidobacterium longum* | Ca | 0.3258 | 0.2918 | 0.0504 | 0.2520 |
| *Bifidobacterium longum* | Mn | 0.3255 | 0.2918 | 0.0499 | 0.2497 |
| *Bifidobacterium longum* | K | 0.3072 | 0.2918 | 0.0222 | 0.1371 |
| *Bifidobacterium longum* | Se | 0.3024 | 0.2918 | 0.0152 | 0.1091 |
| *Bifidobacterium longum* | Cu | 0.3014 | 0.2918 | 0.0137 | 0.1034 |
| *Bifidobacterium longum* | Fe | 0.2987 | 0.2918 | 0.0097 | 0.0876 |
| *Bifidobacterium longum* | Lysozyme | 0.2976 | 0.2918 | 0.0082 | 0.0817 |
| *Bifidobacterium longum* | Lactose | 0.2968 | 0.2918 | 0.0071 | 0.0772 |
| *Bifidobacterium longum* | Mg | 0.2962 | 0.2918 | 0.0062 | 0.0738 |
| *Bifidobacterium longum* | Zn | 0.2949 | 0.2918 | 0.0043 | 0.0664 |
| *Bifidobacterium longum* | P | 0.2947 | 0.2918 | 0.0041 | 0.0656 |
| *Bifidobacterium longum* | sIgA | 0.2940 | 0.2918 | 0.0031 | 0.0619 |
| *Bifidobacterium longum* | Mo | 0.2923 | 0.2918 | 0.0006 | 0.0523 |
| *Bifidobacterium longum* | Na | 0.2923 | 0.2918 | 0.0006 | 0.0522 |
| *Lactobacillus gasseri* | I | 0.2383 | 0.1623 | 0.0998 | 0.4422 |
| *Lactobacillus gasseri* | Zn | 0.2030 | 0.1623 | 0.0511 | 0.2546 |
| *Lactobacillus gasseri* | Lactose | 0.2008 | 0.1623 | 0.0482 | 0.2431 |
| *Lactobacillus gasseri* | Mn | 0.1988 | 0.1623 | 0.0456 | 0.2323 |
| *Lactobacillus gasseri* | Cu | 0.1980 | 0.1623 | 0.0446 | 0.2283 |
| *Lactobacillus gasseri* | sIgA | 0.1971 | 0.1623 | 0.0434 | 0.2235 |
| *Lactobacillus gasseri* | Mo | 0.1865 | 0.1623 | 0.0298 | 0.1680 |
| *Lactobacillus gasseri* | Lactoferrin | 0.1825 | 0.1623 | 0.0247 | 0.1473 |
| *Lactobacillus gasseri* | Fe | 0.1821 | 0.1623 | 0.0242 | 0.1455 |
| *Lactobacillus gasseri* | Lysozyme | 0.1804 | 0.1623 | 0.0221 | 0.1370 |
| *Lactobacillus gasseri* | Se | 0.1769 | 0.1623 | 0.0178 | 0.1195 |
| *Lactobacillus gasseri* | Mg | 0.1723 | 0.1623 | 0.0121 | 0.0968 |
| *Lactobacillus gasseri* | P | 0.1686 | 0.1623 | 0.0076 | 0.0793 |
| *Lactobacillus gasseri* | K | 0.1669 | 0.1623 | 0.0055 | 0.0711 |
| *Lactobacillus gasseri* | Ca | 0.1651 | 0.1623 | 0.0034 | 0.0631 |
| *Lactobacillus gasseri* | Na | 0.1630 | 0.1623 | 0.0008 | 0.0531 |
| Shannon diversity | Lactoferrin | 0.3596 | 0.1800 | 0.2805 | 0.8602 |
| Shannon diversity | I | 0.2512 | 0.1800 | 0.0951 | 0.4254 |
| Shannon diversity | sIgA | 0.2413 | 0.1800 | 0.0808 | 0.3720 |
| Shannon diversity | Na | 0.2225 | 0.1800 | 0.0546 | 0.2689 |
| Shannon diversity | Mg | 0.2105 | 0.1800 | 0.0386 | 0.2039 |
| Shannon diversity | Fe | 0.2012 | 0.1800 | 0.0265 | 0.1548 |
| Shannon diversity | P | 0.1955 | 0.1800 | 0.0193 | 0.1256 |
| Shannon diversity | Mn | 0.1842 | 0.1800 | 0.0051 | 0.0696 |
| Shannon diversity | Zn | 0.1836 | 0.1800 | 0.0045 | 0.0670 |
| Shannon diversity | Mo | 0.1822 | 0.1800 | 0.0027 | 0.0602 |
| Shannon diversity | Lysozyme | 0.1821 | 0.1800 | 0.0025 | 0.0597 |
| Shannon diversity | Se | 0.1819 | 0.1800 | 0.0024 | 0.0589 |
| Shannon diversity | Lactose | 0.1819 | 0.1800 | 0.0023 | 0.0587 |
| Shannon diversity | K | 0.1811 | 0.1800 | 0.0013 | 0.0551 |
| Shannon diversity | Ca | 0.1807 | 0.1800 | 0.0009 | 0.0534 |
| Shannon diversity | Cu | 0.1805 | 0.1800 | 0.0007 | 0.0525 |
| Richness | Lactoferrin | 0.3002 | 0.1716 | 0.1837 | 0.6920 |
| Richness | I | 0.2370 | 0.1716 | 0.0857 | 0.3906 |
| Richness | Na | 0.2221 | 0.1716 | 0.0650 | 0.3103 |
| Richness | Fe | 0.2199 | 0.1716 | 0.0618 | 0.2979 |
| Richness | Mg | 0.2167 | 0.1716 | 0.0575 | 0.2805 |
| Richness | sIgA | 0.2086 | 0.1716 | 0.0467 | 0.2367 |
| Richness | P | 0.1983 | 0.1716 | 0.0333 | 0.1823 |
| Richness | Mn | 0.1870 | 0.1716 | 0.0189 | 0.1239 |
| Richness | Ca | 0.1863 | 0.1716 | 0.0180 | 0.1204 |
| Richness | Mo | 0.1741 | 0.1716 | 0.0030 | 0.0613 |
| Richness | Zn | 0.1739 | 0.1716 | 0.0027 | 0.0604 |
| Richness | Lysozyme | 0.1734 | 0.1716 | 0.0022 | 0.0582 |
| Richness | Lactose | 0.1732 | 0.1716 | 0.0019 | 0.0574 |
| Richness | Cu | 0.1719 | 0.1716 | 0.0004 | 0.0513 |
| Richness | Se | 0.1718 | 0.1716 | 0.0002 | 0.0506 |
| Richness | K | 0.1716 | 0.1716 | 0.0000 | 0.0500 |

R²_full: Coefficient of determination from the full model including the milk component and covariates; R²_covariate: Coefficient of determination from the covariate-adjusted model excluding the milk component; F² = effect size.

**Supplementary Table S10**. Post-hoc power analysis for covariate-adjusted linear regression models based on observed effect sizes using human milk component intakes

| Response | Human milk component | R2_full | R2_covarite | F^2^ | Power |
| --- | --- | --- | --- | --- | --- |
| *Streptococcus mitis* | Lactoferrin | 0.2469 | 0.1755 | 0.0949 | 0.4244 |
| *Streptococcus mitis* | I | 0.2004 | 0.1755 | 0.0312 | 0.1736 |
| *Streptococcus mitis* | Fe | 0.2001 | 0.1755 | 0.0308 | 0.1721 |
| *Streptococcus mitis* | sIgA | 0.1911 | 0.1755 | 0.0193 | 0.1257 |
| *Streptococcus mitis* | Ca | 0.1907 | 0.1755 | 0.0188 | 0.1236 |
| *Streptococcus mitis* | Zn | 0.1882 | 0.1755 | 0.0157 | 0.1112 |
| *Streptococcus mitis* | Lactose | 0.1863 | 0.1755 | 0.0133 | 0.1017 |
| *Streptococcus mitis* | Cu | 0.1808 | 0.1755 | 0.0064 | 0.0747 |
| *Streptococcus mitis* | Na | 0.1794 | 0.1755 | 0.0048 | 0.0684 |
| *Streptococcus mitis* | Se | 0.1791 | 0.1755 | 0.0044 | 0.0667 |
| *Streptococcus mitis* | P | 0.1781 | 0.1755 | 0.0032 | 0.0623 |
| *Streptococcus mitis* | K | 0.1774 | 0.1755 | 0.0024 | 0.0589 |
| *Streptococcus mitis* | Mo | 0.1770 | 0.1755 | 0.0018 | 0.0567 |
| *Streptococcus mitis* | Lysozyme | 0.1768 | 0.1755 | 0.0016 | 0.0559 |
| *Streptococcus mitis* | Mn | 0.1758 | 0.1755 | 0.0003 | 0.0512 |
| *Streptococcus mitis* | Mg | 0.1755 | 0.1755 | 0.0000 | 0.0500 |
| *Gemella haemolysans* | P | 0.3447 | 0.2352 | 0.1671 | 0.6509 |
| *Gemella haemolysans* | Cu | 0.3447 | 0.2352 | 0.1670 | 0.6505 |
| *Gemella haemolysans* | K | 0.3440 | 0.2352 | 0.1658 | 0.6475 |
| *Gemella haemolysans* | Ca | 0.3332 | 0.2352 | 0.1469 | 0.5955 |
| *Gemella haemolysans* | Zn | 0.3268 | 0.2352 | 0.1360 | 0.5628 |
| *Gemella haemolysans* | Se | 0.3243 | 0.2352 | 0.1319 | 0.5501 |
| *Gemella haemolysans* | Lactose | 0.3108 | 0.2352 | 0.1097 | 0.4770 |
| *Gemella haemolysans* | sIgA | 0.2761 | 0.2352 | 0.0565 | 0.2763 |
| *Gemella haemolysans* | Mn | 0.2683 | 0.2352 | 0.0453 | 0.2310 |
| *Gemella haemolysans* | I | 0.2636 | 0.2352 | 0.0385 | 0.2034 |
| *Gemella haemolysans* | Mg | 0.2606 | 0.2352 | 0.0343 | 0.1865 |
| *Gemella haemolysans* | Fe | 0.2589 | 0.2352 | 0.0319 | 0.1766 |
| *Gemella haemolysans* | Na | 0.2566 | 0.2352 | 0.0288 | 0.1639 |
| *Gemella haemolysans* | Lysozyme | 0.2438 | 0.2352 | 0.0114 | 0.0940 |
| *Gemella haemolysans* | Lactoferrin | 0.2370 | 0.2352 | 0.0024 | 0.0590 |
| *Gemella haemolysans* | Mo | 0.2352 | 0.2352 | 0.0000 | 0.0500 |
| *Rothia mucilaginosa* | Lysozyme | 0.4271 | 0.3836 | 0.0758 | 0.3529 |
| *Rothia mucilaginosa* | sIgA | 0.4224 | 0.3836 | 0.0671 | 0.3188 |
| *Rothia mucilaginosa* | Fe | 0.4194 | 0.3836 | 0.0616 | 0.2970 |
| *Rothia mucilaginosa* | Mn | 0.4188 | 0.3836 | 0.0606 | 0.2929 |
| *Rothia mucilaginosa* | Na | 0.3981 | 0.3836 | 0.0241 | 0.1450 |
| *Rothia mucilaginosa* | Lactoferrin | 0.3957 | 0.3836 | 0.0199 | 0.1281 |
| *Rothia mucilaginosa* | Lactose | 0.3881 | 0.3836 | 0.0073 | 0.0781 |
| *Rothia mucilaginosa* | Ca | 0.3877 | 0.3836 | 0.0066 | 0.0752 |
| *Rothia mucilaginosa* | Se | 0.3869 | 0.3836 | 0.0054 | 0.0706 |
| *Rothia mucilaginosa* | P | 0.3856 | 0.3836 | 0.0033 | 0.0625 |
| *Rothia mucilaginosa* | Mo | 0.3849 | 0.3836 | 0.0021 | 0.0578 |
| *Rothia mucilaginosa* | I | 0.3840 | 0.3836 | 0.0006 | 0.0521 |
| *Rothia mucilaginosa* | Cu | 0.3839 | 0.3836 | 0.0004 | 0.0513 |
| *Rothia mucilaginosa* | K | 0.3838 | 0.3836 | 0.0003 | 0.0511 |
| *Rothia mucilaginosa* | Zn | 0.3837 | 0.3836 | 0.0001 | 0.0502 |
| *Rothia mucilaginosa* | Mg | 0.3837 | 0.3836 | 0.0000 | 0.0501 |
| *Streptococcus salivarius* group 1 | Mn | 0.1077 | 0.0550 | 0.0591 | 0.2871 |
| *Streptococcus salivarius* group 1 | Zn | 0.1007 | 0.0550 | 0.0508 | 0.2536 |
| *Streptococcus salivarius* group 1 | Lysozyme | 0.1005 | 0.0550 | 0.0507 | 0.2530 |
| *Streptococcus salivarius* group 1 | Fe | 0.0873 | 0.0550 | 0.0354 | 0.1909 |
| *Streptococcus salivarius* group 1 | sIgA | 0.0870 | 0.0550 | 0.0351 | 0.1897 |
| *Streptococcus salivarius* group 1 | Na | 0.0824 | 0.0550 | 0.0299 | 0.1685 |
| *Streptococcus salivarius* group 1 | Se | 0.0737 | 0.0550 | 0.0202 | 0.1292 |
| *Streptococcus salivarius* group 1 | I | 0.0724 | 0.0550 | 0.0188 | 0.1235 |
| *Streptococcus salivarius* group 1 | Lactoferrin | 0.0668 | 0.0550 | 0.0127 | 0.0994 |
| *Streptococcus salivarius* group 1 | Mg | 0.0657 | 0.0550 | 0.0115 | 0.0946 |
| *Streptococcus salivarius* group 1 | P | 0.0630 | 0.0550 | 0.0085 | 0.0829 |
| *Streptococcus salivarius* group 1 | Mo | 0.0594 | 0.0550 | 0.0048 | 0.0682 |
| *Streptococcus salivarius* group 1 | Lactose | 0.0584 | 0.0550 | 0.0037 | 0.0640 |
| *Streptococcus salivarius* group 1 | Cu | 0.0572 | 0.0550 | 0.0024 | 0.0591 |
| *Streptococcus salivarius* group 1 | K | 0.0563 | 0.0550 | 0.0014 | 0.0554 |
| *Streptococcus salivarius* group 1 | Ca | 0.0553 | 0.0550 | 0.0003 | 0.0512 |
| *Veillonella* sp. | Lysozyme | 0.4047 | 0.2453 | 0.2679 | 0.8442 |
| *Veillonella* sp. | I | 0.3740 | 0.2453 | 0.2057 | 0.7403 |
| *Veillonella* sp. | Mo | 0.3540 | 0.2453 | 0.1684 | 0.6541 |
| *Veillonella* sp. | Se | 0.3216 | 0.2453 | 0.1125 | 0.4867 |
| *Veillonella* sp. | Fe | 0.3215 | 0.2453 | 0.1123 | 0.4861 |
| *Veillonella* sp. | P | 0.3110 | 0.2453 | 0.0954 | 0.4265 |
| *Veillonella* sp. | K | 0.3020 | 0.2453 | 0.0812 | 0.3736 |
| *Veillonella* sp. | Cu | 0.2998 | 0.2453 | 0.0779 | 0.3609 |
| *Veillonella* sp. | sIgA | 0.2813 | 0.2453 | 0.0502 | 0.2510 |
| *Veillonella* sp. | Ca | 0.2753 | 0.2453 | 0.0414 | 0.2154 |
| *Veillonella* sp. | Na | 0.2647 | 0.2453 | 0.0264 | 0.1542 |
| *Veillonella* sp. | Mn | 0.2633 | 0.2453 | 0.0245 | 0.1465 |
| *Veillonella* sp. | Lactoferrin | 0.2612 | 0.2453 | 0.0216 | 0.1346 |
| *Veillonella* sp. | Lactose | 0.2548 | 0.2453 | 0.0128 | 0.0995 |
| *Veillonella* sp. | Mg | 0.2516 | 0.2453 | 0.0084 | 0.0824 |
| *Veillonella* sp. | Zn | 0.2472 | 0.2453 | 0.0025 | 0.0596 |
| *Neisseria subflava* | Mn | 0.2437 | 0.1215 | 0.1616 | 0.6363 |
| *Neisseria subflava* | Fe | 0.2261 | 0.1215 | 0.1351 | 0.5599 |
| *Neisseria subflava* | P | 0.1678 | 0.1215 | 0.0555 | 0.2726 |
| *Neisseria subflava* | Mg | 0.1602 | 0.1215 | 0.0460 | 0.2340 |
| *Neisseria subflava* | Mo | 0.1506 | 0.1215 | 0.0342 | 0.1861 |
| *Neisseria subflava* | K | 0.1503 | 0.1215 | 0.0338 | 0.1843 |
| *Neisseria subflava* | Lactose | 0.1466 | 0.1215 | 0.0293 | 0.1662 |
| *Neisseria subflava* | Se | 0.1375 | 0.1215 | 0.0185 | 0.1223 |
| *Neisseria subflava* | Zn | 0.1345 | 0.1215 | 0.0149 | 0.1081 |
| *Neisseria subflava* | I | 0.1343 | 0.1215 | 0.0148 | 0.1075 |
| *Neisseria subflava* | Na | 0.1273 | 0.1215 | 0.0066 | 0.0752 |
| *Neisseria subflava* | sIgA | 0.1238 | 0.1215 | 0.0026 | 0.0598 |
| *Neisseria subflava* | Cu | 0.1235 | 0.1215 | 0.0022 | 0.0585 |
| *Neisseria subflava* | Ca | 0.1232 | 0.1215 | 0.0019 | 0.0574 |
| *Neisseria subflava* | Lactoferrin | 0.1223 | 0.1215 | 0.0009 | 0.0534 |
| *Neisseria subflava* | Lysozyme | 0.1217 | 0.1215 | 0.0001 | 0.0505 |
| *Streptococcus oralis* | Lactose | 0.3378 | 0.2176 | 0.1816 | 0.6870 |
| *Streptococcus oralis* | Zn | 0.3139 | 0.2176 | 0.1405 | 0.5764 |
| *Streptococcus oralis* | Ca | 0.2892 | 0.2176 | 0.1007 | 0.4456 |
| *Streptococcus oralis* | P | 0.2880 | 0.2176 | 0.0989 | 0.4389 |
| *Streptococcus oralis* | Cu | 0.2799 | 0.2176 | 0.0865 | 0.3936 |
| *Streptococcus oralis* | K | 0.2534 | 0.2176 | 0.0480 | 0.2421 |
| *Streptococcus oralis* | sIgA | 0.2353 | 0.2176 | 0.0232 | 0.1413 |
| *Streptococcus oralis* | Mg | 0.2347 | 0.2176 | 0.0224 | 0.1380 |
| *Streptococcus oralis* | Na | 0.2338 | 0.2176 | 0.0212 | 0.1334 |
| *Streptococcus oralis* | Mo | 0.2330 | 0.2176 | 0.0201 | 0.1287 |
| *Streptococcus oralis* | Se | 0.2259 | 0.2176 | 0.0107 | 0.0914 |
| *Streptococcus oralis* | Lysozyme | 0.2206 | 0.2176 | 0.0039 | 0.0650 |
| *Streptococcus oralis* | Fe | 0.2191 | 0.2176 | 0.0019 | 0.0574 |
| *Streptococcus oralis* | Lactoferrin | 0.2182 | 0.2176 | 0.0008 | 0.0529 |
| *Streptococcus oralis* | Mn | 0.2179 | 0.2176 | 0.0004 | 0.0514 |
| *Streptococcus oralis* | I | 0.2176 | 0.2176 | 0.0001 | 0.0503 |
| *Haemophilus haemolyticus* | Mn | 0.1503 | 0.0188 | 0.1547 | 0.6174 |
| *Haemophilus haemolyticus* | Fe | 0.0894 | 0.0188 | 0.0775 | 0.3592 |
| *Haemophilus haemolyticus* | Lysozyme | 0.0850 | 0.0188 | 0.0723 | 0.3391 |
| *Haemophilus haemolyticus* | sIgA | 0.0751 | 0.0188 | 0.0608 | 0.2938 |
| *Haemophilus haemolyticus* | Se | 0.0728 | 0.0188 | 0.0582 | 0.2834 |
| *Haemophilus haemolyticus* | P | 0.0408 | 0.0188 | 0.0229 | 0.1400 |
| *Haemophilus haemolyticus* | Mg | 0.0296 | 0.0188 | 0.0111 | 0.0930 |
| *Haemophilus haemolyticus* | Zn | 0.0280 | 0.0188 | 0.0094 | 0.0863 |
| *Haemophilus haemolyticus* | Ca | 0.0244 | 0.0188 | 0.0057 | 0.0718 |
| *Haemophilus haemolyticus* | Lactose | 0.0215 | 0.0188 | 0.0027 | 0.0602 |
| *Haemophilus haemolyticus* | Mo | 0.0210 | 0.0188 | 0.0022 | 0.0585 |
| *Haemophilus haemolyticus* | Cu | 0.0195 | 0.0188 | 0.0007 | 0.0527 |
| *Haemophilus haemolyticus* | Lactoferrin | 0.0193 | 0.0188 | 0.0005 | 0.0519 |
| *Haemophilus haemolyticus* | K | 0.0191 | 0.0188 | 0.0003 | 0.0512 |
| *Haemophilus haemolyticus* | Na | 0.0189 | 0.0188 | 0.0001 | 0.0504 |
| *Haemophilus haemolyticus* | I | 0.0189 | 0.0188 | 0.0000 | 0.0501 |
| *Veillonella nakazawae* | Lysozyme | 0.1816 | 0.0999 | 0.0997 | 0.4421 |
| *Veillonella nakazawae* | sIgA | 0.1543 | 0.0999 | 0.0643 | 0.3077 |
| *Veillonella nakazawae* | Fe | 0.1498 | 0.0999 | 0.0587 | 0.2853 |
| *Veillonella nakazawae* | Se | 0.1346 | 0.0999 | 0.0400 | 0.2097 |
| *Veillonella nakazawae* | Na | 0.1315 | 0.0999 | 0.0363 | 0.1947 |
| *Veillonella nakazawae* | Mn | 0.1305 | 0.0999 | 0.0351 | 0.1898 |
| *Veillonella nakazawae* | Lactoferrin | 0.1225 | 0.0999 | 0.0257 | 0.1516 |
| *Veillonella nakazawae* | Lactose | 0.1143 | 0.0999 | 0.0162 | 0.1133 |
| *Veillonella nakazawae* | Ca | 0.1087 | 0.0999 | 0.0098 | 0.0878 |
| *Veillonella nakazawae* | I | 0.1051 | 0.0999 | 0.0058 | 0.0721 |
| *Veillonella nakazawae* | P | 0.1045 | 0.0999 | 0.0051 | 0.0694 |
| *Veillonella nakazawae* | Mg | 0.1020 | 0.0999 | 0.0023 | 0.0589 |
| *Veillonella nakazawae* | Zn | 0.1013 | 0.0999 | 0.0015 | 0.0559 |
| *Veillonella nakazawae* | Mo | 0.1008 | 0.0999 | 0.0010 | 0.0536 |
| *Veillonella nakazawae* | Cu | 0.1000 | 0.0999 | 0.0001 | 0.0503 |
| *Veillonella nakazawae* | K | 0.0999 | 0.0999 | 0.0000 | 0.0500 |
| *Staphylococcus lugdunensis* | Lactoferrin | 0.3298 | 0.2230 | 0.1593 | 0.6301 |
| *Staphylococcus lugdunensis* | I | 0.2732 | 0.2230 | 0.0690 | 0.3264 |
| *Staphylococcus lugdunensis* | Lysozyme | 0.2544 | 0.2230 | 0.0421 | 0.2183 |
| *Staphylococcus lugdunensis* | Mo | 0.2528 | 0.2230 | 0.0398 | 0.2088 |
| *Staphylococcus lugdunensis* | Na | 0.2378 | 0.2230 | 0.0194 | 0.1260 |
| *Staphylococcus lugdunensis* | Cu | 0.2358 | 0.2230 | 0.0168 | 0.1154 |
| *Staphylococcus lugdunensis* | Zn | 0.2352 | 0.2230 | 0.0160 | 0.1122 |
| *Staphylococcus lugdunensis* | Ca | 0.2341 | 0.2230 | 0.0144 | 0.1060 |
| *Staphylococcus lugdunensis* | K | 0.2335 | 0.2230 | 0.0136 | 0.1030 |
| *Staphylococcus lugdunensis* | Lactose | 0.2310 | 0.2230 | 0.0104 | 0.0902 |
| *Staphylococcus lugdunensis* | P | 0.2283 | 0.2230 | 0.0069 | 0.0764 |
| *Staphylococcus lugdunensis* | Mg | 0.2277 | 0.2230 | 0.0060 | 0.0730 |
| *Staphylococcus lugdunensis* | Mn | 0.2256 | 0.2230 | 0.0033 | 0.0625 |
| *Staphylococcus lugdunensis* | sIgA | 0.2238 | 0.2230 | 0.0011 | 0.0540 |
| *Staphylococcus lugdunensis* | Se | 0.2236 | 0.2230 | 0.0007 | 0.0527 |
| *Staphylococcus lugdunensis* | Fe | 0.2235 | 0.2230 | 0.0007 | 0.0526 |
| *Haemophilus parainfluenzae* | Mn | 0.1074 | 0.0735 | 0.0380 | 0.2016 |
| *Haemophilus parainfluenzae* | Na | 0.0946 | 0.0735 | 0.0234 | 0.1419 |
| *Haemophilus parainfluenzae* | Cu | 0.0916 | 0.0735 | 0.0200 | 0.1283 |
| *Haemophilus parainfluenzae* | Lactoferrin | 0.0908 | 0.0735 | 0.0191 | 0.1246 |
| *Haemophilus parainfluenzae* | Mo | 0.0879 | 0.0735 | 0.0159 | 0.1118 |
| *Haemophilus parainfluenzae* | Ca | 0.0842 | 0.0735 | 0.0117 | 0.0953 |
| *Haemophilus parainfluenzae* | P | 0.0838 | 0.0735 | 0.0113 | 0.0937 |
| *Haemophilus parainfluenzae* | Lysozyme | 0.0825 | 0.0735 | 0.0099 | 0.0881 |
| *Haemophilus parainfluenzae* | sIgA | 0.0810 | 0.0735 | 0.0082 | 0.0817 |
| *Haemophilus parainfluenzae* | K | 0.0777 | 0.0735 | 0.0046 | 0.0675 |
| *Haemophilus parainfluenzae* | Lactose | 0.0760 | 0.0735 | 0.0028 | 0.0606 |
| *Haemophilus parainfluenzae* | Fe | 0.0749 | 0.0735 | 0.0015 | 0.0557 |
| *Haemophilus parainfluenzae* | I | 0.0748 | 0.0735 | 0.0015 | 0.0556 |
| *Haemophilus parainfluenzae* | Zn | 0.0739 | 0.0735 | 0.0005 | 0.0519 |
| *Haemophilus parainfluenzae* | Se | 0.0735 | 0.0735 | 0.0001 | 0.0502 |
| *Haemophilus parainfluenzae* | Mg | 0.0735 | 0.0735 | 0.0000 | 0.0500 |
| Unknown *Flavobacteriaceae* sp. | sIgA | 0.1589 | 0.0969 | 0.0737 | 0.3446 |
| Unknown *Flavobacteriaceae* sp. | Zn | 0.1369 | 0.0969 | 0.0463 | 0.2354 |
| Unknown *Flavobacteriaceae* sp. | Mn | 0.1295 | 0.0969 | 0.0375 | 0.1993 |
| Unknown *Flavobacteriaceae* sp. | Ca | 0.1273 | 0.0969 | 0.0348 | 0.1883 |
| Unknown *Flavobacteriaceae* sp. | Lysozyme | 0.1246 | 0.0969 | 0.0316 | 0.1752 |
| Unknown *Flavobacteriaceae* sp. | Lactoferrin | 0.1186 | 0.0969 | 0.0246 | 0.1470 |
| Unknown *Flavobacteriaceae* sp. | Cu | 0.1124 | 0.0969 | 0.0175 | 0.1182 |
| Unknown *Flavobacteriaceae* sp. | Fe | 0.1124 | 0.0969 | 0.0175 | 0.1182 |
| Unknown *Flavobacteriaceae* sp. | Lactose | 0.1106 | 0.0969 | 0.0154 | 0.1101 |
| Unknown *Flavobacteriaceae* sp. | Mg | 0.1085 | 0.0969 | 0.0130 | 0.1004 |
| Unknown *Flavobacteriaceae* sp. | P | 0.1070 | 0.0969 | 0.0113 | 0.0935 |
| Unknown *Flavobacteriaceae* sp. | Mo | 0.1059 | 0.0969 | 0.0101 | 0.0890 |
| Unknown *Flavobacteriaceae* sp. | I | 0.1051 | 0.0969 | 0.0092 | 0.0853 |
| Unknown *Flavobacteriaceae* sp. | Na | 0.0979 | 0.0969 | 0.0010 | 0.0539 |
| Unknown *Flavobacteriaceae* sp. | Se | 0.0977 | 0.0969 | 0.0008 | 0.0531 |
| Unknown *Flavobacteriaceae* sp. | K | 0.0973 | 0.0969 | 0.0004 | 0.0516 |
| *Streptococcus parasanguinis* | Lysozyme | 0.2496 | 0.2014 | 0.0643 | 0.3076 |
| *Streptococcus parasanguinis* | Lactoferrin | 0.2343 | 0.2014 | 0.0430 | 0.2217 |
| *Streptococcus parasanguinis* | Se | 0.2342 | 0.2014 | 0.0428 | 0.2210 |
| *Streptococcus parasanguinis* | I | 0.2336 | 0.2014 | 0.0420 | 0.2177 |
| *Streptococcus parasanguinis* | Fe | 0.2334 | 0.2014 | 0.0417 | 0.2167 |
| *Streptococcus parasanguinis* | Lactose | 0.2292 | 0.2014 | 0.0361 | 0.1936 |
| *Streptococcus parasanguinis* | Cu | 0.2288 | 0.2014 | 0.0355 | 0.1914 |
| *Streptococcus parasanguinis* | P | 0.2258 | 0.2014 | 0.0316 | 0.1752 |
| *Streptococcus parasanguinis* | Ca | 0.2217 | 0.2014 | 0.0260 | 0.1527 |
| *Streptococcus parasanguinis* | K | 0.2151 | 0.2014 | 0.0174 | 0.1180 |
| *Streptococcus parasanguinis* | Zn | 0.2074 | 0.2014 | 0.0076 | 0.0792 |
| *Streptococcus parasanguinis* | Na | 0.2038 | 0.2014 | 0.0029 | 0.0612 |
| *Streptococcus parasanguinis* | Mn | 0.2037 | 0.2014 | 0.0028 | 0.0608 |
| *Streptococcus parasanguinis* | sIgA | 0.2023 | 0.2014 | 0.0011 | 0.0543 |
| *Streptococcus parasanguinis* | Mg | 0.2018 | 0.2014 | 0.0004 | 0.0516 |
| *Streptococcus parasanguinis* | Mo | 0.2016 | 0.2014 | 0.0002 | 0.0506 |
| *Bifidobacterium longum* | I | 0.2892 | 0.1900 | 0.1396 | 0.5738 |
| *Bifidobacterium longum* | Lactoferrin | 0.2528 | 0.1900 | 0.0840 | 0.3842 |
| *Bifidobacterium longum* | Zn | 0.2261 | 0.1900 | 0.0468 | 0.2371 |
| *Bifidobacterium longum* | Lysozyme | 0.2215 | 0.1900 | 0.0405 | 0.2116 |
| *Bifidobacterium longum* | Lactose | 0.2192 | 0.1900 | 0.0374 | 0.1991 |
| *Bifidobacterium longum* | Mn | 0.2110 | 0.1900 | 0.0266 | 0.1551 |
| *Bifidobacterium longum* | Se | 0.2077 | 0.1900 | 0.0223 | 0.1378 |
| *Bifidobacterium longum* | Fe | 0.2064 | 0.1900 | 0.0207 | 0.1313 |
| *Bifidobacterium longum* | Na | 0.2044 | 0.1900 | 0.0182 | 0.1210 |
| *Bifidobacterium longum* | Mg | 0.2034 | 0.1900 | 0.0169 | 0.1160 |
| *Bifidobacterium longum* | P | 0.1968 | 0.1900 | 0.0086 | 0.0830 |
| *Bifidobacterium longum* | K | 0.1934 | 0.1900 | 0.0042 | 0.0661 |
| *Bifidobacterium longum* | Mo | 0.1934 | 0.1900 | 0.0042 | 0.0661 |
| *Bifidobacterium longum* | sIgA | 0.1934 | 0.1900 | 0.0042 | 0.0660 |
| *Bifidobacterium longum* | Cu | 0.1906 | 0.1900 | 0.0008 | 0.0531 |
| *Bifidobacterium longum* | Ca | 0.1904 | 0.1900 | 0.0006 | 0.0522 |
| *Lactobacillus gasseri* | Lactoferrin | 0.0929 | 0.0418 | 0.0563 | 0.2758 |
| *Lactobacillus gasseri* | sIgA | 0.0778 | 0.0418 | 0.0391 | 0.2058 |
| *Lactobacillus gasseri* | I | 0.0744 | 0.0418 | 0.0352 | 0.1902 |
| *Lactobacillus gasseri* | Mo | 0.0735 | 0.0418 | 0.0342 | 0.1861 |
| *Lactobacillus gasseri* | Zn | 0.0708 | 0.0418 | 0.0313 | 0.1741 |
| *Lactobacillus gasseri* | Cu | 0.0677 | 0.0418 | 0.0279 | 0.1602 |
| *Lactobacillus gasseri* | Lysozyme | 0.0559 | 0.0418 | 0.0150 | 0.1084 |
| *Lactobacillus gasseri* | Mn | 0.0511 | 0.0418 | 0.0099 | 0.0881 |
| *Lactobacillus gasseri* | Lactose | 0.0503 | 0.0418 | 0.0090 | 0.0847 |
| *Lactobacillus gasseri* | K | 0.0486 | 0.0418 | 0.0072 | 0.0777 |
| *Lactobacillus gasseri* | Fe | 0.0479 | 0.0418 | 0.0065 | 0.0748 |
| *Lactobacillus gasseri* | Ca | 0.0466 | 0.0418 | 0.0051 | 0.0696 |
| *Lactobacillus gasseri* | P | 0.0440 | 0.0418 | 0.0024 | 0.0590 |
| *Lactobacillus gasseri* | Mg | 0.0431 | 0.0418 | 0.0014 | 0.0555 |
| *Lactobacillus gasseri* | Na | 0.0420 | 0.0418 | 0.0003 | 0.0510 |
| *Lactobacillus gasseri* | Se | 0.0418 | 0.0418 | 0.0000 | 0.0501 |
| Shannon diversity | Lactoferrin | 0.3769 | 0.2175 | 0.2559 | 0.8276 |
| Shannon diversity | I | 0.3080 | 0.2175 | 0.1308 | 0.5466 |
| Shannon diversity | Na | 0.3069 | 0.2175 | 0.1290 | 0.5410 |
| Shannon diversity | Ca | 0.3035 | 0.2175 | 0.1235 | 0.5233 |
| Shannon diversity | K | 0.2872 | 0.2175 | 0.0978 | 0.4350 |
| Shannon diversity | P | 0.2647 | 0.2175 | 0.0642 | 0.3074 |
| Shannon diversity | Cu | 0.2598 | 0.2175 | 0.0571 | 0.2791 |
| Shannon diversity | Zn | 0.2575 | 0.2175 | 0.0539 | 0.2662 |
| Shannon diversity | Lactose | 0.2450 | 0.2175 | 0.0365 | 0.1953 |
| Shannon diversity | sIgA | 0.2446 | 0.2175 | 0.0359 | 0.1929 |
| Shannon diversity | Fe | 0.2445 | 0.2175 | 0.0359 | 0.1927 |
| Shannon diversity | Se | 0.2353 | 0.2175 | 0.0233 | 0.1416 |
| Shannon diversity | Mg | 0.2319 | 0.2175 | 0.0188 | 0.1234 |
| Shannon diversity | Lysozyme | 0.2315 | 0.2175 | 0.0182 | 0.1212 |
| Shannon diversity | Mn | 0.2314 | 0.2175 | 0.0181 | 0.1209 |
| Shannon diversity | Mo | 0.2213 | 0.2175 | 0.0049 | 0.0687 |
| Richness | Lactoferrin | 0.3324 | 0.2509 | 0.1221 | 0.5187 |
| Richness | Na | 0.3175 | 0.2509 | 0.0976 | 0.4344 |
| Richness | Ca | 0.3065 | 0.2509 | 0.0802 | 0.3698 |
| Richness | I | 0.3020 | 0.2509 | 0.0732 | 0.3427 |
| Richness | Zn | 0.2934 | 0.2509 | 0.0603 | 0.2916 |
| Richness | K | 0.2871 | 0.2509 | 0.0508 | 0.2534 |
| Richness | P | 0.2757 | 0.2509 | 0.0343 | 0.1864 |
| Richness | Lactose | 0.2732 | 0.2509 | 0.0308 | 0.1720 |
| Richness | Fe | 0.2717 | 0.2509 | 0.0286 | 0.1631 |
| Richness | Cu | 0.2714 | 0.2509 | 0.0282 | 0.1614 |
| Richness | sIgA | 0.2699 | 0.2509 | 0.0261 | 0.1529 |
| Richness | Lysozyme | 0.2651 | 0.2509 | 0.0193 | 0.1258 |
| Richness | Mg | 0.2642 | 0.2509 | 0.0181 | 0.1206 |
| Richness | Mn | 0.2638 | 0.2509 | 0.0175 | 0.1185 |
| Richness | Se | 0.2605 | 0.2509 | 0.0130 | 0.1005 |
| Richness | Mo | 0.2509 | 0.2509 | 0.0000 | 0.0501 |

R²_full: Coefficient of determination from the full model including the milk component and covariates; R²_covariate: Coefficient of determination from the covariate-adjusted model excluding the milk component; F² = effect size.

Supplementary figure:

**Supplementary Figure S1.** PCoA plots of Aitchison distances for infant oral microbiome samples at 3 months of age. (A) Overall community structure across all samples showing even clustering with no outliers. (B) Samples colored by DNA extraction / PCR batch, showing no significant influence of processing batch on microbiome profiles (PERMANOVA P=0.608). (C) Samples colored by sequencing pool, showing no significant influence of sequencing bacth on microbiome profiles (PERMANOVA P=0.533).
